# Targeting AXL induces tumor-intrinsic immunogenic response in tyrosine kinase inhibitor-resistant liver cancer

**DOI:** 10.1038/s41419-024-06493-0

**Published:** 2024-02-03

**Authors:** Yunong Xie, Haofeng Wu, Yimiao He, Linglin Liu, Ianto Bosheng Huang, Lei Zhou, Cheuk-Yin Lin, Rainbow Wing-Hei Leung, Jia-Jian Loh, Terence Kin-Wah Lee, Jin Ding, Kwan Man, Stephanie Ma, Man Tong

**Affiliations:** 1 School of Biomedical Sciences, The Chinese University of Hong Kong, Hong Kong, China; 2 School of Biomedical Sciences, Li Ka Shing Faculty of Medicine, The University of Hong Kong, Hong Kong, China; 3 Precision Medicine Institute, The First Affiliated Hospital, Sun Yat-Sen University, Guangzhou, China; 4 Department of Applied Biology and Chemical Technology, The Hong Kong Polytechnic University, Hong Kong, China; 5 Clinical Cancer Institute, Center for Translational Medicine, Naval Medical University, Shanghai, China; 6 Department of Surgery, School of Clinical Medicine, Li Ka Shing Faculty of Medicine, The University of Hong Kong, Hong Kong, China; 7 State Key Laboratory of Liver Research, The University of Hong Kong, Hong Kong, China; 8 Hong Kong University—Shenzhen Hospital, Shenzhen, China

**Keywords:** Cancer therapeutic resistance, Liver cancer

## Abstract

Hepatocellular carcinoma (HCC) is an aggressive malignancy without effective therapeutic approaches. Here, we evaluate the tumor-intrinsic mechanisms that attenuate the efficacy of immune checkpoint inhibitor (ICI) that is observed in patients with advanced HCC who progress on first-line tyrosine kinase inhibitor (TKI) therapy. Upregulation of AXL observed in sorafenib- and lenvatinib-resistant HCCs is correlated with poor response towards TKI and ICI treatments. AXL upregulation protects sorafenib-resistant HCC cells from oxidative stress, mitochondrial damage, and accompanying immunogenic cell death through suppressed tumor necrosis factor-α (TNF-α) and STING-type I interferon pathways. Pharmacological inhibition of AXL abrogates the protective effect and re-sensitizes TKI-resistant HCC tumors to anti-PD-1 treatment. We suggest that targeting AXL in combination with anti-PD-1 may provide an alternative treatment scheme for HCC patients who progress on TKI treatment.

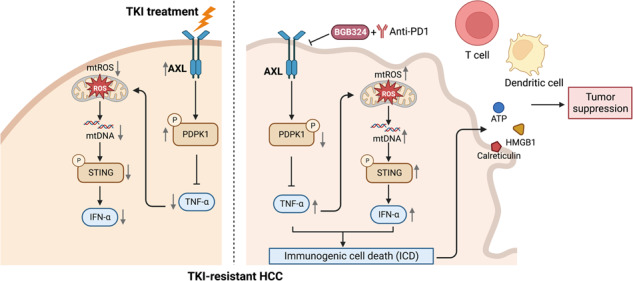

## Introduction

Tyrosine kinase inhibitors (TKI) and immune checkpoint inhibitors (ICI) are an integral part of systemic therapy in advanced hepatocellular carcinoma (HCC). Despite various TKIs and ICI-based therapy being approved for first-line or second-line therapy in HCC, the best treatment sequences of the available drugs have not been established. Sorafenib and lenvatinib are the FDA-approved first-line TKIs for advanced HCC patients. For HCC patients who are not responsive or progress on first-line TKI, TKIs such as regorafenib and cabozantinib and ICI-based therapy targeting PD-1 and CTLA-4 are approved as second-line treatment [1]. Subsequent therapy using drugs with distinct mechanisms of action is anticipated to generate optimal therapeutic effects in cancer treatment. However, compromised therapeutic efficacy of second-line ICI-based therapy has been witnessed in cancers displaying immune-evasive phenotypes following first-line targeted therapy [2, 3]. In HCC, several clinical trials revealed that the survival benefits of second-line ICI treatment were not improved in HCC patients who progressed on TKI treatment when compared with treatment-naïve patients (CheckMate 040 and KEYNOTE-224). Yet, the underlying mechanisms that cause poor response to ICI-based therapy in TKI-resistant HCC remain elusive.

AXL is a receptor tyrosine kinase that belongs to the TAM receptor family [4]. The oncogenic functions of AXL have been extensively characterized in different solid tumors, including HCC [5–7]. Previous studies have also revealed a critical role of AXL in contributing to drug resistance towards various anti-cancer therapies such as chemotherapy and targeted therapy [8]. Accumulating evidence suggests that AXL activation shapes an immunosuppressive tumor microenvironment [9–11]. Notably, clinical trials have been conducted to investigate the therapeutic potential and safety profile of combined treatment of AXL inhibition and ICI in solid tumors [12]. The role of AXL in sorafenib-resistant HCC was first reported in a previous study, which showed that upregulation of AXL promoted the epithelial-to-mesenchymal transition pathway and thus increased the motility of sorafenib-resistant HCC cells [13]. However, the immunosuppressive role of AXL that affects TKI and ICI treatment responses has not been documented in HCC. In this study, we found that TKI-resistant HCC displayed an immunosuppressive phenotype, which was regulated by AXL-dependent suppression of proinflammatory signaling. Pharmacological inhibition of AXL could overcome TKI resistance by inducing immunogenic cell death and re-sensitizing TKI-resistant HCC towards ICI therapy.

## Materials and methods

### Animal experiments

All study protocols were approved by and performed in accordance with the Committee on the Use of Live Animals in Teaching and Research at The University of Hong Kong, the Animal Experimentation Ethics Committee at The Chinese University of Hong Kong, and the Animals (Control of Experiments) Ordinance of Hong Kong. For subcutaneous xenograft model, HCC cells resuspended in Matrigel (Corning, # 354234) at 1:1 ratio were injected subcutaneously into the flank of male C57BL/6 N mice. Tumor volumes were measured every two days with the caliper and calculated using the following formula: volume (cm^3^) = L × W^2^ × 0.5, with *L* and *W* representing the largest and smallest diameters, respectively. Drug administration began when the tumors reached 50 mm^3^ and mice were randomized for treatment. For the hydrodynamic tail vein injection model, 6–8-week-old male C57BL/6 N mice were injected through lateral tail vein with 22.5 µg of plasmids encoding human AKT1 (myristylated AKT1) and human neuroblastoma Ras viral oncogene homolog (N-RasV12) along with sleeping beauty transposase in a ratio of 25:1, diluted in 2 ml saline (0.9% NaCl), filtered through 0.22 µm filter. Mice were subjected to sorafenib (30 mg/kg/day, daily, p.o.), lenvatinib (30 mg/kg/day, daily, p.o.), BGB324 (10 mg/kg/day, daily, p.o.) and/or anti-PD-1 treatment (5 mg/kg, twice/week, i.p.), and H-151 (750 nmol in 200 µL PBS with 5% Tween 80/mice, daily, i.p.). There was no exclusion of animals, and the experimenter was not blinded to the assignment of the groups and the evaluation of the results. No statistical methods were used for sample size estimation.

### Clinical samples

Tissue microarray (TMA) comprising 89 HCC tissue samples was collected from HCC patients who received surgical resection at the Eastern Hepatobiliary Surgical Hospital from December 2008 to May 2010. Informed consent was obtained from the patients. The patients did not receive any previous local or systemic treatment prior to the operation. Sorafenib was administered as first-line targeted therapy to these patients at a dose of 400 mg, twice a day. The study was approved by the Ethical Committee of the Eastern Hepatobiliary Surgical Hospital. Clinico-pathological features of these patients were reported previously [14].

### Cell lines and HCC organoids

Human HCC cell line HepG2 was purchased from the American Type Culture Collection. Human HCC cell line PLC/PRF/5 were purchased from the Japanese Collection of Research Bioresources. The establishment of sorafenib-resistant clones from HepG2 and PLC/PRF/5 cells was reported previously [15, 16]. Murine hepatic cancer cell line RIL-175 was a gift from Dr. Lars Zender (University of Tübingen, Tübingen, Germany). 293FT cells were purchased from Invitrogen. Cells were routinely inspected for any mycoplasma contamination by PCR method. The source and culture conditions of HCC organoids were reported previously [17].

### Publicly available datasets and bioinformatics analyses

#### Human clinical samples

The mRNA expression of 67 sorafenib-treated HCC patients (GSE109211) was downloaded from the Gene Expression Omnibus (GEO) database of the National Centre for Biotechnology Information (NCBI). There were 21 sorafenib treatment responders and 46 non-responders. HCC tissue samples were segregated into two groups (AXL-high and AXL-low) using the median AXL expression levels as a cut-off point. The correlation of AXL expression and clinical sorafenib response was calculated by Chi-square contingency analysis. HCC patient transcriptomes were downloaded from The Cancer Genome Atlas - Liver Hepatocellular Carcinoma (TCGA-LIHC) cohort using TCGABiolinks (v2.14) and normalized with DESeq2 (v1.26). TCGA-LIHC tumor samples were purified by the ESTIMATE tumor purity index [18]. HCC patients were divided into AXL-high and AXL-low groups based on their median transcriptomic AXL expression level. Tumor Immune Dysfunction and Exclusion (TIDE) analysis was performed using the web-based TIDE analytical tool (http://tide.dfci.harvard.edu/). Patient stratification with immunotherapy response and calculations of the immune dysfunction score and the immune exclusion score were performed using this tool. The heatmap was drawn by the pheatmap package of R.

#### HCC cell lines/patient-derived xenografts

Our previously reported transcriptomic datasets of sorafenib-sensitive/resistant HepG2 cells and lenvatinib-sensitive/resistant patient-derived xenograft were deposited at GSE108531 and GSE191224, respectively. The druggable gene targets in the TKI-resistant samples were identified by the Drug Gene Interaction Database (DGIdb, https://www.dgidb.org/), and the top-ranked targets were displayed by the heatmap showing the fold-change of gene expression in resistant versus sensitive samples. GEO datasets (GSE176151 and GSE151412) containing transcriptomic data of sorafenib-sensitive and resistant HCC cell lines were downloaded from GREIN (http://www.ilincs.org/apps/grein/?gse=). The tumor-infiltrating immune cell abundance was calculated by performing the Pathway Level analysis of Gene Expression (PLAGE) score using the GSVA R package (version 4.2.1) on the ConsensusTME LIHC gene signature. The Pearson correlation matrix was calculated and plotted by corrplot package of R.

#### Gene set enrichment analysis (GSEA)

GSEA was performed using the GSEA_MSigDB software (version 4.1.0). An interferon-stimulated gene (ISG) signature gene set containing 71 genes (Reactome: Interferon Alpha Beta Signaling) was applied as a self-defined gene set for GSEA.

### Drugs and recombinant proteins

Sorafenib (S-8502) and lenvatinib (L-5400) were purchased from LC Laboratories. AXL inhibitor BGB324 (V0635) was purchased from InvivoChem. Anti-PD-1 antibody (BE0146) and its IgG2a isotype control (BE0089) were purchased from BioXCell. Human TNFα (HZ-1014) was purchased from ProteinTech. Human IFNα (PHC4044) was purchased from Thermo Fisher. mitoTEMPO (1569257-94-80) was purchased from SantaCruz. STING inhibitor H-151 (HY-112693) was purchased from MedChemExpress (MCE).

### Plasmids and lentiviral transduction

Human AXL-specific (NM_021913) and human PDPK1-specific (NM_002610) shRNA expression vectors and scrambled shRNA non-target control (NTC) (pLKO.1-puro) were purchased from Sigma-Aldrich. shRNA sequences are available in Supplemental Table 1. Plasmids were transfected into 293FT cells and packaged using MISSION Lentiviral Packaging Mix (Sigma-Aldrich). Transduced cells were selected using puromycin.

### RNA extraction, cDNA synthesis, and quantitative real-time PCR

Total mRNAs were extracted using RNA IsoPlus (TaKaRa), and cDNA was synthesized using PrimeScript^TM^ RT Master Mix (TaKaRa). Gene expression was detected with primers listed in Supplemental Table 1 and real-time qPCR was performed and analyzed using Roche LightCycler 480 system (Roche).

### Western blotting

Protein was extracted from cells using 1× RIPA buffer (Cell Signaling) supplemented with protease and phosphatase inhibitors. Nuclear sub-fractionation was performed according to the previous protocol [19]. Proteins were quantified and resolved on an SDS-PAGE gel, transferred onto a PVDF membrane (Millipore), and immunoblotted with primary antibody, followed by incubation with secondary antibody. Antibody signal was detected using an enhanced chemiluminescence system (Cytiva). Antibodies used are listed in Supplemental Table 2. For measurement of secretary HMGB1, culture medium was collected and spun down at 2000 × *g* for 5 min to remove any cell debris. Collected medium was denatured with SDS denaturing buffer. Western blotting was performed as described above.

### Enzyme-linked immunosorbent assay (ELISA)

To measure the secretory TNF-α and IFN-α, conditioned cell media were collected and centrifuged at 2000 × *g* for 5 min. The supernatant was collected, and ELISA assays were performed according to the manufacturer’s instructions (Invitrogen BMS216 for IFN-α and Invitrogen 88-7346-22 for TNF-α).

### Cytoplasmic mitochondrial DNA quantification

To measure the cytosolic mitochondrial DNA (mtDNA) leakage, cells were trypsinized and collected at 200 × *g* for 5 min and divided into two equal parts. One part was subjected to DNA extraction following the instruction of the QIAamp DNA kit (Qiagen) and served as the normalization control for the total mtDNA. The other part of the cells was resuspended in 200 µl of buffer containing 150 mM NaCl, 50 mM HEPES-KOH (pH 7.4), and 25 µg/ml digitonin (Calbiochem). The homogenates were incubated end-over-end for 10 min and centrifuged at 1000 × *g* for 3 min. Centrifugation was repeated three times to clear the supernatant of intact cells. The cytosolic fractions were spun down at 17,000 × *g* for 10 min, and DNA was isolated from the supernatant using the QIAamp DNA kit (Qiagen). Real-time qPCR was performed on cytosolic fractions using mtDNA primers (mt16S, mtCytb, mtDloop). Neglectable level of nuclear DNA in cytosolic fractions using nuclear β-actin DNA primer, indicating no nuclear lysis happened during the extraction process.

### Extracellular ATP quantification

After treatment, the conditioned medium was collected and spun at 2000 × *g* for 5 min to eliminate any cell debris. The level of ATP was determined using the ATP determination kit (Thermo Fisher, A22066) following the manufacturer’s protocol.

### Flow cytometry analyses

For measuring mitochondrial ROS, cells were stained with MitoSOX red mitochondrial superoxide dye (Thermo Scientific) at the concentration suggested by the manufacturer for 30 min at 37 °C. For calreticulin staining, cells were stained with calreticulin antibody (1 μg/ml) (Abcam, ab92516) for 30 min at RT, followed by staining with Alexa Fluoro 488 goat-anti-rabbit antibody (Invitrogen) for 30 min at RT. Stained cells were analyzed on BD FACSCanto II (BD Biosciences) with data analyzed by FlowJo (Tree Star). Residual tumors were resected from mice. Dissociation of tumor tissues into single cells was performed according to our previous protocol [20]. Dissociated single cells were stained with LIVE/DEAD Fixable Red Dead Cell Stain (Invitrogen, L34972) and antibodies of immune markers listed in Supplemental Table 2. Immunophenotyping was performed on Quanteon Flow Cytometer (Novocyte) and FACSymphony A5.2 (BD), and data analysis was performed using Flowjo (Tree Star). Gating strategies were included in Suppl. Fig. 19.

### Immunohistochemistry and TMA analysis

Immunohistochemical staining of paraffin sections was carried out using a two-step protocol. Slides were immersed in antigen retrieval buffer and heated using boiling water. Endogenous peroxidase activity was inhibited with 3% hydrogen peroxide. Sections were subsequently incubated overnight with primary antibodies at 4 °C. Primary antibodies were listed in Supplemental Table 2. Slides were developed with DAB+ Substrate-Chromogen System (Dako) and counterstained with Mayer’s hematoxylin. Quantification of staining densities of the immunohistochemistry images was performed using ImageJ (v1.8.0_112). Three random fields were selected for quantification. TMA on the archived 89 clinical patient samples was stained with AXL antibody. AXL expression was scored and separated into AXL-low, AXL-medium, and AXL-high groups according to the percentage and intensity of staining described in the previous publications [21].

### Immunofluorescence

For immunofluorescence staining of mitoTracker Green, BAX, and BAK, cells were seeded on glass coverslips, and stained with mitoTracker Green (M7157, Invitrogen) according to the manufacturer’s instruction at 37 °C for 30 min. Cells were then fixed in 4% paraformaldehyde (Sigma-Aldrich), and permeabilized with 0.1% Triton X-100 solution (Sigma-Aldrich). Non-specific binding sites were blocked with 5% bovine serum albumin solution in phosphate-buffered saline (PBS). Primary antibodies used for immunoblotting against various targets were listed as follows: BAX (1:50, 5023; Merck Cell Signaling Technology), BAK (1:100, 12105; Cell Signaling Technology). Cells were counterstained with antifade 4′,6-diamidino-2-phenylindole (DAPI, Invitrogen) and visualized by a fluorescent confocal microscope (FV1200; Olympus). For multiplex immunofluorescence staining, each target was first optimized for conditions by chromogen-based IHC before multiplex immunofluorescence using Opal 4-Color Manual IHC Kit (Akoya Biosciences, #NEL810001KT). Sections were deparaffinized in xylene and rehydrated in decreasing graded alcohols and distilled water. Slides were processed for antigen retrieval by a standard microwave heating technique in diluted 50× Envision FLEX Target Retrieval Buffer (pH 9.0, K8004, Dako) for 15 min. Endogenous peroxidase activities were quenched using 3% hydrogen peroxide for 10 min at room temperature. The sections were immersed in blocking/antibody diluent (Akoya Biosciences, ARD1001EA) for 30 min at room temperature. Specimens were incubated with primary antibodies (AXL (1:500, Abcam, ab227871); CD8α (1:500, Abcam, ab217344); CD103 (1:50, R&D System, AF1990)). The sections were then washed thoroughly and incubated with Opal polymer HRP Ms+Rb (ARH1001EA, Akoya Biosciences) for 30 min at room temperature. Followed by a brief wash with 1× TBST, Opal fluorophore (1:100) was applied for AXL, (Opal 570), CD103, (Opal 520), CD8α, (Opal 690) for 15 min at room temperature. A final stripping step was performed in 1× AR6 sodium citrate buffer (pH 6.0) in a microwave oven for 15 min. The section slides were cooled down, counterstained with DAPI solution (1:1000, AKOYA), and imaged using Vectra Polaris imaging system (PerkinElmer).

### Statistical analyses

Statistical analyses were performed using GraphPad Prism 9.0 and SPSS 21.0. Data were analyzed by a two-tailed unpaired Student’s *t* test (2 groups), or one-way ANOVA (>2 groups) with Bonferroni’s multiple comparisons test. Survival curves were plotted by the Kaplan–Meier method and the statistical *p* values were generated by the Cox-Mantel log-rank test. All in vitro functional assays are a representation of at least three independent experiments expressed as mean ± SEM. Statistical significance was defined as *p* ≤ 0.05. Significance values were set at *p ≤ 0.05, **p < 0.01, ***p < 0.001 and ****p < 0.0001.

## Results

### TKI-resistant HCC is negatively associated with immune-related signatures and show altered infiltration of immune cells

**Fig. 1:**
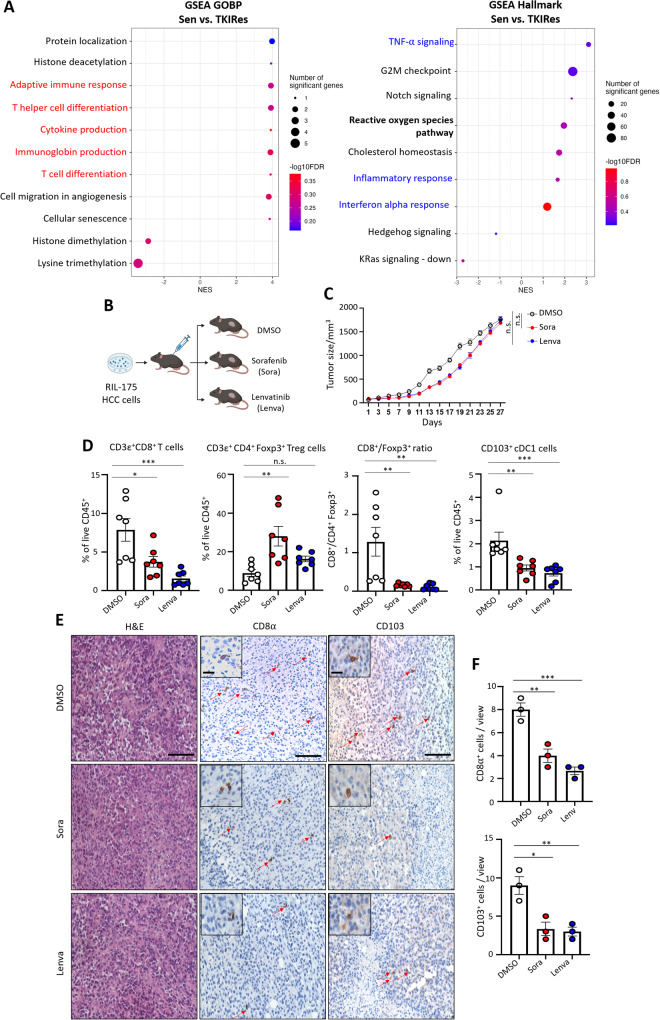
TKI-resistant HCC is negatively associated with immune-related signatures and show altered infiltration of immune cells. **A** Gene Set Enrichment Analysis (GSEA) of TKI sensitive (Sen) versus TKI-resistant (TKIRes) samples; left - Gene Oncology Biological Process (GOBP) and right - Hallmark. **B** Schematic of sorafenib (Sora), lenvatinib (Lenva) and vehicle control (DMSO) treatment in C57BL/6 N mice bearing xenografts established from murine HCC cell line RIL-175. **C** Tumor growth curves of C57BL/6 N mice bearing RIL-175 xenografts treated with Sora, Lenva, or DMSO. **D** Bar charts showing the percentages of intratumoral immune cell populations and CD8^+^/Foxp3^+^ ratio in mice treated with Sora, Lenva or DMSO (*n* = 7 per group). Data representative of two independent experiments. **E** Representative H&E and IHC images showing CD8α^+^ and CD103^+^ cells in the tumor sections treated with either Sora, Lenva, or DMSO. Scale bar = 100 µm and 25 µm (inset). Red arrows indicate positive signals of CD8α and CD103. **F** Bar charts showing the quantification of CD8α^+^ and CD103^+^ cells in three independent, randomly selected fields. *p < 0.05; **p < 0.01; ***p < 0.001; n.s. not significant on one-way ANOVA with Bonferroni’s multiple comparisons test.

To identify dysregulated gene signatures shared between TKI-resistant HCCs with acquired sorafenib and lenvatinib resistance, transcriptomes of sorafenib and lenvatinib-resistant HCC cells and patient-derived xenograft (denoted as TKIRes) and their corresponding sensitive sample (denoted as Sen) established in our previous studies [15, 16] were subjected to Gene Set Enrichment Analysis (GSEA). GSEA results revealed that immune-associated processes (under Gene Ontology Biological Process, GOBP) were enriched in Sen samples when compared with TKIRes samples (Fig. 1A). Hallmark signatures associated with inflammatory responses, including TNF-α signaling and interferon alpha response, were also enriched in Sen samples when compared with TKIRes samples (Fig. 1A). In view of the negative association of the immune-associated and inflammatory signatures in TKI-resistant HCC, we modeled acquired sorafenib and lenvatinib resistance in immunocompetent mice bearing xenograft implantation established from murine HCC cells RIL-175 with continuous treatment of either sorafenib, lenvatinib or vehicle control (Fig. 1B). Tumor growth was delayed under the treatment of either sorafenib or lenvatinib, until the end of week 4 when the tumor sizes did not have significant difference between TKI-treated groups and control group (Fig. 1C and Suppl. Fig. 1A, B). Immunophenotyping of residual tumors showed that TKI treatment with either sorafenib or lenvatinib led to reduced tumor-infiltrating CD8^+^ T cells and increased immunosuppressive CD4^+^Foxp3^+^ regulatory T cells (Treg) (Fig. 1D). TNF-α and INF-γ-expressing CD8^+^ T cells were also suppressed in the tumor tissues after TKI treatment, despite no obvious change in the populations of CD8^+^granzyme B^+^ cells and CD8^+^PD-1^+^ cells (Suppl. Fig. 1C, D). The ratio of CD8^+^/Foxp3^+^ T cells, which is indicative of immunotherapy response [22], is greatly reduced in TKI-treated tumors (Fig. 1D). In addition, CD103^+^ conventional type 1 dendritic cells (cDC1) [23], which are potent antigen-presenting cells for the activation of CD8^+^ cytotoxic T cells were also reduced (Fig. 1D). Diminished infiltrations of CD8^+^ and CD103^+^ cells were further confirmed by immunohistochemistry in the tumor tissue sections (Fig. 1E, F). These data collectively suggest that TKI treatment may cause an attenuated immune response and landscape in both HCC cells and the tumor microenvironment.

### AXL upregulation in TKI-resistant HCC inversely correlates with tumor infiltration of immune cells and may predict treatment response

**Fig. 2:**
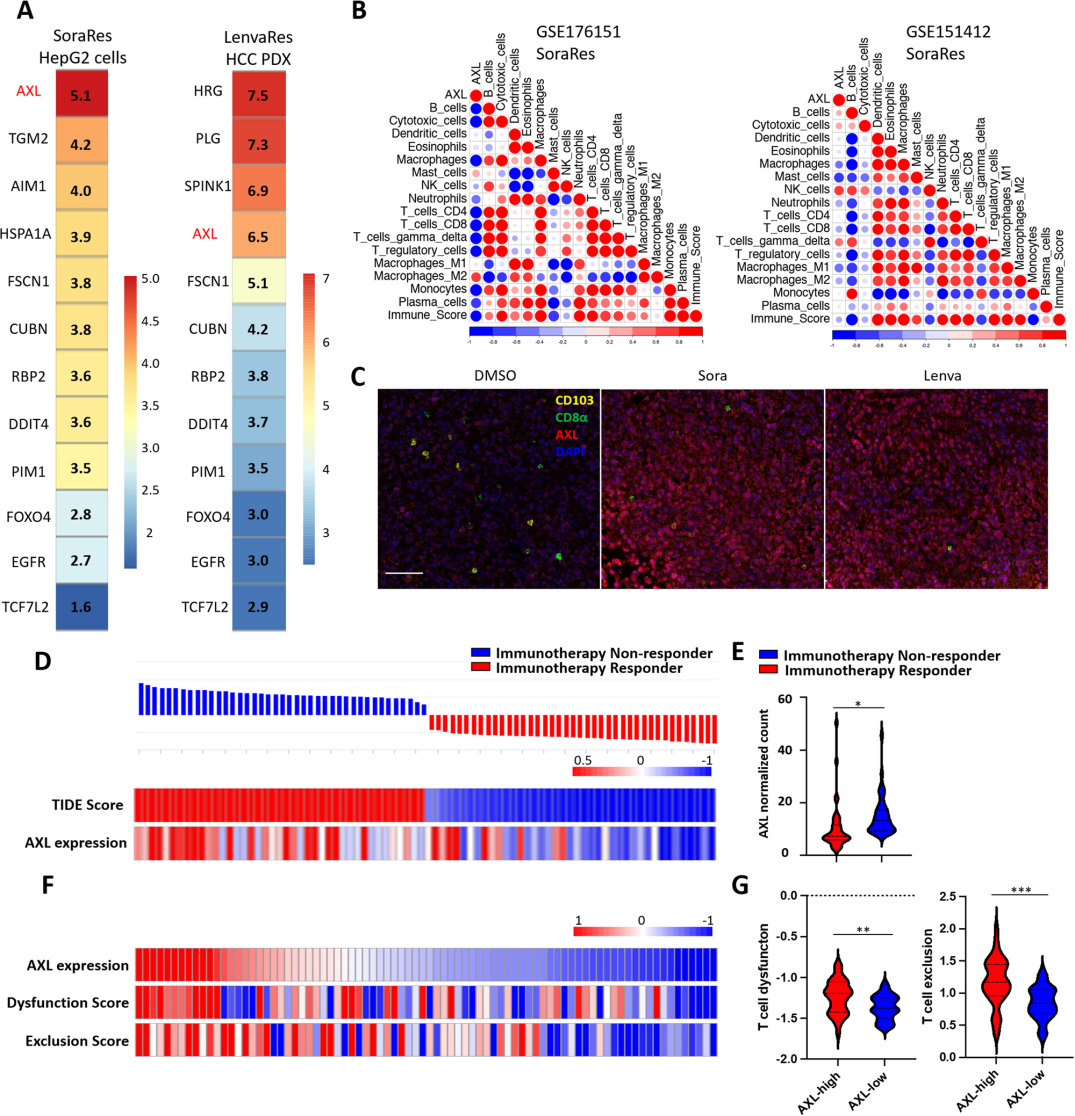
AXL upregulation in TKI-resistant HCC inversely correlates with tumor infiltration of immune cells and may predict treatment response. **A** Lists of upregulated druggable targets in sorafenib-resistant HepG2 cells and lenvatinib-resistant HCC patient-derived xenograft (PDX). The numbers and the color scale bar indicate the expression fold-change of resistant samples versus sensitive samples. **B** Bubble plots showing the correlation analysis of AXL with immune gene signatures in sorafenib-resistant samples from the datasets GSE176151 (left) and GSE151412 (right). The color scale bar and the size of the dots indicate the Pearson correlation coefficient. **C** Representative multiplex immunofluorescence images (top) of staining of CD103, CD8α, AXL, and DAPI in residual xenografts after treatment with DMOS, Sora, or Lenva. Scale bar = 50 µm. **D** Immunotherapy response prediction (top) of TCGA-LIHC patients using Tumor immune dysfunction and exclusion (TIDE) analysis. Heatmap (bottom) of TIDE scores and the corresponding AXL expression (normalized count) of patients from TCGA-LIHC dataset. **E** Violin plot of AXL expression in immunotherapy responders and non-responders as predicted by TIDE. **F** Heatmap showing AXL expression, T-cell dysfunction score, and T-cell exclusion score of patients from TCGA-LIHC dataset. **G** Violin plots of T-cell dysfunction score (left) and T-cell exclusion score (right) in AXL-high and AXL-low patients. *p < 0.05; **p < 0.01; ***p < 0.001 on a two-tailed unpaired Student’s *t* test.

With the aim of identifying the molecular determinants that contribute to the immunosuppressive phenotypes incurred in TKI-resistant HCC and developing novel therapies to overcome TKI resistance, we selected the druggable genes that are commonly upregulated in both sorafenib-resistant (SoraRes) and lenvatinib-resistant (LenvaRes) HCC transcriptomes. AXL was found to be the most upregulated druggable gene, which is commonly deregulated in both SoraRes and LenvaRes HCC samples (Fig. 2A). In other publicly available datasets, AXL is consistently upregulated in sorafenib-resistant HCC samples (Suppl. Fig. 2A). In a public dataset (GSE109211) comprising patients who are either sorafenib responders or non-responders, high AXL mRNA level was significantly correlated with poor response towards sorafenib treatment (Suppl. Fig. 2B). Using our in-house tissue microarray with tumor tissue sections obtained from HCC patients who went on for sorafenib treatment, high proteomic AXL expression was tightly associated with worse overall survival, indicative of a prognostic value of AXL in predicting sorafenib treatment response (Suppl. Fig. 2C, D).

We next asked whether AXL upregulation is negatively associated with immune signatures and may predict immunotherapy response in HCC. Comparing the GSVA scores of immune cell signatures from ConsensusTME [24] with AXL expression in sorafenib-sensitive and sorafenib-resistant HCC samples, we observed that AXL expression was negatively correlated with immune cell signatures including T lymphocytes in sorafenib-resistant HCC samples (Fig. 2B), whereas AXL expression is positively correlated with immune cell signatures in sorafenib-sensitive HCC samples (Suppl. Fig. 3A). The negative correlation between AXL and immune infiltration was further validated using multiplex immunofluorescence staining of AXL, CD8α, and CD103 in TKI-treated murine tumor tissues. TKI-treated tumors showed an increased AXL expression, concomitant with a decreased infiltration of CD8^+^ and CD103^+^ immune cells (Fig. 2C and Suppl. Fig. 3B). Applying Tumor Immune Dysfunction and Exclusion (TIDE) analysis [25] in TCGA-LIHC dataset, HCC patients were segregated into immunotherapy non-responders and immunotherapy responders according to their TIDE scores. Notably, patients who are defined as immunotherapy non-responders had a higher AXL expression (Fig. 2D, E). In addition, HCC patients with high AXL expression showed a higher T-cell dysfunction score and T-cell exclusion score (Fig. 2F, G). In PD-L1-low-expressing patients, AXL expression could also predict survival as high AXL expression was correlated with shorter overall survival time (Suppl. Fig. 3C). These results suggest high AXL expression to be associated with reduced immune infiltration, and AXL may predict sorafenib and immunotherapy response.

### AXL inhibition induces TNF-α expression and promotes STING-type I interferon pathway

**Fig. 3:**
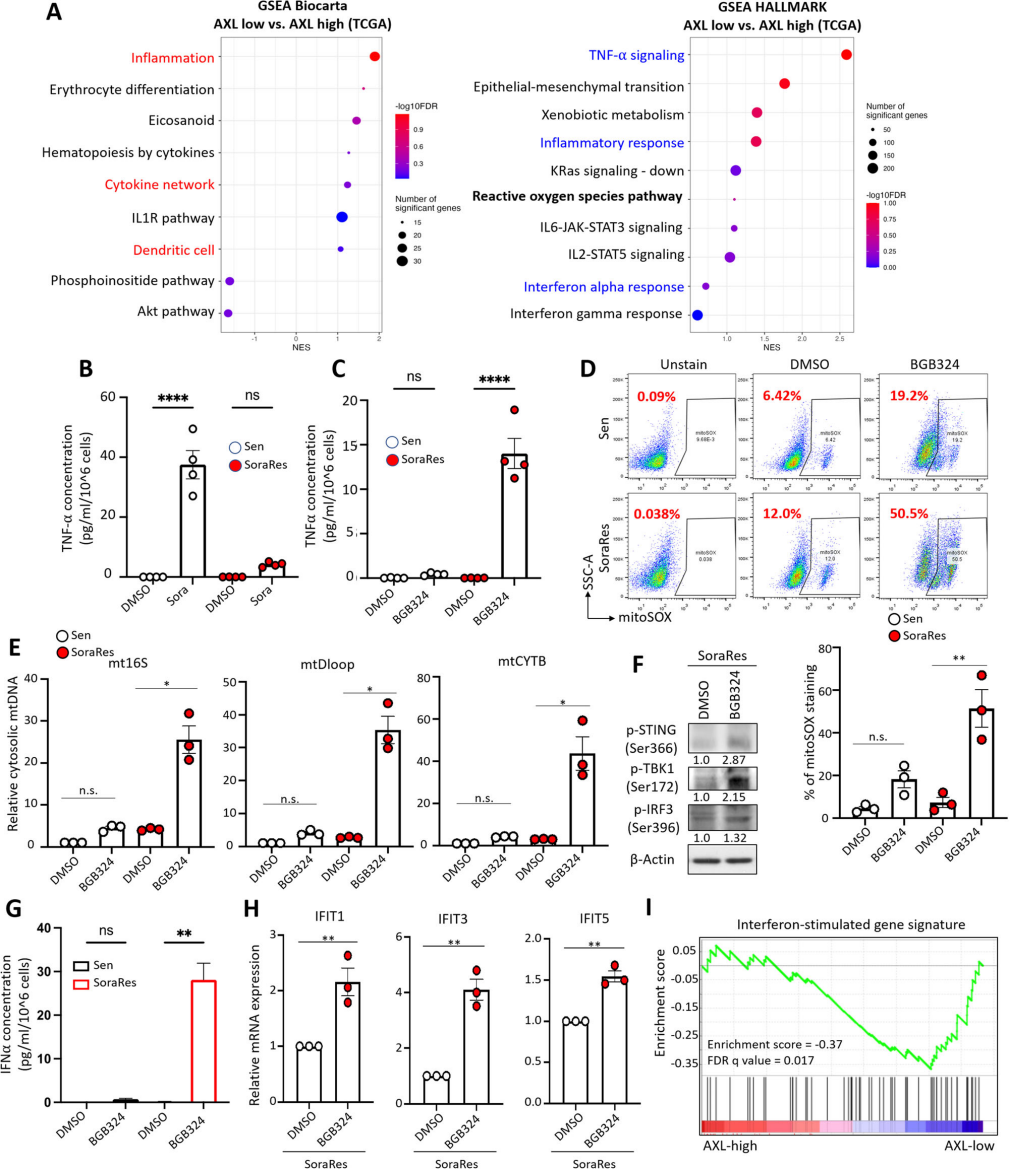
AXL inhibition induces TNF-α expression and promotes STING-type I interferon pathway. **A** Gene Set Enrichment Analysis (GSEA) of transcriptomes of AXL-low versus AXL-high HCC patients from TCGA-LIHC; left - Biocarta and right - Hallmark. **B** ELISA quantification of secretory TNF-α in sorafenib-sensitive (Sen) and sorafenib-resistant (SoraRes) HepG2 cells upon sorafenib treatment. **C** ELISA quantification of secretory TNF-α in Sen and SoraRes HepG2 cells upon BGB treatment. **D** Representative FACS plots (top) and percentages (bottom-right chart) of mitoSOX staining in Sen and SoraRes HepG2 cells upon BGB324 treatment. **E** qRT-PCR quantification of mt16S, mtDloop, and mtCYTB levels in the cytosol extract of Sen and SoraRes HepG2 cells upon BGB324 treatment. **F** WB analysis of STING pathway in SoraRes HepG2 cells upon BGB324 treatment. **G** ELISA quantification of secretory IFN-α in Sen and SoraRes HepG2 cells treated with BGB324. **H** qRT-PCR quantification of ISGs in Sen and SoraRes HepG2 cells treated with BGB324. **I** GSEA of AXL-high versus AXL-low HCC patients from TCGA-LIHC showing a negative correlation with interferon-stimulated gene signature. *p < 0.05; **p < 0.01; ****p < 0.0001; n.s. not significant on a two-tailed unpaired Student’s *t* test or one-way ANOVA with Bonferroni’s multiple comparisons test.

To elucidate the molecular mechanisms underlying AXL-driven immunosuppressive phenotypes in TKI-resistant HCC, TCGA-LIHC dataset was divided into AXL-low and AXL-high groups and subjected to pathway enrichment analyses. GSEA results similarly showed a negative association of signatures involving inflammation, cytokine network, and dendritic cells in AXL-high patients (Fig. 3A), in accordance with the enriched signatures observed in TKIRes samples (Fig. 1A). Enriched HALLMARK pathways related to inflammatory responses including TNF-α signaling and interferon alpha response were commonly observed in AXL-low patients and Sen samples (Fig. 3A), suggesting an overlapping phenotype between AXL-high and TKI-resistant HCC and a potential contributing role of AXL in the immunosuppressive phenotypes of TKI-resistant HCC.

In particular, TNF-α signaling ranks at the top among the enriched pathways in both Sen versus TKIRes samples and AXL-low versus AXL-high patients (Figs. 1A and 3A). Intracellular expression and secretion of TNF-α expression were highly increased in Sen cells, but were not induced in SoraRes HepG2 cells upon sorafenib treatment (Fig. 3B and Suppl. Fig. 4A). Conversely, blockade of AXL kinase activity using AXL selective inhibitor BGB324 resulted in a more prominent increase of TNF-α expression and secretion in SoraRes cells (Fig. 3C and Suppl. Fig. 4B). TNF-α has been known for its role in promoting reactive oxygen species (ROS) and oxidative stress, which induces cell death [26, 27]. In accordance with TNF-α levels, sorafenib treatment greatly enhanced mitochondrial stress in Sen cells but not in SoraRes cells (Suppl. Fig. 4C). High accumulated levels of mitochondrial ROS might cause prominent damage to the mitochondrial, leading to the release of mitochondrial DNA to the cytoplasm of cells [28]. We also examined the abundance of cytosolic mitochondrial DNA (mtDNA) in SoraRes and Sen cells treated with a high concentration of sorafenib. Consistent with augmented mitochondrial ROS level, cytosolic mtDNA was drastically induced in Sen cells but was maintained at a similar level in SoraRes cells after sorafenib treatment, despite SoraRes cells having higher basal cytosolic mtDNA abundance than Sen cells (Suppl. Fig. 4D).

The presence of cytosolic DNA could trigger the nucleotide-sensing mechanism and activate the cGAS-STING-type I interferon (IFN-I) pathway [29]. The expression of p-STING, p-TBK1, and p-IRF3 were lower in SoraRes cells under sorafenib treatment (Suppl Fig. 4E). Activation of STING pathway was accompanied by an increased expression and secretion of interferon-alpha (IFN-α) in Sen cells upon sorafenib treatment (Suppl. Fig. 4F, G). The production and signaling of IFN-I lead to the induction of IFN-stimulated genes (ISGs) [30]. TKIRes samples were negatively correlated with interferon-stimulated gene signature (Suppl. Fig. 4H). ISGs, including IFIT1, IFIT3, and IFIT5 were validated to be highly expressed in Sen cells when compared with SoraRes cells (Suppl. Fig. 4I). Similar results were observed in Sen and SoraRes cells derived from PLC/PRF/5 cell line (Suppl. Fig. 5A–D).

In contrary, pharmacological inhibition of AXL by BGB324 could rescue the suppressed ROS level in SoraRes cells and promote cytosolic mtDNA release in SoraRes cells but not in Sen cells (Fig. 3D, E). STING pathways, IFN-α and ISGs were highly upregulated upon AXL inhibition (Fig. 3F–H and Suppl. Fig. 4J). Consistent results were observed with AXL silencing in two lines of SoraRes cells (Suppl. Fig. 6A–D and Suppl. Fig. 7A–D). Correspondingly, AXL-high patients were negatively correlated with interferon-stimulated gene signature (Fig. 3I). Taken together, these results suggest that AXL suppresses TNF-α and STING-IFN-I pathways in sorafenib-resistant HCC.

### Knockdown of PDPK1 phenocopies AXL inhibition in sorafenib-resistant HCC

**Fig. 4:**
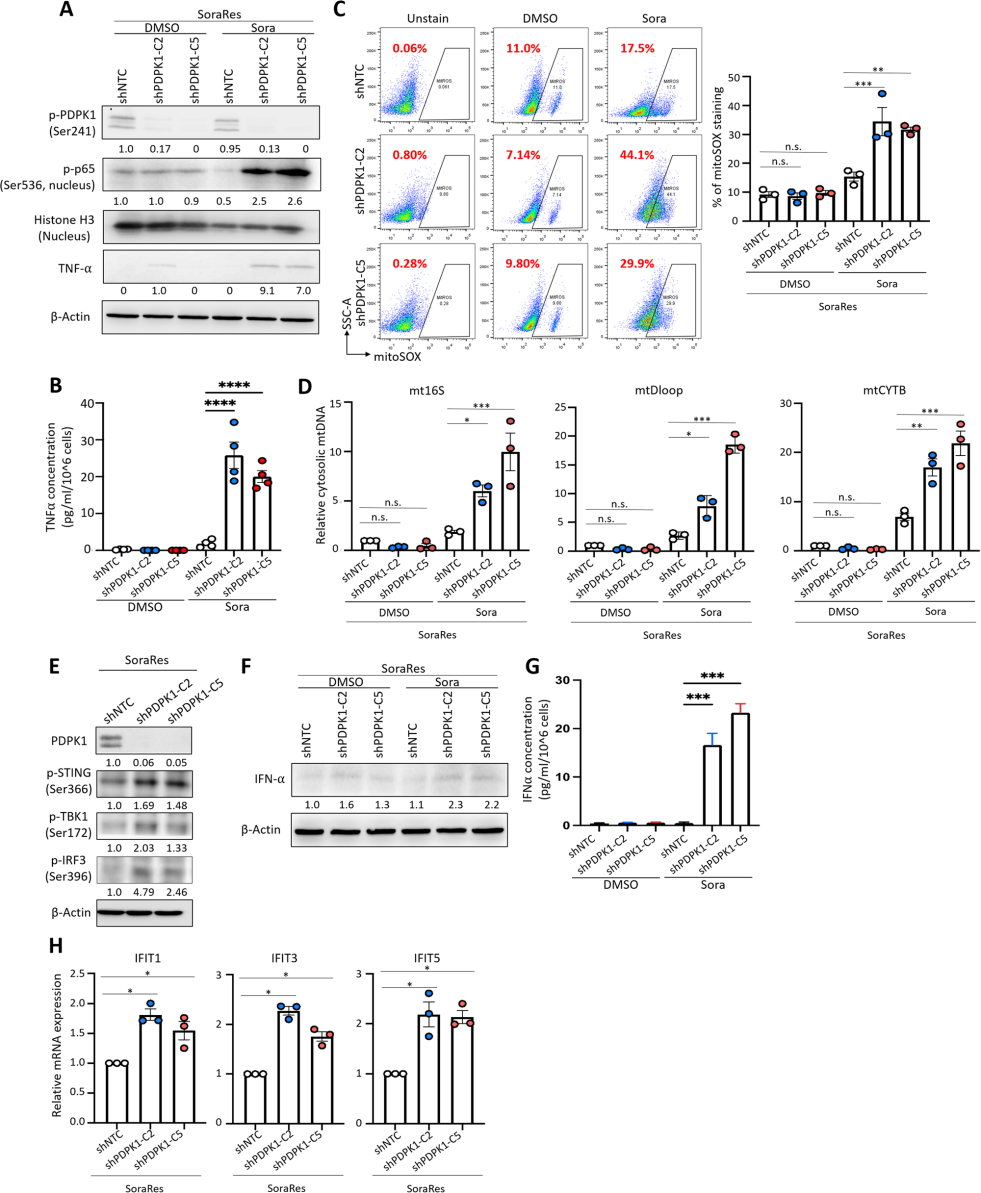
Knockdown of PDPK1 phenocopies AXL inhibition in sorafenib-resistant HCC. **A** WB analysis of p-PDPK1, nuclear p-p65, nuclear histone H3, and TNF-α in SoraRes HepG2 cells with PDPK1 knockdown upon sorafenib treatment. **B** ELISA quantification of secretory TNF-α in SoraRes HepG2 cells with PDPK1 knockdown upon sorafenib treatment. **C** Representative FACS plots (left) and percentages (right chart) of mitoSOX staining in SoraRes HepG2 cells with non-target control (shNTC) or PDPK1 knockdown (shPDPK1-C2 and shPDPK1-C5). **D** qRT-PCR quantification of mt16S, mtDloop, and mtCYTB levels in the cytosol extract of SoraRes HepG2 cells with PDPK1 knockdown upon sorafenib treatment. **E** WB analysis of STING pathway in SoraRes HepG2 cells with PDPK1 knockdown. **F** WB analysis of IFN-α in SoraRes HepG2 cells with PDPK1 knockdown upon sorafenib treatment. **G** ELISA quantification of secretory IFN-α in SoraRes HepG2 cells with PDPK1 knockdown upon sorafenib treatment. **H** qRT-PCR quantification of mt16S, mtDloop, and mtCYTB levels in the cytosol extract of SoraRes HepG2 cells with PDPK1 knockdown. *p < 0.05; **p < 0.01; ***p < 0.001; ****p < 0.0001; n.s. not significant on one-way ANOVA with Bonferroni’s multiple comparisons test.

AXL, as a receptor tyrosine kinase activates a number of downstream signaling pathways, including PI3K/PDPK1, MAPK, and mTOR pathways to promote tumorigenesis and enhance cell survival [8]. To determine the underlying mechanism that AXL regulates TNF-α and STING-IFN-I signaling, we examined the proteomic expression of key players of AXL-regulated signaling pathways. Phosphorylated PDPK1 was the only target with concordant upregulation as AXL in SoraRes cells (Suppl. Fig. 8A). AXL inhibition with BGB324 and AXL knockdown consistently reduced the expression of p-PDPK1 in SoraRes cells (Suppl. Fig. 8B, C). PDPK1 activates various downstream kinase signaling, including MAPK and AKT/mTOR pathways [31]. However, we did not observe consistent changes of MAPK and AKT/mTOR pathways in our systems (Suppl. Fig. 8A–C). A previous study has showed that PDPK1 activates NF-κB pathway by directly phosphorylating IκB kinase β (IKKβ), leading to the nuclear translocation of NF-κB and subsequent activation of anti-apoptotic gene expression [32]. Multiple studies have indicated that NF-κB is one of the major transcription factors with putative binding site on TNF-α promoter [33]. In addition, in GSEA analysis comparing Sen and TKIRes HCC samples, we also found TNF-α signaling via NF-κB to be the top-ranked down-regulated Hallmark (Suppl. Fig. 9A), further suggesting AXL/PDPK1 axis to regulate downstream TNF-α expression through NF-κB pathway. Indeed, Western blot results show that phospho-IKKα/β level was increased in Sen cells upon sorafenib treatment, as well as in SoraRes cells upon AXL inhibition by BGB324 (Suppl. Fig. 9B, C). PDPK1 knockdown also resulted in an increased phospho-IKKα/β level under sorafenib treatment (Suppl. Fig. 9D). Consistently, nuclear phospho-p65 levels were increased in Sen cells upon sorafenib treatment, in SoraRes cells upon AXL inhibition, and in SoraRes cells upon PDPK1 knockdown (Fig. 4A, Suppl. Fig. 9B, C). These results suggest a potential regulatory role of AXL/PDPK1/NF-kB axis in sorafenib-resistant HCC.

We further confirmed that knockdown of PDPK1 similarly promoted TNF-α expression and secretion (Fig. 4A, B), suggesting that suppression of AXL could promote TNF-α expression through PDPK1/NF-kB-dependent pathway. Knockdown of PDPK1 similarly promoted mitochondrial damage under high concentrations of sorafenib treatment (Fig. 4C, D). STING pathway was activated upon PDPK1 knockdown in SoraRes cells (Fig. 4E). IFN-α and ISGs were highly upregulated upon PDPK1 knockdown (Fig. 4F–H). Similar findings were obtained using SoraRes cells established from PLC/PRF/5 cells (Suppl. Fig. 10A–D). These results suggest that AXL signals through PDPK1 to suppress TNF-α and STING-IFN-I pathways in sorafenib-resistant HCC.

Three potential mechanisms have been suggested to govern the release of mtDNA to the cytosol in stressed cells, including the reduced level of TFAM to indicate defective mitochondrial integrity, BAX/BAK-dependent permeabilization of outer mitochondrial membrane, and the opening of mitochondrial permeability transition pores (mPTP) [34–36]. We observed localization of BAX and BAK to the mitochondria upon sorafenib treatment in Sen cells and upon BGB324 treatment in SoraRes cells, and reduced cytosolic mtDNA detection through inhibition of mPTP with cyclosporin A (CsA) (Suppl. Fig. 11A–F). However, TFAM may not be involved in regulating the release of mtDNA in our system (Suppl. Fig. 11G–I). To confirm if cytosolic mtDNA is crucial for the activation of STING pathway under AXL inhibition, we treated SoraRes cells with mitoTEMPO, which alleviates mitochondrial ROS [37] and ethidium bromide, which inhibits mtDNA replication and transcription and depletes mtDNA [38]. With the alleviation of mitochondrial ROS and reduced cytosolic mtDNA level (Suppl. Fig. 12A, C), activation of STING pathway could be rescued in SoraRes cells treated with BGB324 (Suppl. Fig. 12B, D).

### AXL inhibits sorafenib-induced immunogenic cell death, which could be rescued by TNF-α and IFN-α in sorafenib-resistant HCC

The release of mtDNA into the cytosol from the damaged mitochondria may instigate the intracellular danger signaling pathways such as STING-type I IFN response that govern immunogenic cell death (ICD) in dying cancer cells [39]. Stressed cells undergoing ICD release immune-stimulatory molecules known as danger-associated molecular patterns (DAMPs) which are further presented to dendritic cells for T-cell priming [39]. TKIs were reported to exert immunomodulatory effects by affecting immune cell infiltration or functionality [40]. However, little evidence has depicted how TKIs might alter the immunogenicity of cancer cells. Given the enhanced oxidative burst and mtDNA release from mitochondria triggered by sorafenib treatment in Sen cells, we went on to investigate if sorafenib may induce ICD and trigger the release of DAMPs, which confer adjuvanticity to support adaptive immunity in cancer treatment [41].

**Fig. 5:**
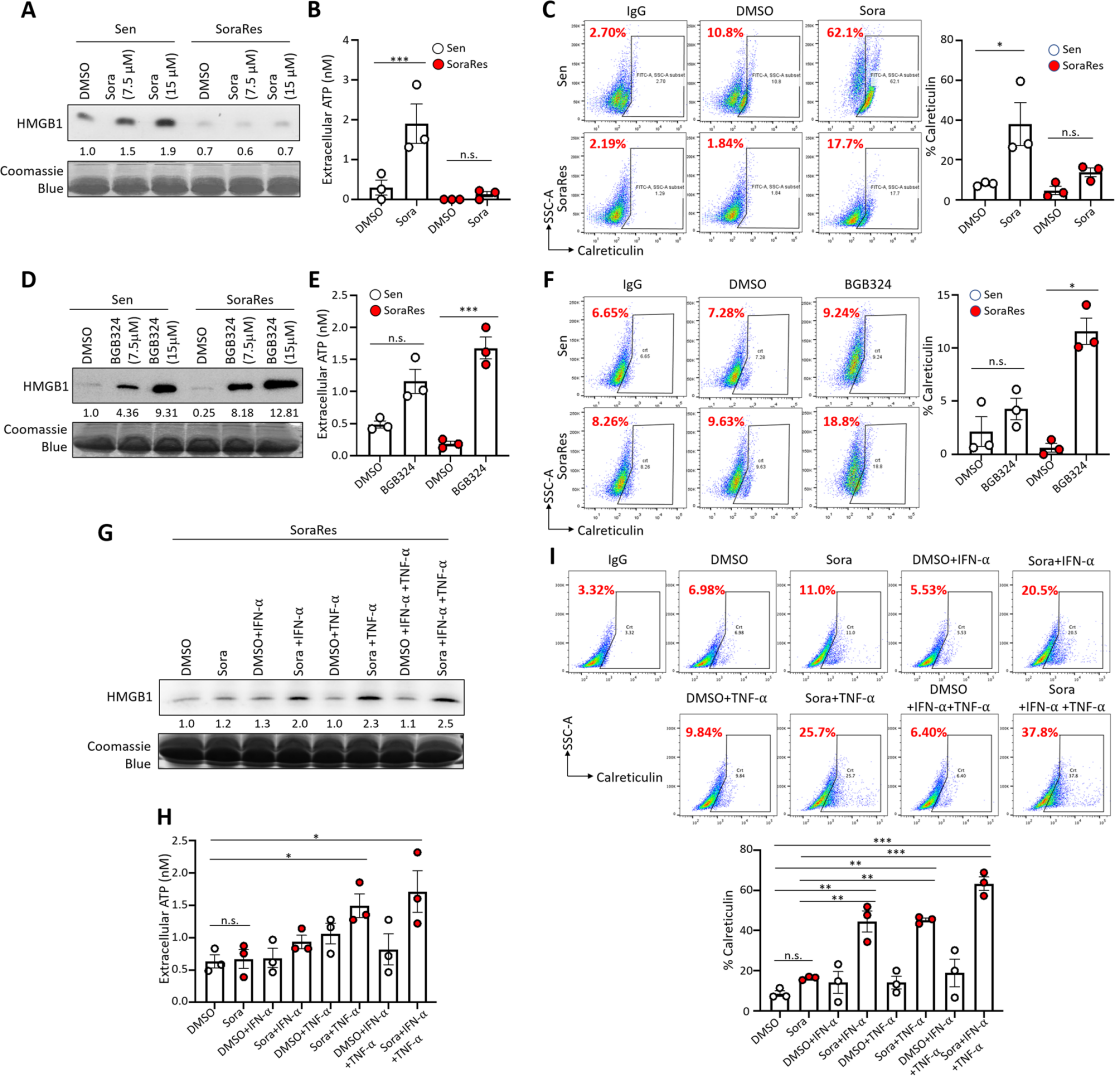
AXL inhibits sorafenib-induced immunogenic cell death, which could be rescued by TNF-α and IFN-α in sorafenib-resistant HCC. **A** WB analysis of secretory HMGB1 in the conditioned media of Sen and SoraRes HepG2 cells upon sorafenib treatment at the indicated concentrations. **B** ATP concentrations in the conditioned media collected from Sen and SoraRes HepG2 cells upon sorafenib treatment. **C** Representative FACS plots (left) and percentages (right chart) of membrane calreticulin expression in Sen and SoraRes HepG2 cells upon sorafenib treatment. **D** WB analysis of secretory HMGB1 in the conditioned media of Sen and SoraRes HepG2 cells treated with BGB324 at the indicated concentrations. **E** ATP concentrations in the conditioned media collected from Sen and SoraRes HepG2 cells treated with BGB324. **F** Representative FACS plots (left) and percentages (right chart) of membrane calreticulin expression in Sen and SoraRes HepG2 cells treated with BGB324. **G** WB analysis of secretory HMGB1 in the conditioned media of SoraRes HepG2 cells treated with either IFN-α or TNF-α alone or combined IFN-α and TNF-α in the presence or absence of sorafenib. **H** ATP concentrations in the conditioned media collected from SoraRes HepG2 cells treated with either IFN-α or TNF-α alone or combined IFN-α and TNF-α in the presence or absence of sorafenib. **I** Representative FACS plots (top) and percentages (bottom chart) of membrane calreticulin expression in SoraRes HepG2 cells treated with either IFN-α or TNF-α alone or combined IFN-α and TNF-α in the presence or absence of sorafenib. *p < 0.05; **p < 0.01; ***p < 0.001; n.s. not significant on a two-tailed unpaired Student’s *t* test for (**B**–**F**) and one-way ANOVA with Bonferroni’s multiple comparisons test for **H**, **I**.

We found that sorafenib treatment triggered ICD in Sen cells as evident by increased DAMPs, including secretory HMGB1, extracellular ATP, and membrane translocation of calreticulin [41] (Fig. 5A–C and Suppl. Fig. 13A–C). However, DAMPs were not induced in SoraRes cells after sorafenib treatment (Fig. 5A–C and Suppl. Fig. 13A–C). In mice receiving TKI treatment, residual tumors from sorafenib or lenvatinib treatment showed elevated expression of AXL and p-PDPK1, whereas HMGB1 and calreticulin levels were lower compared with the control tumors (Suppl. Fig. 13D, E). Blockade of AXL with BGB324 could elicit ICD response in both Sen and SoraRes cells, but to a greater extent in SoraRes cells (Fig. 5D–F). Similar findings were observed in AXL- and PDPK1-depleted SoraRes cells which showed prominent ICD response after sorafenib treatment (Suppl. Fig. 14A–F). We next sought to investigate the AXL-dependent factors that trigger ICD. Treatment of TNF-α or IFN-α alone could induce ICD in SoraRes cells (Fig. 5G–I). Combination of TNF-α and IFN-α, however did not further enhance ICD response (Fig. 5G–I). These results suggest that sorafenib-resistant HCC cells not succumbing to ICD could be rescued through inhibiting AXL-dependent TNF-α and IFN-α suppression.

### Co-treatment of lenvatinib and AXL exerts therapeutic effects in preclinical HCC models

In view of the functional roles of AXL in modulating the TKI resistance in HCC, we sought to examine the therapeutic effects of blocking AXL alone and in combination with sorafenib and lenvatinib in preclinical HCC models. Treatment of BGB324 alone in treatment-naïve HCC patient-derived 3D organoids resulted in moderate growth inhibition, whereas the combination of BGB324 and sorafenib or lenvatinib would result in a more drastic inhibitory effect (Suppl. Fig. 15A–D).

**Fig. 6:**
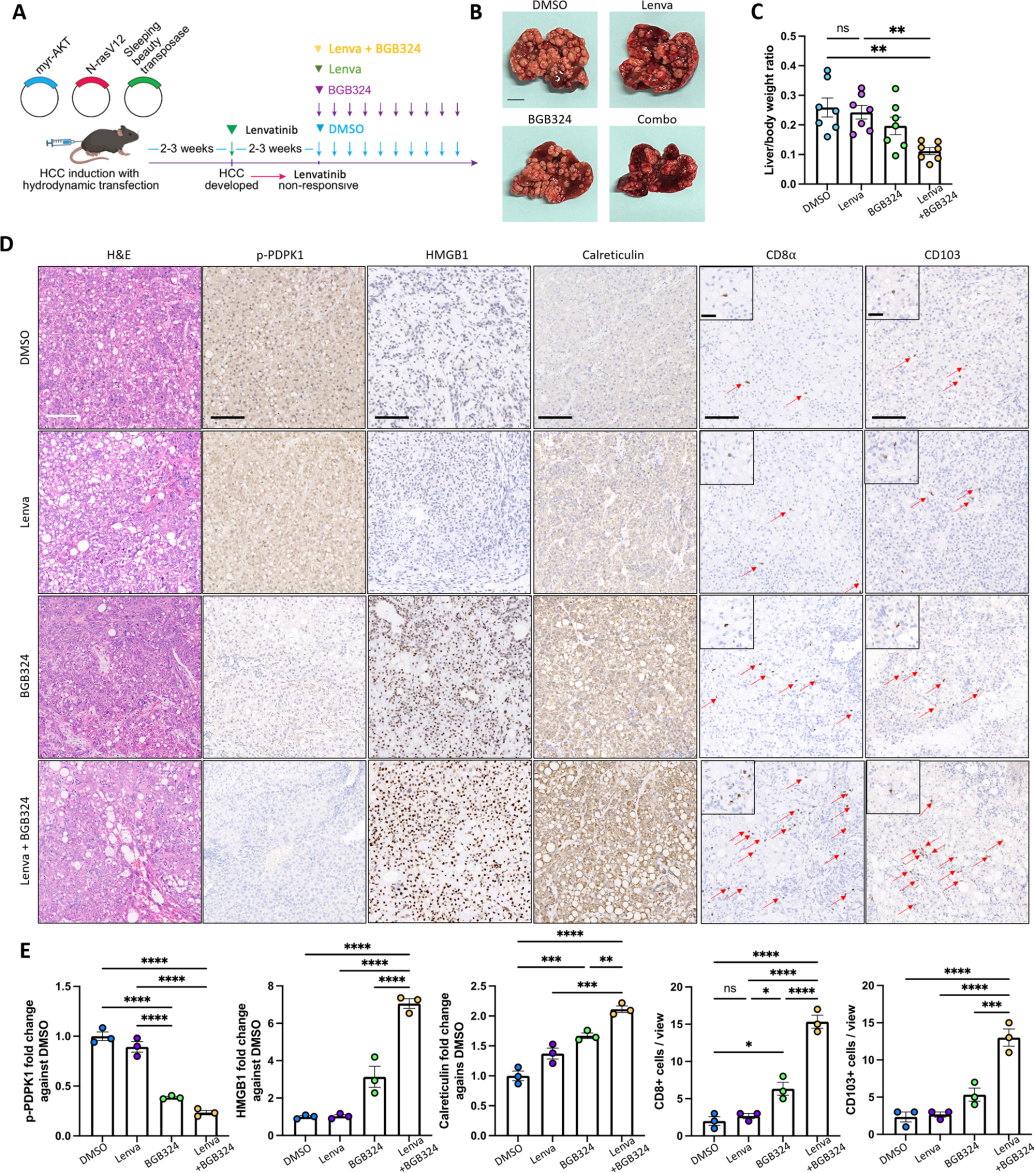
Co-treatment of lenvatinib and AXL exerts therapeutic effects in preclinical HCC mouse model. **A** Schematic diagram illustrating the treatment scheme of either BGB324 or lenvatinib alone, or in combination with lenvatinib and BGB324 in lenvatinib non-responsive spontaneous liver tumors established by hydrodynamic tail vein injection of oncogenic plasmids. **B** Representative images of livers resected from mice receiving either BGB324 or lenvatinib alone, or the combination treatment. Scale bar = 1 cm. **C** Liver to body weight ratio of mice after treatment (*n* = 8 per group). Data representative of one experiment. **D** Representative H&E and IHC images showing p-PDPK1, HMGB1, calreticulin, CD8α, and CD103 expression in the tumor sections after single or combination treatment. Scale bar = 100 µm and 50 µm (inset). Red arrows indicating positive signals of CD8α and CD103. **E** Bar charts showing the quantification of p-PDPK1, HMGB1, calreticulin staining intensities, and CD8α^+^ and CD103^+^ cells in three independent fields. **p < 0.01 on a Cox–Mantel log-rank test in the survival curve. *p < 0.05; **p < 0.01; ***p < 0.001; ****p < 0.0001; n.s. not significant on one-way ANOVA with Bonferroni’s multiple comparisons test.

To extend our findings to an in vivo setting, we applied a previously established TKI-resistant model using immune-competent C57BL/6 N mice, in which TKI-resistant HCC tumors were developed from hydrodynamic tail vein injection of oncogenic plasmids expressing N-Ras and Akt and subsequent continuous treatment with sorafenib or lenvatinib (Fig. 6A and Suppl. Fig. 16A) [15]. Higher expression of AXL in sorafenib non-responsive tumor was confirmed by IHC analysis (Suppl. Fig. 16B). We sought to investigate if BGB324 treatment could suppress tumorigenesis and progression in these two TKI-resistant mouse models. Treatment of BGB324 alone resulted in significant suppression of tumor growth and extension of mice survival in sorafenib non-responsive tumors but resulted in a marginal reduction in lenvatinib non-responsive tumors (Fig. 6B–E and Suppl. Fig. 16C, D). However, combination treatment of BGB324 and lenvatinib showed a significant growth inhibition compared with a single treatment of BGB324 or lenvatinib (Fig. 6B, C). AXL inhibition alone by BGB324 or in combination with lenvatinib resulted in PDPK1 suppression and ICD induction, as evident by increased HMGB1 and calreticulin staining (Fig. 6D, E, and Suppl. Fig. 16G, H). Increased infiltration of activated CD8^+^ T cells and CD103^+^ dendritic cells was also observed in sorafenib non-responsive tumors treated with BGB324 alone (Suppl. Fig. 16F–H) or in lenvatinib non-responsive tumors treated with BGB324 and lenvatinib (Fig. 6D, E).

### TKI treatment compromises immunotherapy efficacy which could be rescued by AXL inhibition

As we observed an altered immune landscape resulted from TKI treatment (Fig. 1D), we wondered if this could cause immune evasion and compromise anti-PD-1 efficacy as a second-line treatment in HCC. We transplanted the residual tumors after sorafenib or DMSO treatment to secondary recipient mice and treated the mice with either anti-PD-1 or IgG antibody (Suppl. Fig. 17A). Mice bearing primary tumors treated with sorafenib were not responsive to anti-PD-1 treatment and showed no difference in tumor volume compared with IgG control (Suppl. Fig. 17B, C). The littermate-bearing tumors with prior DMSO treatment showed drastic suppression of tumor growth under anti-PD-1 treatment (Suppl. Fig. 17B, C). Residual tumors receiving prior sorafenib treatment showed a stronger expression of AXL and p-PDPK1 compared with tumors receiving prior DMSO treatment (Suppl. Fig. 17D, E). Anti-PD-1 treatment would not alter AXL and p-PDPK1 expression (Suppl. Fig. 17D, E). However, tumors without prior sorafenib treatment were not only responsive to anti-PD-1 treatment, but also showed a significant increase in the numbers of infiltrating CD8^+^ and CD103^+^ cells (Suppl. Fig. 17D, E). Regardless of the secondary treatment received, the numbers of infiltrating CD8^+^ and CD103^+^ cells in the tumors with prior sorafenib treatment were lower than in tumors with DMSO and anti-PD-1 treatment (Suppl. Fig. 17D, E). These data suggest that TKI treatment might compromise immunotherapy efficacy in HCC patients.

**Fig. 7:**
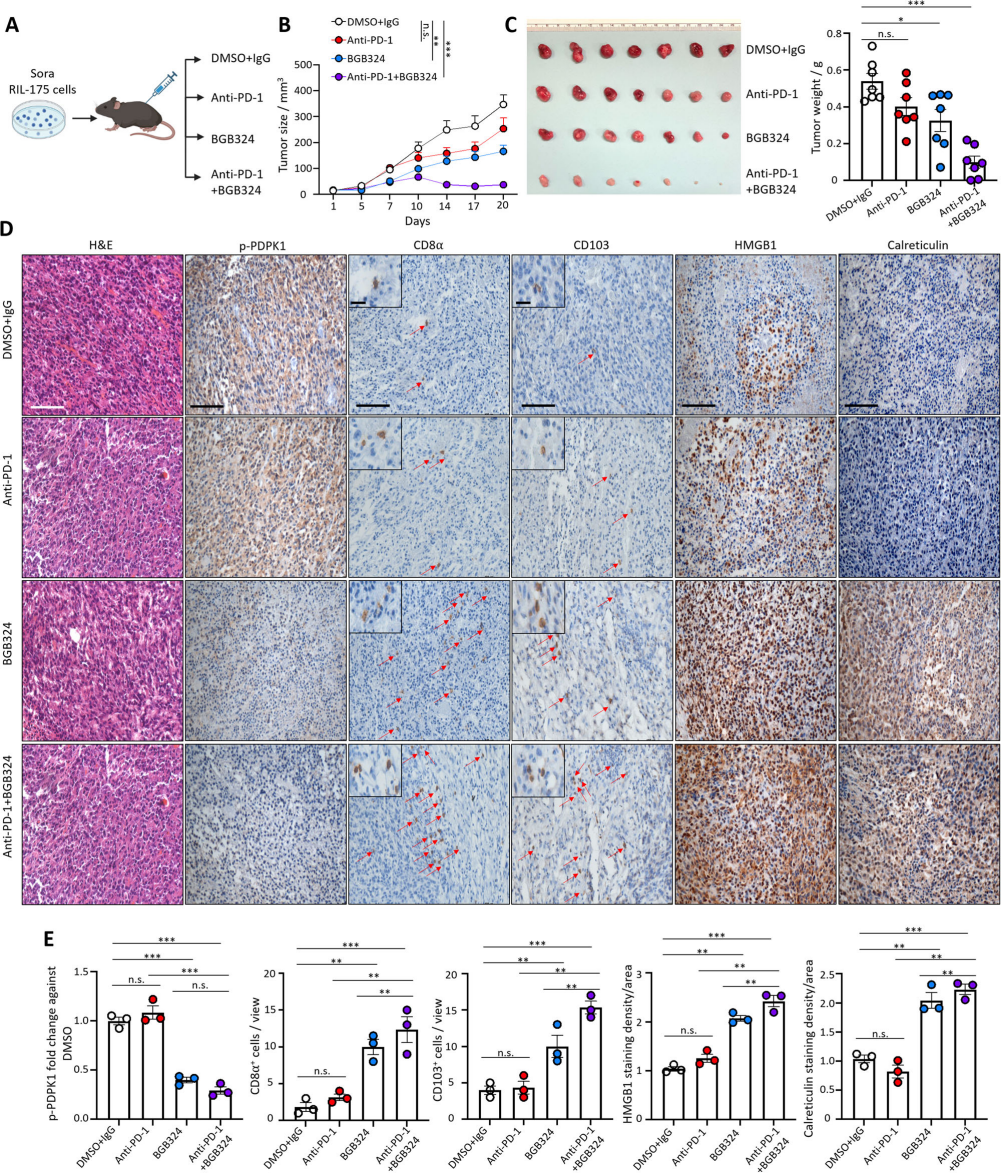
AXL inhibition sensitizes TKI-resistant HCC tumors towards immunotherapy. **A** Schematic diagram illustrating single and combined anti-PD-1 and BGB324 treatment in secondary xenografts established from residual RIL-175 HCC cells after sorafenib treatment. **B** Tumor growth curves across the treatment course (*n* = 7 per group). Data representative of one experiment. **C** Representative image of resected residual tumor nodules. **D** Bar chart showing the tumor weight of resected xenografts after treatment. **E** Representative H&E and IHC images showing p-PDPK1, HMGB1, calreticulin, CD8α, and CD103 expression in the tumor sections after single anti-PD-1 antibody, single BGB324, combined treatment, and respective controls. Scale bar = 100 µm and 25 µm (inset). Red arrows indicate positive signals of CD8α and CD103. **E** Bar charts showing the quantification of p-PDPK1, HMGB1, calreticulin staining intensities, and CD8α^+^ and CD103^+^ cells in three independent fields. *p < 0.05; **p < 0.01; ***p < 0.001; n.s. not significant on one-way ANOVA with Bonferroni’s multiple comparisons test.

We further investigated the therapeutic effect of AXL inhibition in overcoming the compromised immunotherapy efficacy following TKI treatment. Mice bearing tumors with prior sorafenib treatment were treated with either anti-PD-1 or BGB324 treatment alone, or the combination of anti-PD-1 and BGB324 (Fig. 7A). The combined treatment of anti-PD-1 and BGB324 showed the strongest tumor suppressive effect compared with single treatment or control treatment (Fig. 7B, C). This result suggested that BGB324 treatment was able to suppress tumor growth and potentiated the anti-PD-1 treatment response. BGB324 treatment could suppress PDPK1 activation and promote the infiltration of CD8+ and CD103+ cells into the tumors (Fig. 7D, E). ICD markers HMGB1 and calreticulin were also increased after BGB324 treatment (Fig. 7D, E). The effects were more prominent in the combined treatment compared with the single BGB324 treatment (Fig. 7D, E). The therapeutic efficacy of the combination treatment of anti-PD-1 and BGB324 was found to be STING-dependent, as evident by the loss of anti-tumor effect and the reduced immunostimulatory effect upon STING inhibition by its inhibitor H-151 in the combination treatment group (Suppl. Fig. 18A–E). Taken together, the results suggest that AXL inhibition could overcome the immunosuppressive effect exerted by TKI treatment and re-sensitize the tumors towards immunotherapy treatment through STING activation.

## Discussion

In this study, we showed that prior TKI treatment renders HCC insensitive to subsequent immunotherapy, at least in part, through the impairment of TNF-α and STING-IFN-α signaling and immunogenic cell death, which may lead to reduced intratumoral infiltration of CD8^+^ T cells and cDC1 dendritic cells. Mechanistically, upregulation of AXL and its downstream signaling mediator PDPK1 suppressed the production of proinflammatory factors TNF-α and IFN-α, and further attenuated the activation of ICD and the release of DAMPs. The reduction of these critical factors could contribute to compromised anti-tumor immunity in TKI-resistant HCC, which showed reduced infiltrating cDC1 and CD8^+^ T cells. Pharmacological targeting of AXL in combination with TKI could evoke a more immunogenic phenotype of the tumor and potentiate the treatment efficacy of subsequent immunotherapy in TKI-resistant HCC. Our findings provide novel mechanistic evidence of AXL-suppressed TNF-α/IFN-α signaling and a proof-of-concept with preclinical model to support the clinical testing of AXL inhibition in combination with immunotherapy for HCC patients who progress on first-line TKI treatment.

TKI treatment has been shown to elicit immunomodulatory effects in the host, as demonstrated by varied infiltration frequency and perturbed function of circulating and tumor-infiltrating immune cells post-therapy in preclinical models and patient samples. In an earlier study, sorafenib was found to affect neither the induction of antigen-specific T cells nor the number of Treg cells in PBMCs [42]. The immunoenhancing effects of sorafenib was later observed in different studies where reduced abundance of immunosuppressive Treg cells and MDSCs, and augmented function and migration of CD8^+^ T cells supported an immune-permissive tumor microenvironment which might potentiate adoptive T-cell therapy [43, 44]. A contrasting immunosuppressive effect of sorafenib was also observed in various studies. Sorafenib was shown to inhibit T-cell proliferation [45], which might be caused by suppressive dendritic cells or reduced plasmacytoid dendritic cells [42, 46, 47]. On the other hand, lenvatinib has been shown to mount an anti-tumor immunity response. Clinically, short duration of lenvatinib treatment could improve patients’ immune status with reduced immunosuppressive immune cells and increased cytotoxic T lymphocytes [48]. Lenvatinib treatment decreased the proportion of monocytes and macrophages but increased neutrophil recruitment and increased CD8^+^ T cells in HCC mouse models [49–51]. Despite the observations of variable immunomodulatory effects of TKIs in HCC TME, direct modulation of cancer cells, which may provoke immunogenic response to alter the immune landscape in the TME by TKIs is not yet confirmed. In a recent study, lenvatinib treatment was shown to induce ICD in HCC cells [52]. Our study provided an additional insight into underlying molecular mechanism of adaptation and tolerance of HCC in response to ICD induction under prolonged treatment of TKI. Our findings suggest that TKI resistance and tolerance render HCC not succumbing to TKI-induced ICD, resulting in an immunologically cold TME supported by reduced proinflammatory factors and DAMPs.

The concept of cross-resistance between molecular targeted therapy and immunotherapy was first recognized in melanoma [2], in which targeted therapy with MAPK inhibitor creates an immunosuppressive TME to resist the subsequent immunotherapy. In non-small cell lung cancer, results from clinical trials have revealed that there were no survival benefits for EGFR-mutant non-small cell lung cancer (NSCLC) patients with the treatment of subsequent immunotherapy [53, 54]. The underlying mechanism of poor response to second-line immunotherapy was shown to be attributable to an EMT-associated immunologically cold phenotype in TKI-resistant NSCLC tumors [3]. AXL has been implicated to correlate with lower response rates of anti-PD-1 blockade and worse overall survival in clear-cell renal carcinoma (ccRCC) patients who were refractory to VEGF-directed therapy [11]. In line with these studies, our findings supported the notion of cross-resistance in TKI-resistant HCC. We provided a distinct and HCC-specific mechanism of AXL-driven tumor-intrinsic signaling to promote an immunosuppressive phenotype of TKI-resistant HCC and further proposed the application of AXL targeting to overcome a potential cross-resistance in HCC.

In light of the oncogenic functions of AXL, clinical trials targeting AXL with different means, such as small molecular inhibitors, antibody-drug conjugates, and monoclonal antibodies have been conducted to evaluate the therapeutic potential of AXL inhibition for anti-cancer treatment [55]. Furthermore, research findings revealing the immunomodulatory functions of AXL have provided strong support for the combinational treatment of AXL inhibition and ICI treatment in primary tumor models of breast and lung cancers [9, 56, 57]. These preclinical data also provided the basis for the clinical trials with AXL inhibition and ICI-based therapy in cancer patients [55]. A study in erlotinib-resistant NSCLC indicates that AXL signaling supports autophagy-dependent drug-resistant persister cell phenotype, and targeting AXL could elicit ICD [56]. Clinically, high AXL expression was correlated with lower anti-PD-1 treatment response in drug-resistant ccRCC patients [11]. Although AXL has been implicated in conferring drug resistance and mediating immunomodulatory functions, the therapeutic potential of AXL inhibition in potentiating ICI-based therapy in drug-resistant cancers, for example, HCC, has not been studied. Here, our data collectively describe an underlying mechanism and offer additional proof-of-concept to demonstrate that targeting AXL could be a therapeutic opportunity to bolster immunotherapy in HCC patients who are refractory to first-line TKI treatment.

Combination therapy harnessing the improved vascular perfusion and immunomodulation effects of anti-angiogenic TKI and ICI has been a research hotspot. Preclinical data showed that a combination of lenvatinib and anti-PD-1 treatment resulted in increased dendritic cell infiltrates and induced an immune-active microenvironment with suppression of TGFβ immunosuppressive signaling [58]. Cabozantinib, as a second-line TKI for advanced HCC, targets pro-angiogenic growth factors VEGFR, MET, and the TAM family of kinases, including AXL [59]. Cabozantinib, in combination with immune checkpoint inhibitors as first-line treatment, has shown promising clinical activity in advanced RCC and HCC, with improved progression-free survival and overall survival [60, 61]. These data suggest that targeting kinases, including AXL could be a potential therapeutic opportunity to improve ICI treatment efficacy. However, high-grade toxicities may be imposed with the combination of anti-angiogenic TKI and ICI-based therapy [62]. This could be attributed to the nature of TKI with a broad spectrum of targets that may impose undesirable immunological reactions when applied with immunotherapy [63]. Previous clinical trials and preclinical studies did not report severe toxicity in the combination treatment of AXL inhibition and ICI [12]. However, the potential toxicity effect of AXL inhibition with ICI-based therapy in TKI-resistant HCC patients still warrants further investigation.

### Supplementary information

Supplementary information

Original Data File

aj-checklist

## Data Availability

The materials included in this study are available from the corresponding authors upon reasonable request.
