# Supplementary material for: Targeting AXL induces tumor-intrinsic immunogenic response in tyrosine kinase inhibitor-resistant liver cancer
Source: Cell Death Dis. 2024 Feb 3;15(2):110. doi: 10.1038/s41419-024-06493-0 (PMC10838288; doi:10.1038/s41419-024-06493-0)
Supplement: Supplementary file 1 — Supplementary information [file 41419_2024_6493_MOESM1_ESM.docx]

**Supplemental Information**

**Targeting AXL induces tumor-intrinsic immunogenic response in tyrosine kinase inhibitor-resistant liver cancer**

Yunong Xie^1,2^, Haofeng Wu^1^, Yimiao He^1^, Linglin Liu^1^, Ianto Bosheng Huang^2^, Lei Zhou^2,3^, Cheuk-Yin Lin^2^, Rainbow Wing-Hei Leung^4^, Jia-Jian Loh^2^, Terence Kin-Wah Lee^4^, Jin Ding^5^, Kwan Man^6^, Stephanie Ma^2,7,8^^, Man Tong^1,2^^

^1^ School of Biomedical Sciences, The Chinese University of Hong Kong, Hong Kong, China

^2^ School of Biomedical Sciences, Li Ka Shing Faculty of Medicine, The University of Hong Kong, Hong Kong, China

^3^ Precision Medicine Institute, The First Affiliated Hospital, Sun Yat-Sen University, Guangzhou, China

^4^ Department of Applied Biology and Chemical Technology, The Hong Kong Polytechnic University, Hong Kong, China

^5^ Eastern Hepatobiliary Surgery Hospital, The International Cooperation Laboratory on Signal Transduction, Shanghai, China

^6^ Department of Surgery, School of Clinical Medicine, Li Ka Shing Faculty of Medicine, The University of Hong Kong, Hong Kong, China

^7^ State Key Laboratory of Liver Research, The University of Hong Kong, Hong Kong, China

^8^ Hong Kong University – Shenzhen Hospital, Shenzhen, China

^ co-corresponding authors

**List of content**

Supplemental Table 1

Supplemental Table 2

Supplemental Figure 1

Supplemental Figure 2

Supplemental Figure 3

Supplemental Figure 4

Supplemental Figure 5

Supplemental Figure 6

Supplemental Figure 7

Supplemental Figure 8

Supplemental Figure 9

Supplemental Figure 10

Supplemental Figure 11

Supplemental Figure 12

Supplemental Figure 13

Supplemental Figure 14

Supplemental Figure 15

Supplemental Figure 16

Supplemental Figure 17

Supplemental Figure 18

Supplemental Figure 19

Supplemental Table 1. The sequences used for shRNA and qPCR primers.

| **shRNA** | | |
| --- | --- | --- |
| Gene | Clone ID | Sequence |
| AXL  (shAXL-B9) | TRCN0000001039 | 5’-CCGGCGAAAGAAGGAGACCCGTTATCTCGAGATAACGGGTCTCCTTCTTTCGTTTTT-3’ |
| AXL  (shAXL-B10) | TRCN0000001040 | 5’-CCGGCGAAATCCTCTATGTCAACATCTCGAGATGTTGACATAGAGGATTTCGTTTTT-3’ |
| PDPK1  (shPDPK1-C2) | TRCN0000006260 | 5’-CCGGCGGATCAGAAACCGACACAATCTCGAGATTGTGTCGGTTTCTGATCCGTTTTT-3’ |
| PDPK1  (shPDPK1-C2) | TRCN0000006263 | 5’-CCGGCCAGGGTGTGATTGAATACAACTCGAGTTGTATTCAATCACACCCTGGTTTTT-3’ |
| Non-target control (NTC) |  | 5’-CCGGCAACAAGATGAAGAGCACAACTCGAGTTGGTGCTCTTCATCTTGTTGTTTTT-3’ |
|  | | |
| **qPCR primers** | | |
| Gene | Sequence | |
| Nuclear Actin (nucActin) | F: 5’-AACAGACTCCCCATCCCAAG-3’  R: 5’-CCAGAGGCGTACAGGGATAG-3’ | |
| Mitochondrial 16S (mt16S) | F: 5’-GCCTTCCCCCGTAAATGATA-3’  R: 5’-TTATGCGATTACCGGGCTCT-3’ | |
| Mitochondrial Dloop (mtDloop) | F: 5’-GTCCCTTGACCACCATCCTC-3’  R: 5’-GTAGCACTCTTGTGCGGGAT-3’ | |
| Mitochondrial CYTB (mtCYTB) | F: 5’-AGACAGTCCCACCCTCACAC-3’  R: 5’-GGTGATTCCTAGGGGGTTGT-3’ | |
| IFIT3 | F: 5’-AAAAGCCCAACAACCCAGAAT-3’  R: 5’-CGTATTGGTTATCAGGACTCAGC-3’ | |
| IFIT1 | F: 5’-CAGCCTAGAGGGCAGAACAG-3’  R: 5’-CACCTCAAATGTGGGCTTTT-3’ | |
| IFIT5 | F: 5’-TAAAAAAGGCCTTGGAGGTG-3’  R: 5’-CCAGGTCTGTGTAGGCAAAT-3’ | |

Supplemental Table 2. Primary antibodies used in this study

| **IHC/IF** | | | |
| --- | --- | --- | --- |
| Antibody | **Source (Cat. No.)** | **Concentration** | |
| Rabbit anti-mouse CD8α | Abcam, ab217344 | 1:500 | |
| Goat anti-moue CD103 | R&D Systems | 1:50 | |
| Rabbit anti-human/mouse AXL | Abcam, ab227871 | 1:500 | |
| Rabbit anti-human PDPK1 | ABclonal, AP0426 | 1:200 | |
| Rabbit anti-human/mouse HMGB1 | Abcam, ab79823 | 1:350 | |
| Rabbit anti-human/mouse Calreticulin | Abcam, ab92516 | 1:250 | |
| Rabbit anti-human Bax | Cell signaling, 5023 | 1:50 | |
| Rabbit anti-human Bak | Cell signaling, 12105 | 1:100 | |
|  | | | |
| **Flow cytometry** | | | |
| Brilliant Violet 510 anti-mouse CD45 | BioLegend, 103138 | 1:100 | |
| BUV805 anti-mouse CD45 | BD Horizon, 568336 | 1:100 | |
| Brilliant Violet 421 anti-mouse CD3ε | BioLegend, 100336 | 1:50 | |
| RB780 anti-mouse CD3 | BD Horizon, 755792 | 1:50 | |
| Brilliant Violet 570 anti-mouse CD8α | BioLegend, 100740 | 1:50 | |
| RB545 anti-mouse CD8 | BD Horizon, 569279 | 1:50 | |
| PE/Cyanine5 anti-mouse CD4 | BioLegend, 100514 | 1:50 | |
| APC-Fire750 anti-mouse Granzyme B | BioLegend, 372209 | 1:50 | |
| BV421 anti-mouse TNF-α | BD Horizon, 563387 | 1:50 | |
| BV750 anti-mouse IFN-gamma | BD Horizon, 566366 | 1:50 | |
| BV480 anti-mouse PD-1 | BD Horizon, 746784 | 1:100 | |
| FOXP3 monoclonal antibody | eBioscience, 14-5773-82 | 1:50 | |
| Brilliant Violet 421 anti-mouse CD11b | BioLegend, 101236 | 1:25 | |
| PE anti-mouse CD103 | BioLegend, 101406 | 1:50 | |
| Rabbit anti-human/mouse Calreticulin | Abcam, ab92516 | 1μg/mL | |
|  | | | |
| **Western Blotting** | | | |
| Antibody | **Source (Cat. No.)** | **Size (kDa)** | **Concentration** |
| Mouse anti-human β-Actin | Sigma-Aldrich, A5136 | 42 | 1:5000 (5% milk) |
| Rabbit anti-human AXL | Cell Signaling, 8661 | 130 | 1:1000 (5% BSA) |
| Rabbit anti-human phospho-AXL | R&D Systems, AF2228 | 130 | 1:1000 (5% BSA) |
| Rabbit anti-human phospho-S6 Ribosomal Protein (Ser235/236) | Cell Signaling, 4858 | 32 | 1:1000 (5% BSA) |
| Rabbit anti-human phospho-S6 Ribosomal Protein (Ser240/244) | Cell Signaling, 5364 | 32 | 1:1000 (5% BSA) |
| Rabbit anti-human phospho-eIF4B (Ser422) | Cell Signaling, 3591 | 80 | 1:1000 (5% BSA) |
| Rabbit anti-human phospho-p70 S6 Kinase (Thr389) | Cell Signaling, 9234 | 70 | 1:1000 (5% BSA) |
| Rabbit anti-human Phospho-p38 | Cell Signaling, 9211 | 38 | 1:1000 (5% BSA) |
| Rabbit anti-human Phospho-MEK | Millipore, 07-461 | 45 | 1:1000 (5% BSA) |
| Rabbit anti-human PDPK1 | Cell Signaling, 3062 | 58 to 68 | 1:1000 (5% BSA) |
| Rabbit anti-human phospho-PDPK1 | Cell Signaling, 3061 | 58 to 68 | 1:1000 (5% BSA) |
| Rabbit anti-human phospho-STING (Ser366) | Cell Signaling, 50907 | 40 | 1:1000 (5% BSA) |
| Rabbit anti-human phospho-TBK1/NAK (Ser172) | Cell Signaling, 5483 | 84 | 1:1000 (5% BSA) |
| Rabbit anti-human phospho-IRF-3 (Ser396) | Cell Signaling, 29047 | 45-55 | 1:1000 (5% BSA) |
| Rabbit anti-human/mouse HMGB1 | Abcam, ab79823 | 29 | 1:5000 (5% BSA) |
| Rabbit anti-human TNF alpha | ProteinTech, 17590-1-AP | 26 | 1:1000 (5% milk) |
| Rabbit anti-human Interferon alpha | ProteinTech, 18013-1-AP | 22 | 1:1000 (5% milk) |
| Mouse anti-human IKKα | Cell Signaling, 11930 | 85 | 1:1000 (5% BSA) |
| Rabbit anti-human IKKβ | Cell Signaling, 8943 | 87 | 1:1000 (5% BSA) |
| Rabbit anti-human phospho-IKKα/β (Ser176/180) | Cell Signaling, 2697 | 85, 87 | 1:1000 (5% BSA) |
| Rabbit anti-human phospho-NF-κB p65 (Ser536) | Cell Signaling, 3033 | 65 | 1:1000 (5% BSA) |
| Mouse anti-human IκBα | Cell Signaling, 4814 | 39 | 1:1000 (5% BSA) |
| Rabbit anti-human phospho- IκBα (Ser32) | Cell Signaling, 2859 | 40 | 1:1000 (5% BSA) |
| Rabbit anti-human NF-κB p65 | Cell Signaling, 8242 | 65 | 1:1000 (5% BSA) |
| Rabbilt anti-human Bax | Cell signaling, 5023 | 20 | 1:1000 (5% milk) |
| Rabbilt anti-human Bak | Cell signaling, 12105 | 25 | 1:1000 (5% milk) |


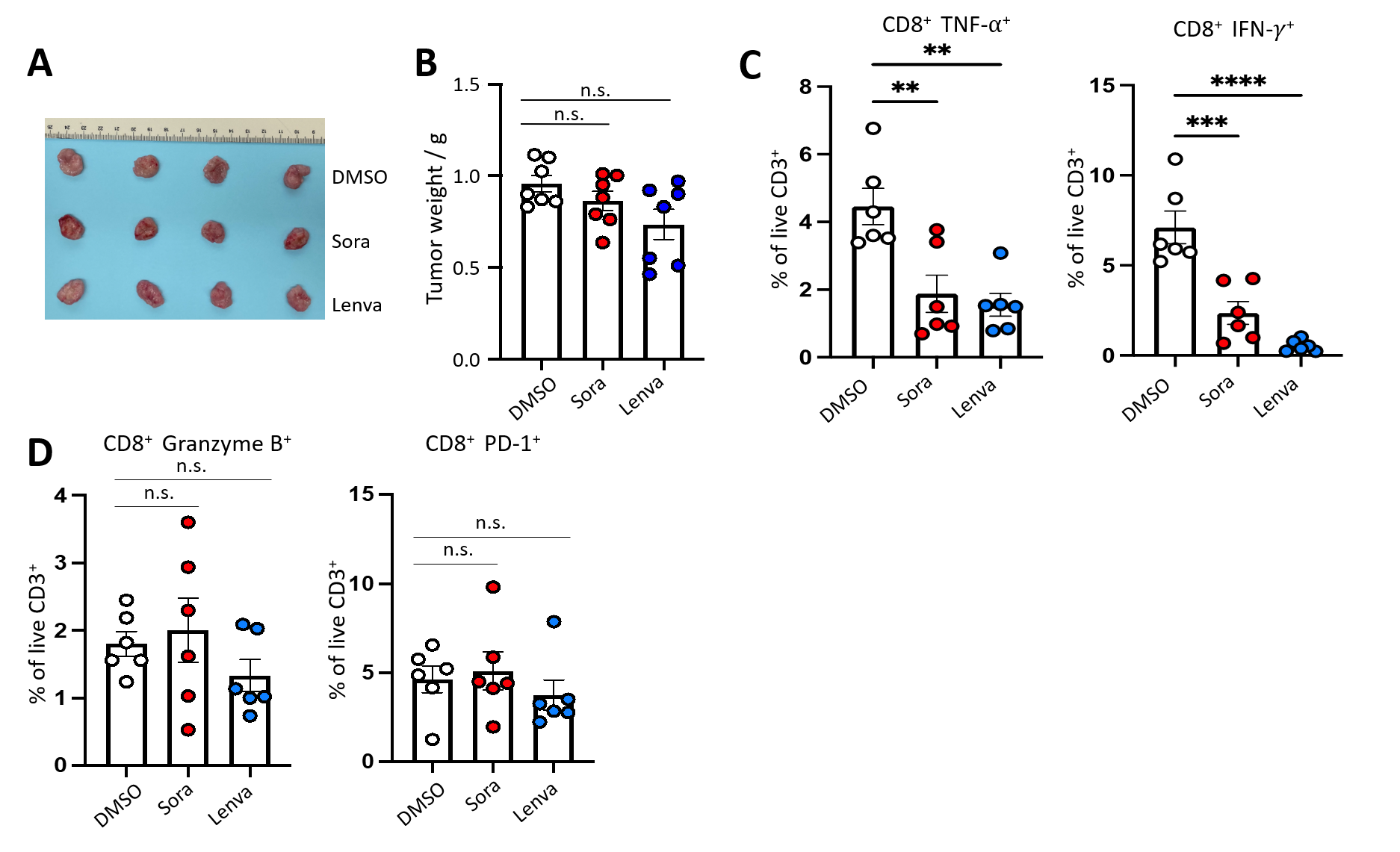


Supplemental Figure 1. (A) Representative image of xenografts resected from mice bearing RIL-175 xenografts treated with Sorafenib (Sora), Lenvatinib (Lenva) or vehicle control (DMSO). (B) Bar chart showing the residual tumor weights in mice treated with Sora, Lenva or DMSO (n = 7 per group). (C) Bar charts showing the percentages of TNF-⍺ (left) and IFN-𝛾 (right) expressing CD8^+^ T cell populations in mice treated with Sora, Lenva or DMSO (n = 6 per group). (D) Bar charts showing the percentages of Granzyme B (left) and PD-1 (right) expressing CD8^+^ T cell populations in mice treated with Sora, Lenva or DMSO (n = 6 per group). **p<0.01; ***p<0.001; ****p<0.0001; n.s. not significant on one-way ANOVA with Bonferroni’s multiple comparisons test.


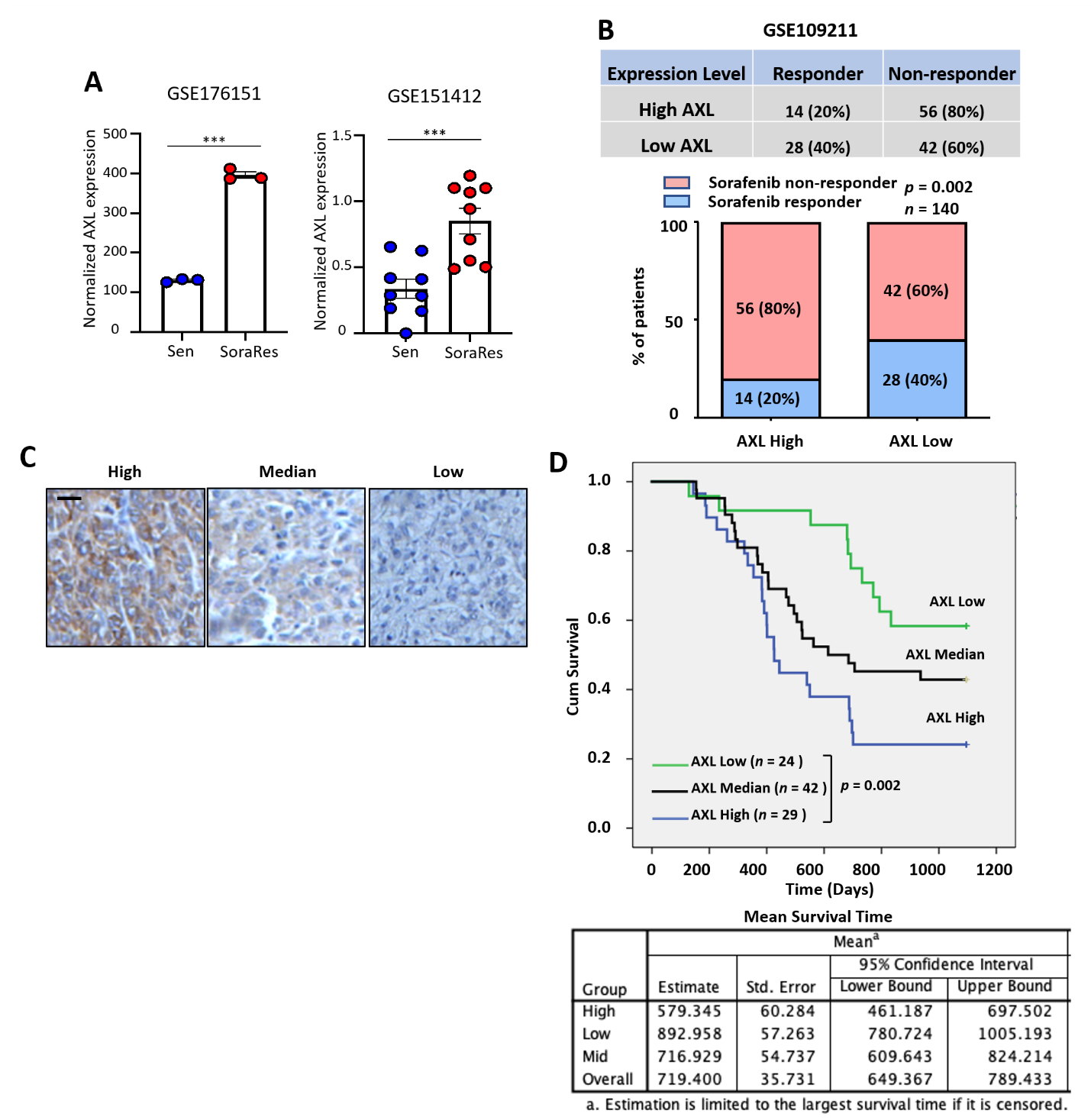


Supplemental Figure 2. (A) Normalized expression of AXL in sorafenib sensitive (Sen) and sorafenib resistant (SoraRes) samples from two datasets (GSE176151 and GSE151412). (B) Table (top) and bar chart (bottom) showing the percentage of patients with either high or low AXL expression and their corresponding response to sorafenib treatment in GSE109211 dataset. (B) Representative IHC images of various levels of AXL expression in patients receiving sorafenib treatment. Scale bar = 100 µm. (C) Kaplan-Meier survival curve (top) of patients with HCC receiving sorafenib treatment, divided into AXL high, median and low groups according to AXL expression from IHC analysis. Survival analysis was performed using the log-rank (Mantel-Cox) test. Table (bottom) summarizing the mean survival time for patients in AXL high, medium and low groups. ****p*<0.001 on a two-tailed unpaired Student’s *t*-test.


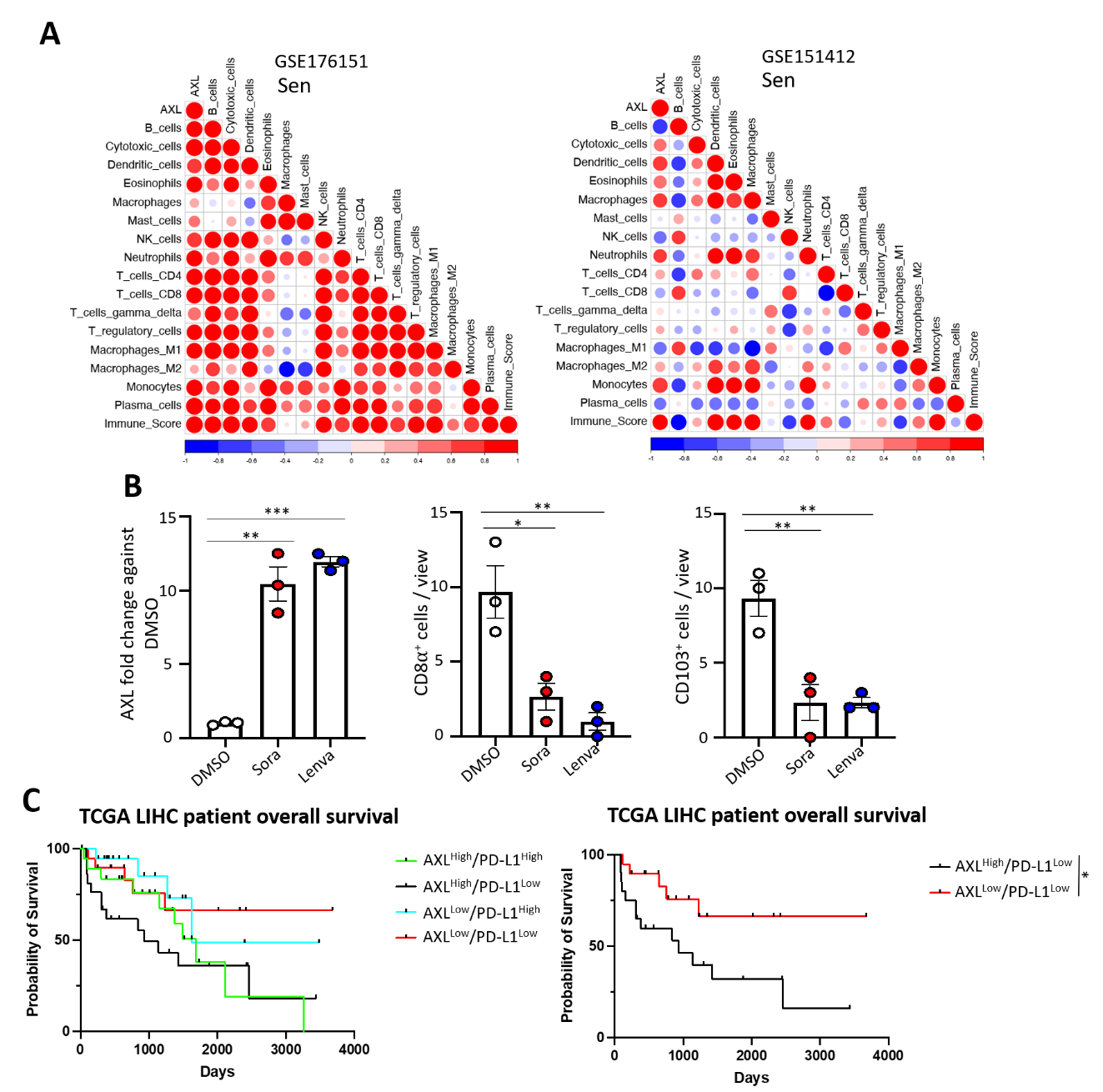


Supplemental Figure 3. (A) Bubble plots showing the correlation analysis of AXL with immune gene signatures in sorafenib sensitive samples from the datasets GSE176515 (left) and GSE151412 (right). The color scale bar and the size of the dots indicates the Pearson correlation coefficient. (B) Bar charts showing the quantification of AXL expression and CD8α^+^ and CD103^+^ cells in three independent fields. (C) Kaplan-Meier survival curves of HCC patients from TCGA-LIHC segregated into four groups according to the expression of AXL and PD-L1 (left). Survival comparison of PD-L1 low-expressing patients with either high/low AXL expression is shown (right). **p*<0.05; ***p*<0.01; ****p*<0.001 on one-way ANOVA with Bonferroni’s multiple comparisons test. **p*<0.05 on a Cox-Mantel log-rank test in the survival curve.


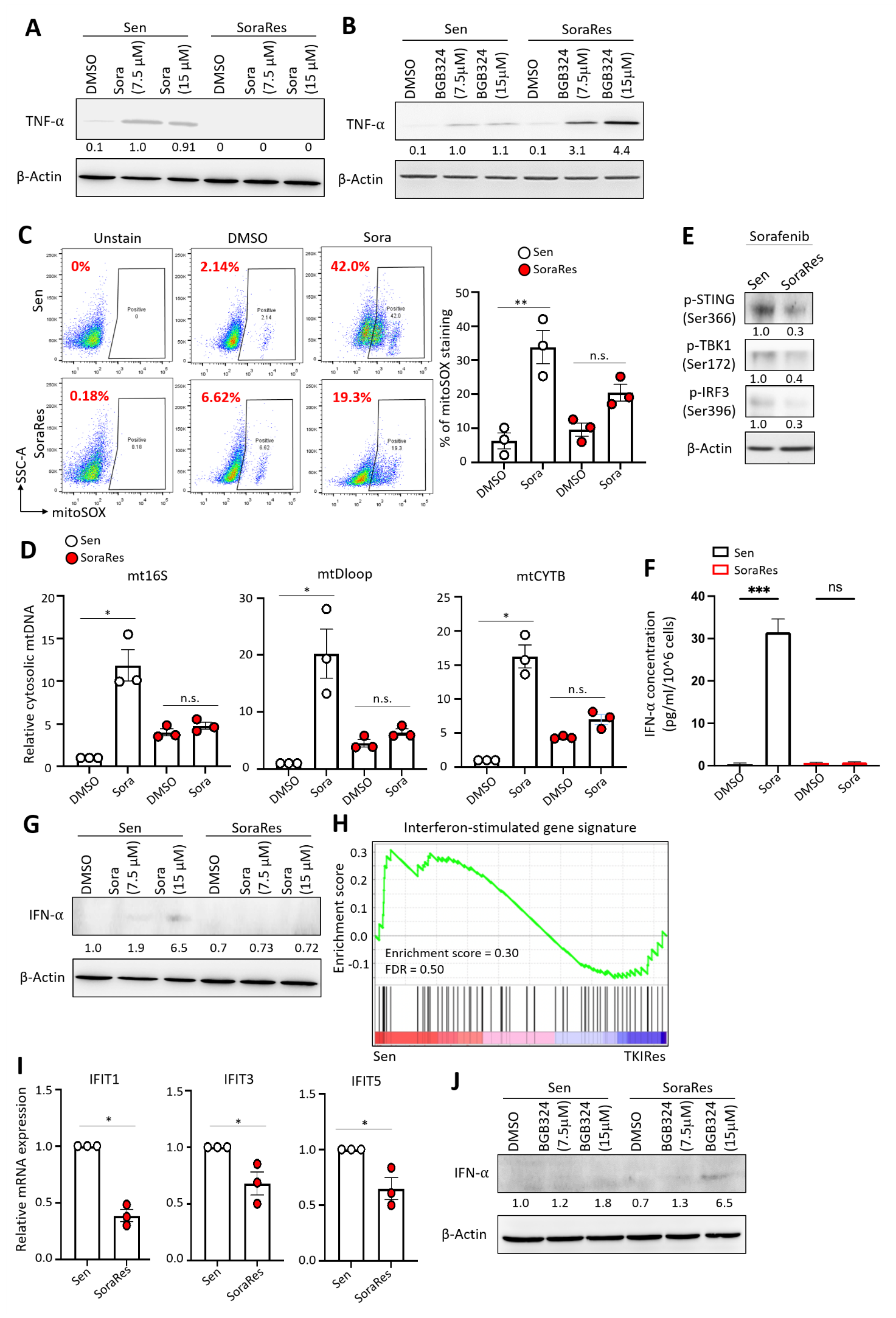


Supplemental Figure 4. (A) Western blot (WB) analysis of TNF-α in sorafenib sensitive (Sen) and sorafenib resistant (SoraRes) HepG2 cells upon sorafenib treatment. (B) WB analysis of TNF-α in Sen and SoraRes HepG2 cells upon BGB treatment. (C) Representative FACS plots (left) and percentages (right chart) of mitoSOX staining in sorafenib sensitive (Sen) and sorafenib resistant (SoraRes) HepG2 cells upon sorafenib treatment. (D) qRT-PCR quantification of mt16S, mtDloop and mtCYTB levels in the cytosol extract of Sen and SoraRes HepG2 cells upon sorafenib treatment. (E) WB analysis of STING pathway in Sen and SoraRes HepG2 cells. (F) ELISA analysis of secretory IFN-α in Sen and SoraRes cells upon sorafenib treatment. (G) WB analysis of IFN-α in Sen and SoraRes HepG2 cells upon sorafenib treatment. (H) GSEA of Sen versus TKIRes samples showing a negative correlation with interferon-stimulated gene signature. (I) qRT-PCR quantification of ISGs in Sen and SoraRes HepG2 cells. (J) WB analysis of IFN-α in Sen and SoraRes HepG2 cells treated with BGB324. **p*<0.05; ***p*<0.01; ****p*<0.001; n.s. not significant on a two-tailed unpaired Student’s *t*-test or one-way ANOVA with Bonferroni’s multiple comparisons test.


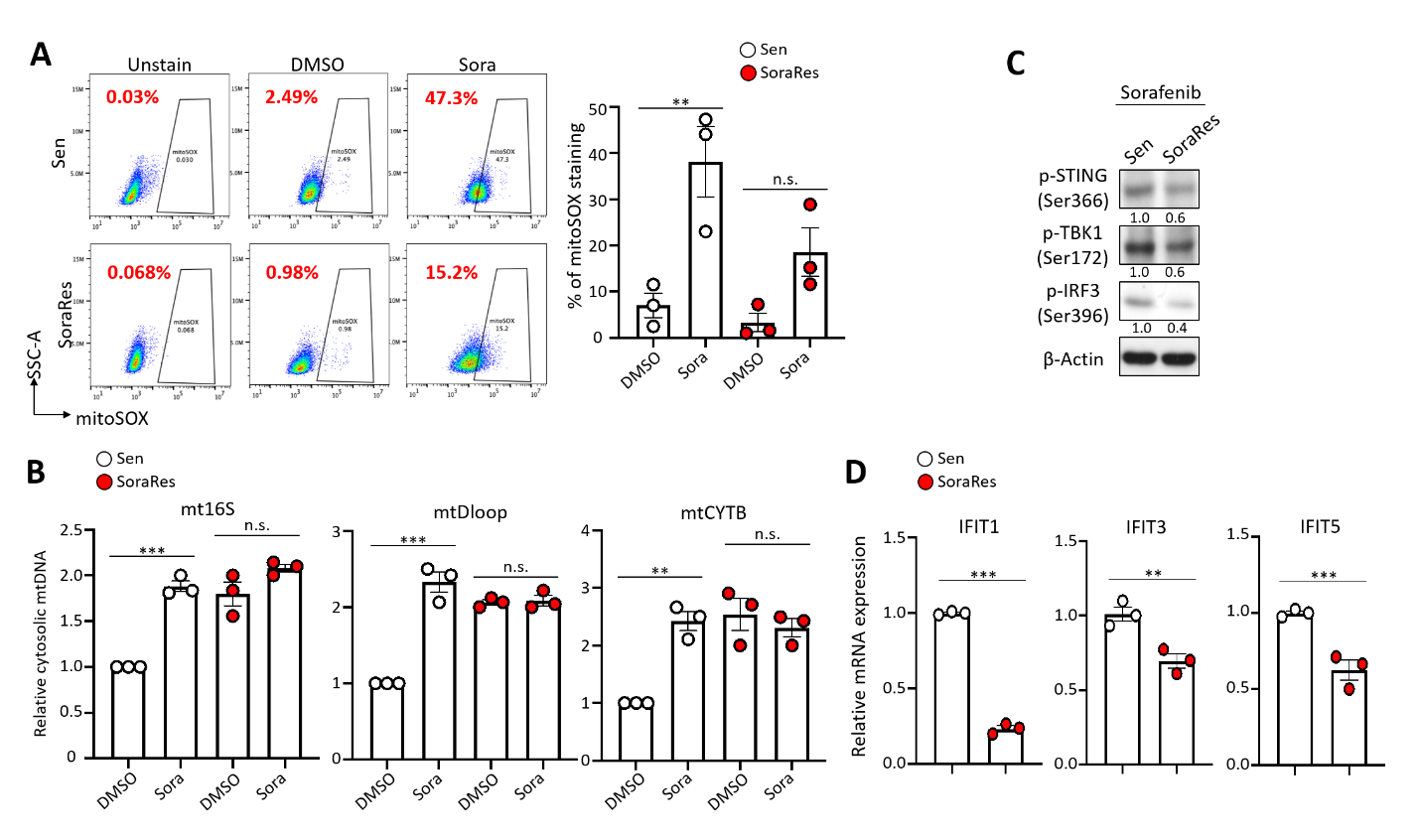


Supplemental Figure 5. (A) Representative FACS plots (left) and percentages (right chart) of mitoSOX staining in Sen and SoraRes PLC/PRF/5 cells upon sorafenib treatment. (B) qRT-PCR quantification of mt16S, mtDloop and mtCYTB levels in the cytosol extract of Sen and SoraRes PLC/PRF/5 cells upon sorafenib treatment. (C) WB analysis of STING pathway in Sen and SoraRes PLC/PRF/5 cells. (D) qRT-PCR quantification of ISGs in Sen and SoraRes PLC/PRF/5 cells. ***p*<0.01; ****p*<0.001; n.s. not significant on a two-tailed unpaired Student’s t-test.


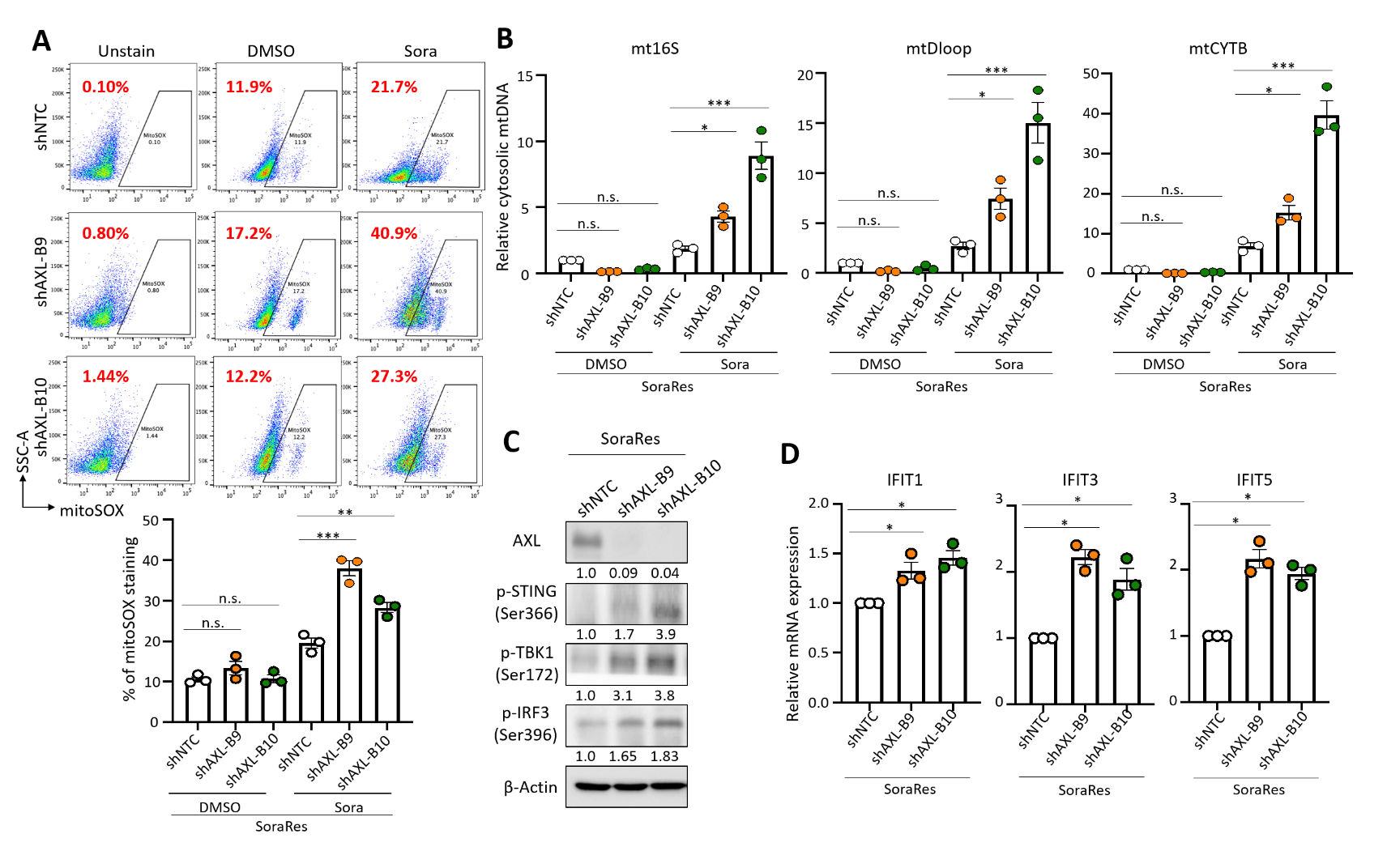


Supplemental Figure 6. (A) Representative FACS plots (top) and percentages (bottom chart) of mitoSOX staining in SoraRes HepG2 cells with non-target control (shNTC) or AXL knockdown (shAXL-B9 and shAXL-B10). (B) qRT-PCR quantification of mt16S, mtDloop and mtCYTB levels in the cytosol extract of SoraRes HepG2 cells with AXL knockdown upon sorafenib treatment. (C) WB analysis of STING pathway in SoraRes HepG2 cells with AXL knockdown. (D) qRT-PCR quantification of mt16S, mtDloop and mtCYTB levels in the cytosol extract of SoraRes HepG2 cells with AXL knockdown. **p*<0.05; ***p*<0.01; ****p*<0.001; n.s. not significant on one-way ANOVA with Bonferroni’s multiple comparisons test.


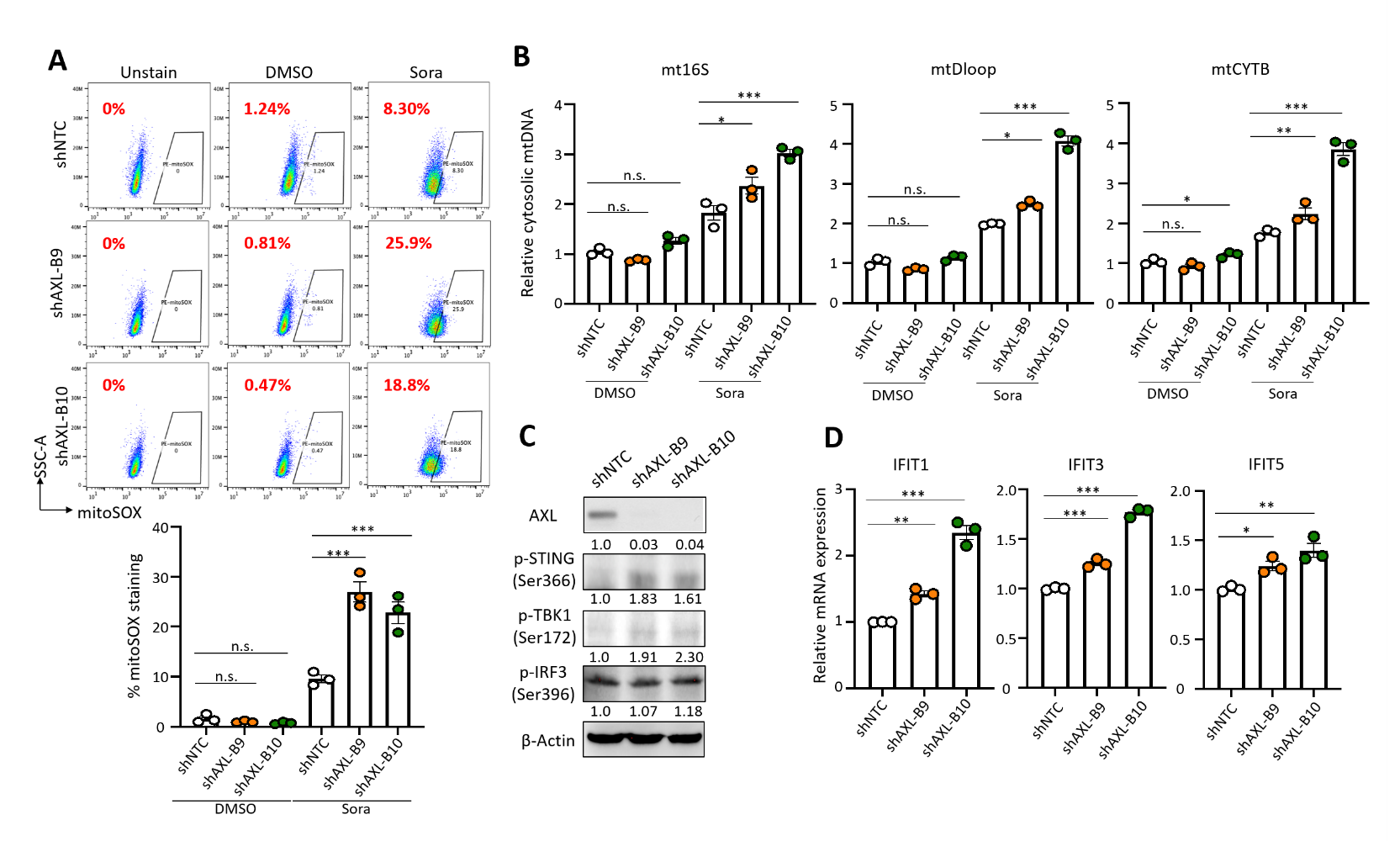


Supplemental Figure 7. (A) Representative FACS plots (top) and percentages (bottom chart) of mitoSOX staining in SoraRes PLC/PRF/5 cells with non-target control (shNTC) or AXL knockdown (shAXL-B9 and shAXL-B10). (B) qRT-PCR quantification of mt16S, mtDloop and mtCYTB levels in the cytosol extract of SoraRes PLC/PRF/5 cells with AXL knockdown upon sorafenib treatment. (C) WB analysis of STING pathway in SoraRes PLC/PRF/5 cells with AXL knockdown. (D) qRT-PCR quantification of mt16S, mtDloop and mtCYTB levels in the cytosol extract of SoraRes PLC/PRF/5 cells with AXL knockdown. **p*<0.05; ***p*<0.01; ****p*<0.001; n.s. not significant on one-way ANOVA with Bonferroni’s multiple comparisons test.


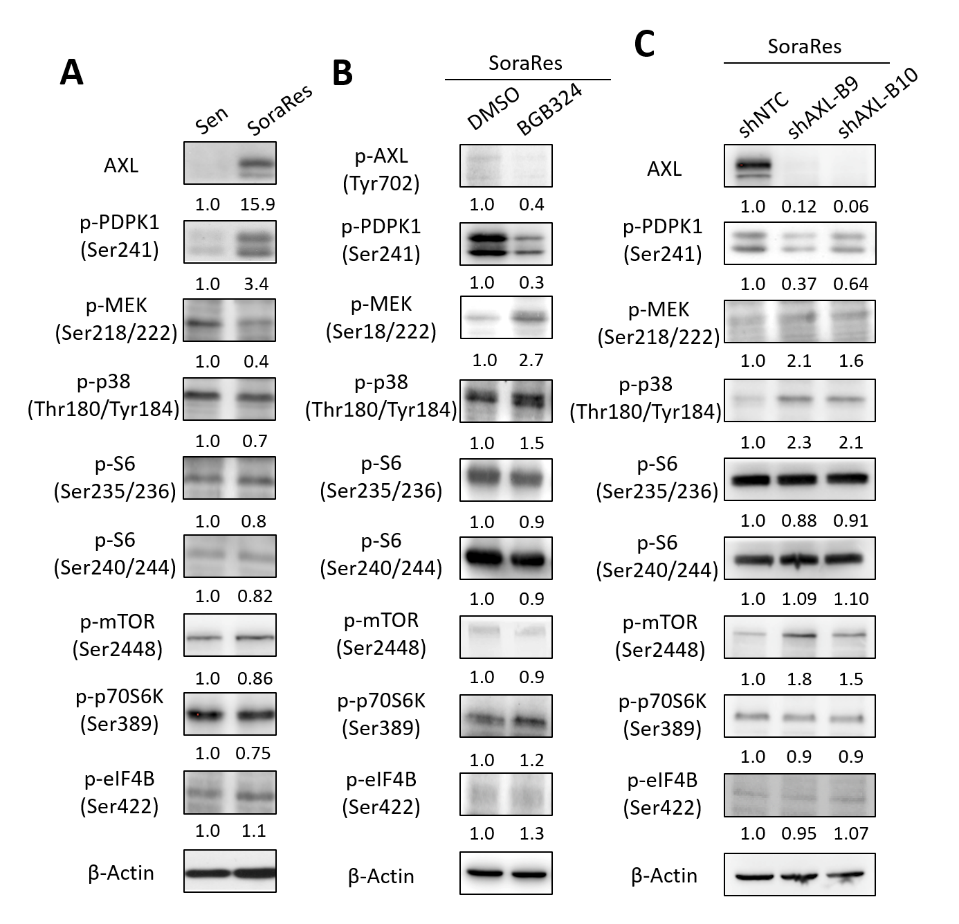


Supplemental Figure 8. (A) Western Blot (WB) analysis of AXL-mediated downstream signaling pathways in sorafenib sensitive (Sen) and sorafenib resistant (SoraRes) HepG2 cells. (B) WB analysis of AXL-mediated downstream signaling pathways in SoraRes HepG2 cells treated with BGB324. (C) WB analysis of AXL-mediated downstream signaling pathways in SoraRes HepG2 cells with AXL knockdown.


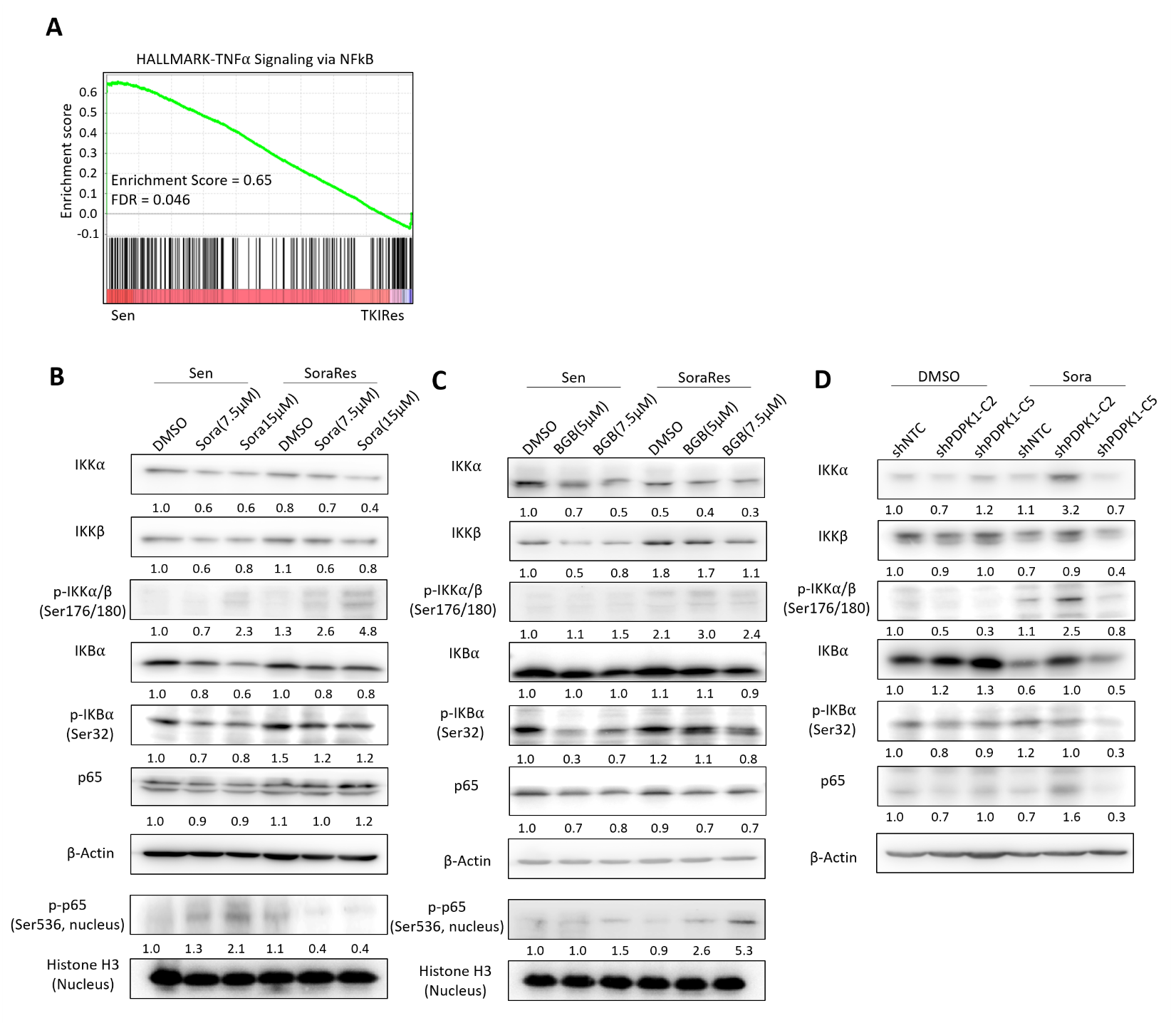


Supplemental Figure 9. (A) GSEA of Sen versus TKIRes samples showing a negative correlation with Hallmark signature of TNFα signaling via NFκB. (B) Western Blot (WB) analysis of NFκB pathway in sorafenib sensitive (Sen) and sorafenib resistant (SoraRes) HepG2 cells. (C) WB analysis of NFκB pathway in SoraRes HepG2 cells treated with BGB324. (D) WB analysis of NFκB pathway in SoraRes HepG2 cells with PDPK1 knockdown.


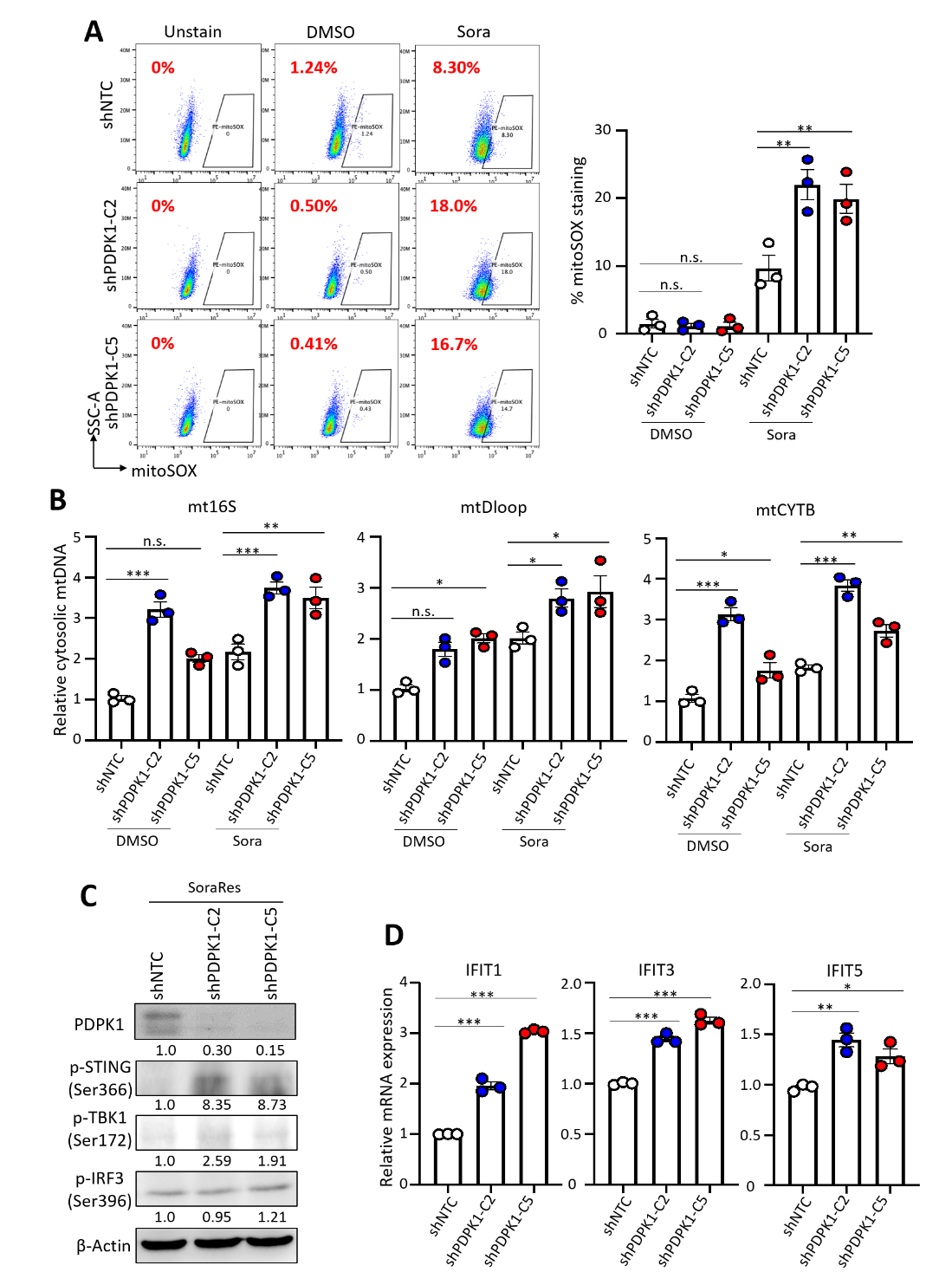


Supplemental Figure 10. (A) Representative FACS plots (top) and percentages (bottom chart) of mitoSOX staining in SoraRes PLC/PRF/5 cells with shNTC or shPDPK1-C2 and shPDPK1-C5. (B) qRT-PCR quantification of mt16S, mtDloop and mtCYTB levels in the cytosol extract of SoraRes PLC/PRF/5 cells with PDPK1 knockdown upon sorafenib treatment. (C) WB analysis of STING pathway in SoraRes PLC/PRF/5 cells with PDPK1 knockdown. (D) qRT-PCR quantification of mt16S, mtDloop and mtCYTB levels in the cytosol extract of SoraRes PLC/PRF/5 cells with PDPK1 knockdown. **p*<0.05; ***p*<0.01; ****p*<0.001; n.s. not significant on one-way ANOVA with Bonferroni’s multiple comparisons test.


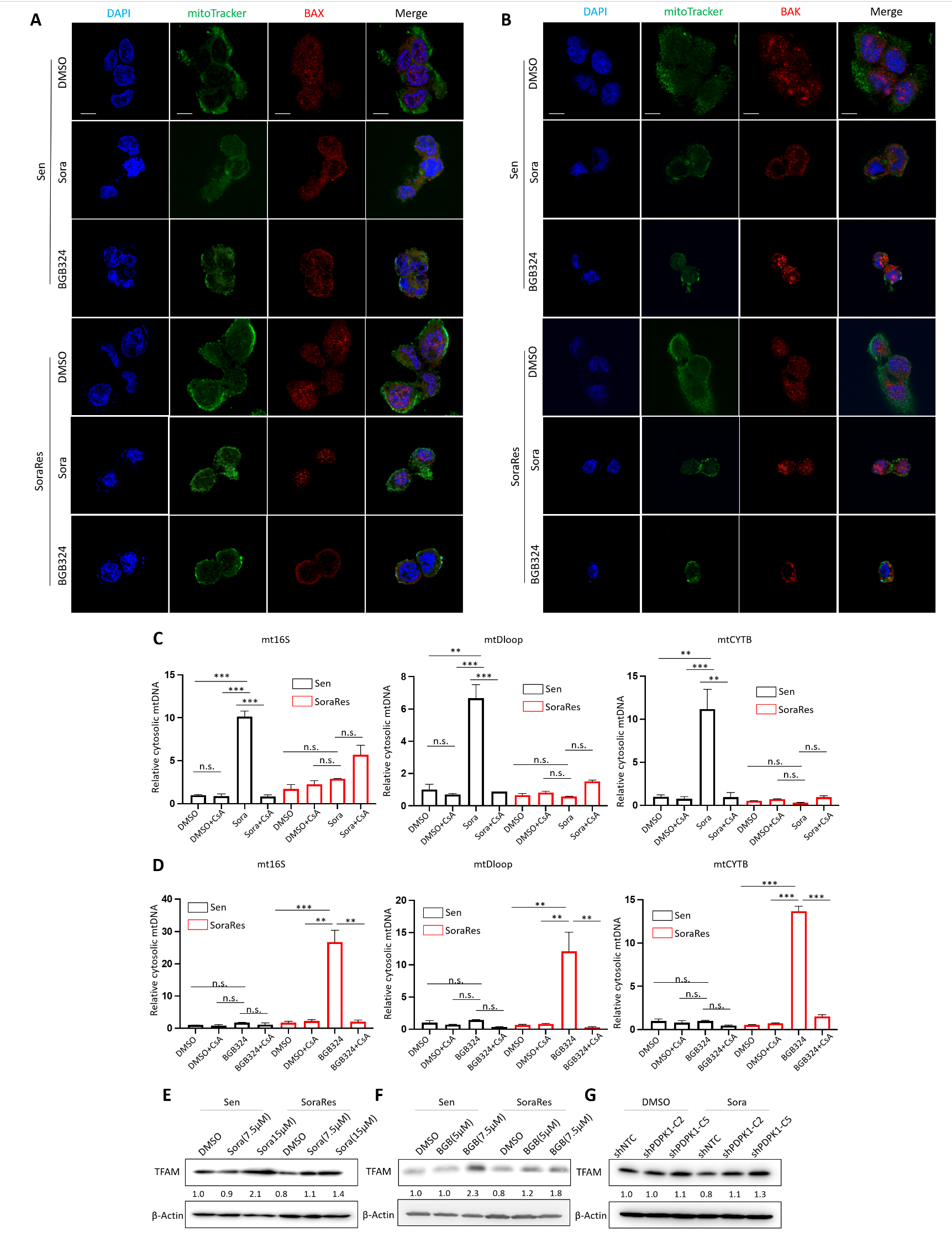


Supplemental Figure 11. (A) Representative immunofluorescence (IF) images stained with BAX, mitoTracker Green and DAPI in Sen and SoraRes cells treated with sorafenib and BGB324. (B) Representative IF images stained with BAK, mitoTracker Green and DAPI in Sen and SoraRes cells treated with sorafenib and BGB324. Scale bar = 10 um. (C) qRT-PCR quantification of mt16S, mtDloop and mtCYTB levels in the cytosol extracts of Sen and SoraRes HepG2 cells upon treatment of sorafenib and mPTP inhibitor cyclosporin A (CsA, 5ug/mL). (D) qRT-PCR quantification of mt16S, mtDloop and mtCYTB levels in the cytosol extracts of Sen and SoraRes HepG2 cells upon treatment of BGB324 and CsA (5ug/mL). (E) Western blot (WB) analysis of TFAM in Sen and SoraRes HepG2 cells treated with DMSO or sorafenib. (F) WB analysis of TFAM in Sen and SoraRes HepG2 cells treated with DMSO or BGB324. (G) WB analysis of TFAM in SoraRes HepG2 cells with PDPK1 knockdown. **p<0.01; ***p<0.001; n.s. not significant on one-way ANOVA with Bonferroni’s multiple comparisons test.


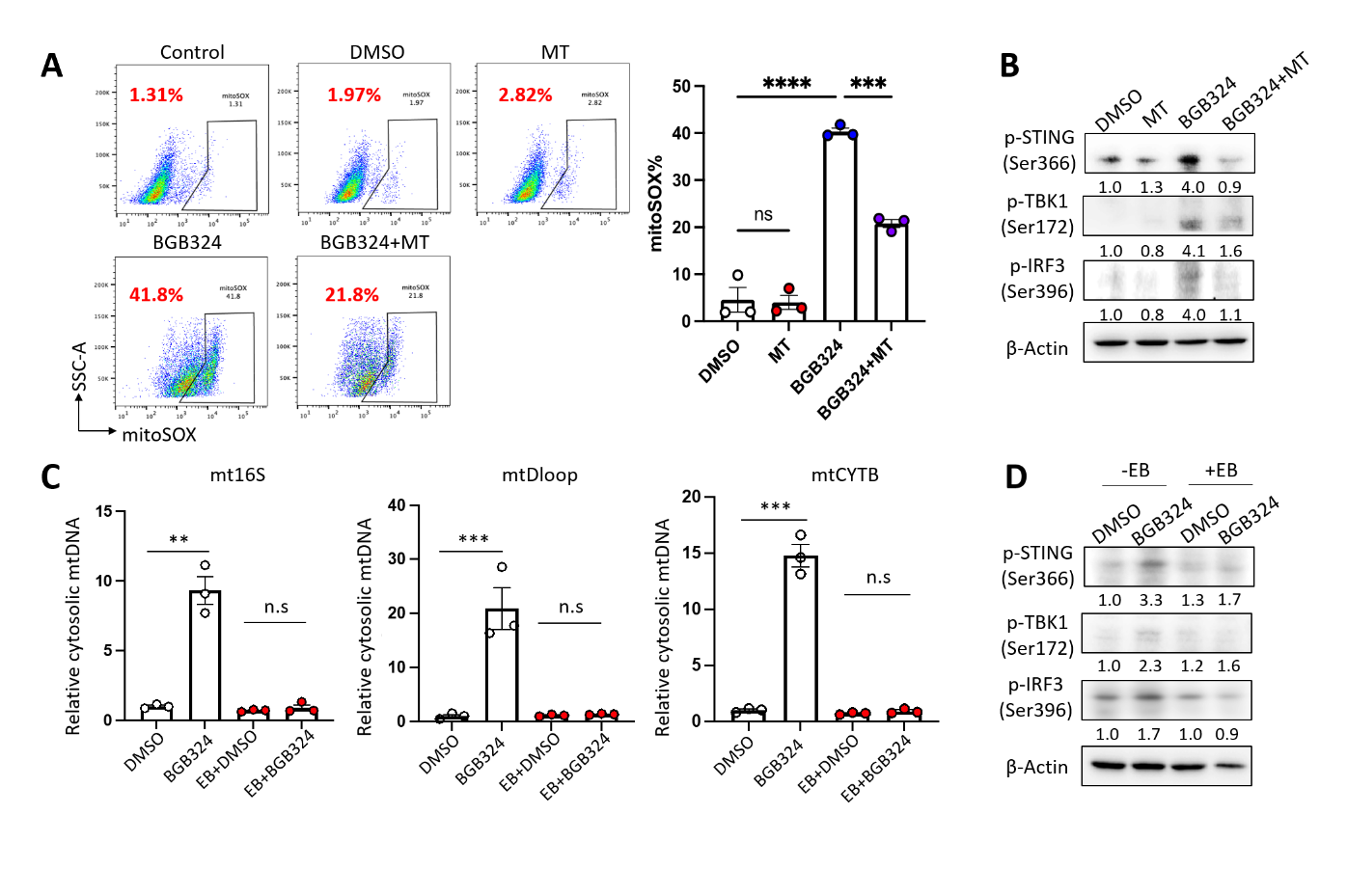


Supplemental Figure 12. (A) Flow cytometry analysis of mitochondrial ROS levels in SoraRes HepG2 cells treated with mitoTEMPO (MT) (0.5mM). (B) Western blot analysis of STING pathway in SoraRes HepG2 cells under BGB324 treatment with/without MT treatment. (C) qPCR analysis of mtDNA levels in SoraRes HepG2 cells with/without BGB324 and ethidium bromide (EB) treatment. (D) Western blot analysis of STING pathway in SoraRes HepG2 cells with/without BGB324 and EB treatment. **p<0.01; ***p<0.001; ****p<0.0001; n.s. not significant on one-way ANOVA with Bonferroni’s multiple comparisons test.


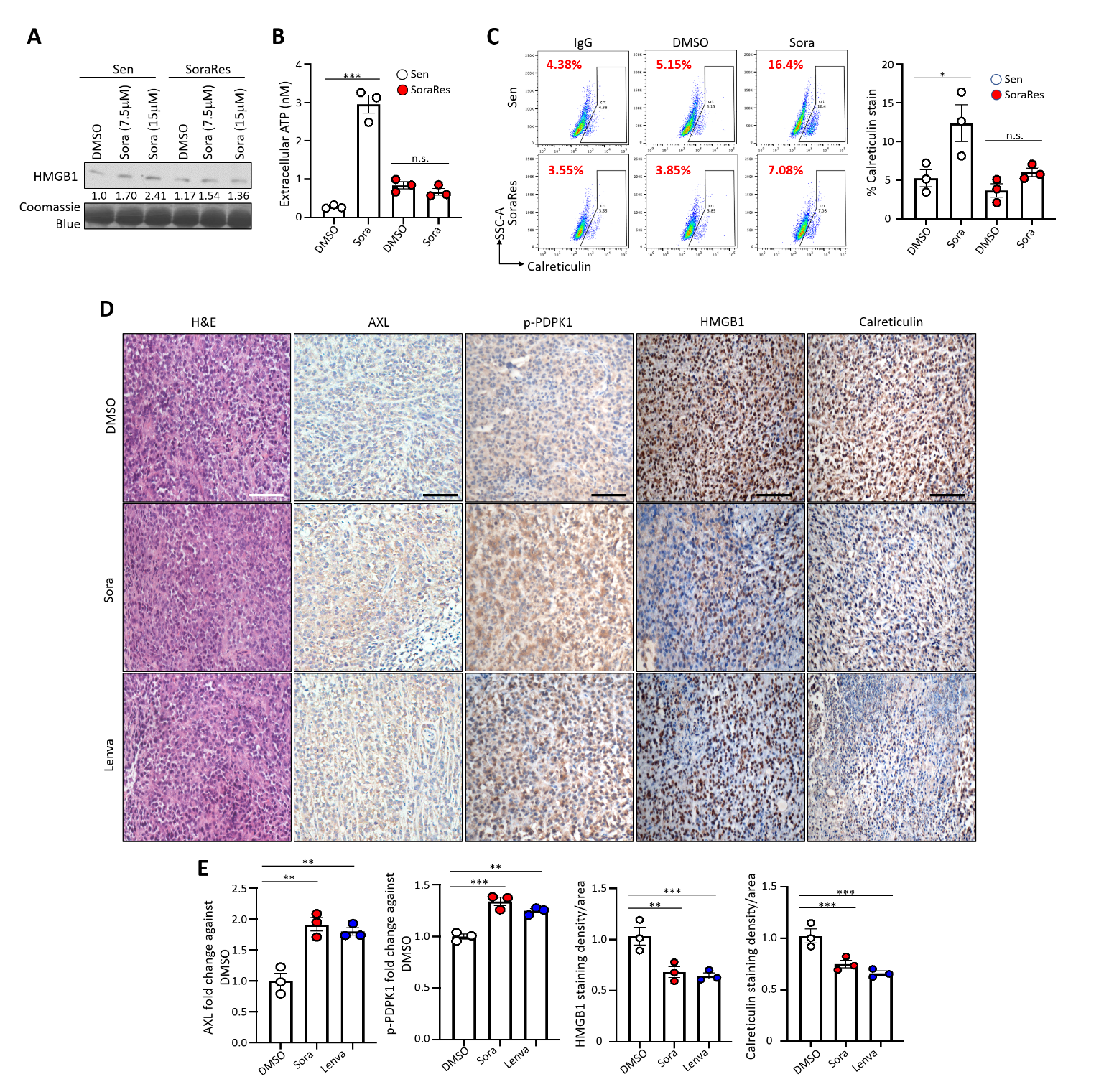


Supplemental Figure 13. (A) WB analysis of secretory HMGB1 in the conditioned media of SoraRes PLC/PRF/5 cells upon sorafenib treatment at the indicated concentrations. (B) ATP concentrations in the conditioned media collected from Sen and SoraRes PLC/PRF/5 cells upon sorafenib treatment. (C) Representative FACS plots (left) and percentages (right chart) of membrane calreticulin expression in Sen and SoraRes PLC/PRF/5 cells upon sorafenib treatment. (D) Representative H&E and IHC images showing AXL, p-PDPK1, HMGB1 and calreticulin expression in the tumor sections of C57BL/6N mice bearing RIL-175 xenografts treated with Sorafenib (Sora), Lenvatinib (Lenva) or vehicle control (DMSO). Scale bar = 100 µm. (E) Bar charts showing the quantification of AXL, p-PDPK1, HMGB1 and calreticulin staining intensities in three independent fields. ***p*<0.01; ****p*<0.001 on one-way ANOVA with Bonferroni’s multiple comparisons test.


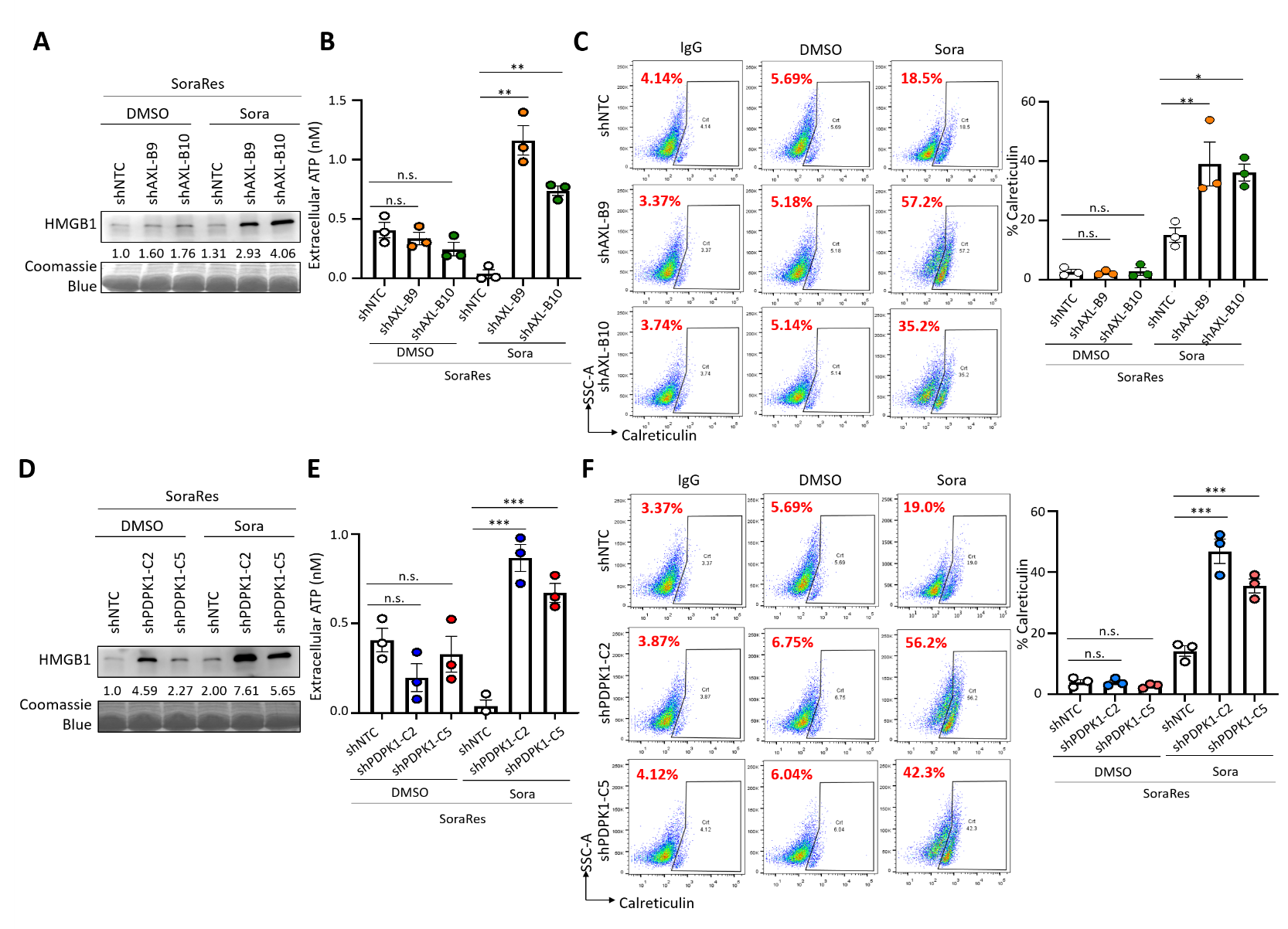


Supplemental Figure 14. (A) WB analysis of secretory HMGB1 in the conditioned media of SoraRes HepG2 cells with AXL knockdown upon sorafenib treatment. (B) ATP concentrations in the conditioned media collected from SoraRes HepG2 cells with AXL knockdown upon sorafenib treatment. (C) Representative FACS plots (left) and percentages (right chart) of membrane calreticulin expression in SoraRes HepG2 cells with AXL knockdown upon sorafenib treatment. (D) WB analysis of secretory HMGB1 in the conditioned media of SoraRes HepG2 cells with PDPK1 knockdown upon sorafenib treatment. (E) ATP concentrations in the conditioned media collected from SoraRes HepG2 cells with PDPK1 knockdown upon sorafenib treatment. (F) Representative FACS plots (left) and percentages (right chart) of membrane calreticulin expression in SoraRes HepG2 cells with PDPK1 knockdown upon sorafenib treatment. **p*<0.05; ***p*<0.01; ****p*<0.001; n.s. not significant on one-way ANOVA with Bonferroni’s multiple comparisons test.


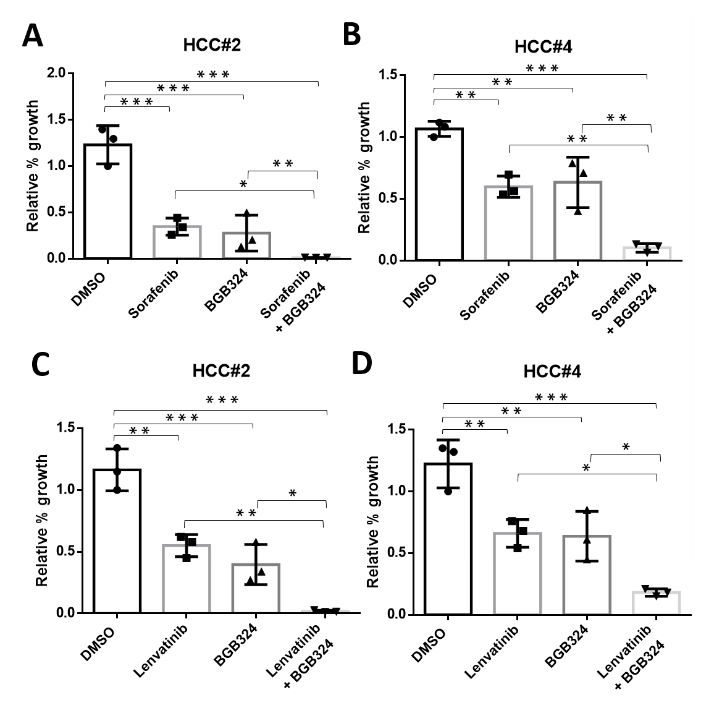


Supplemental Figure 15. (A-B) Relative percentage growth of HCC patient-derived organoids upon sorafenib, BGB324 or combined treatment. (C-D) Relative percentage growth of HCC patient-derived organoids upon lenvatinib, BGB324 or combined treatment.


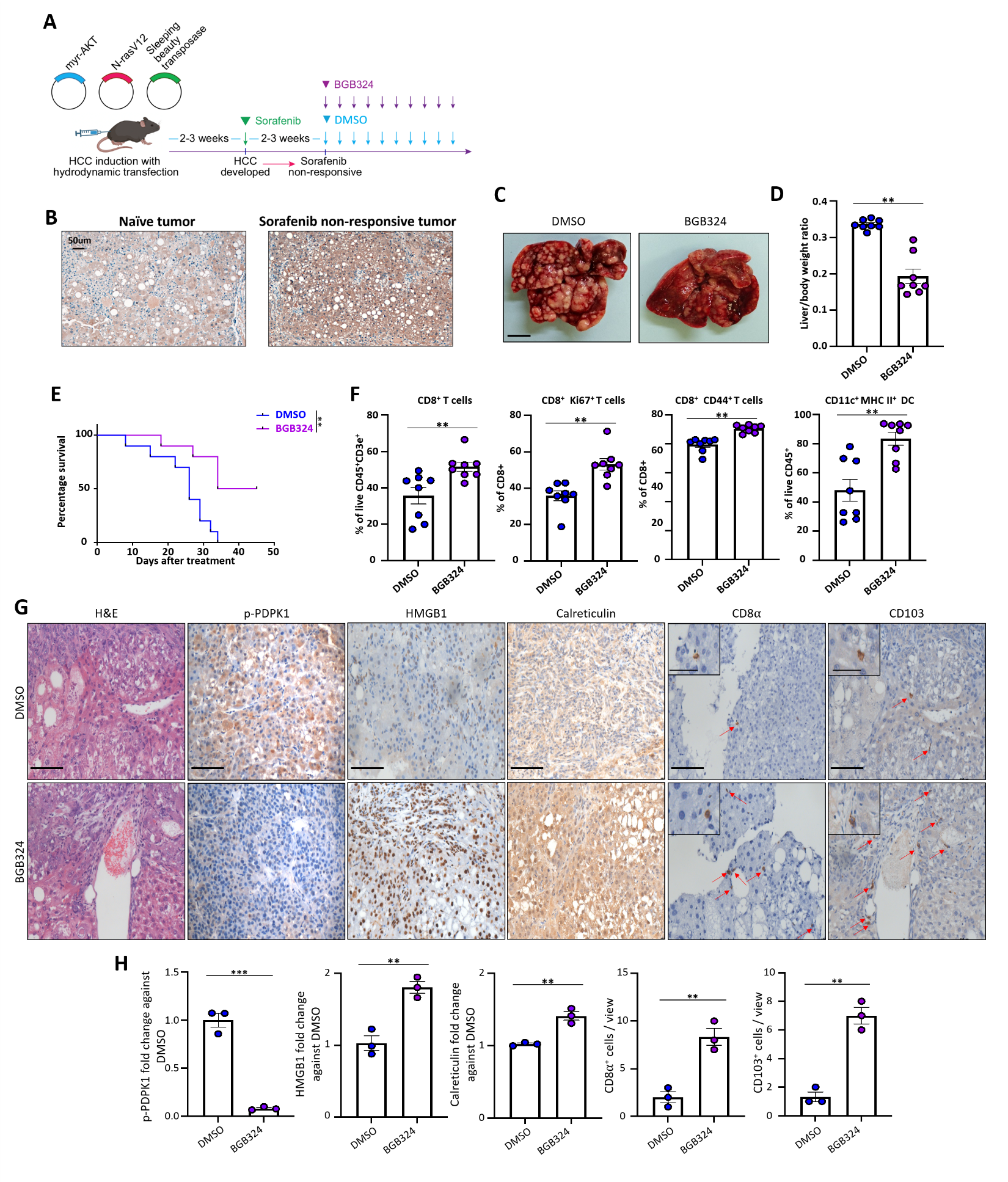
 Supplemental Figure 16. (A) Schematic diagram illustrating the treatment scheme of BGB324 and vehicle (DMSO) control groups in sorafenib non-responsive spontaneous liver tumors established by hydrodynamic tail vein injection of oncogenic plasmids. (B) Representative IHC images showing AXL expression in treatment-naïve and sorafenib non-responsive tumor tissues. Scale bar = 50 µm. (C) Representative images of livers resected from mice receiving either DMSO or BGB324 treatment. Scale bar = 1 cm. (D) Liver to body weight ratio of mice after treatment (n = 8 per group). Data representative of one experiment. (E) Survival curve of mice treated with BGB324 and DMSO. (F) Bar charts showing the percentages of intratumoral immune cell populations and CD8+/Foxp3+ ratio in mice treated with BGB324 or DMSO. (G) Representative H&E and IHC images showing p-PDPK1, HMGB1, calreticulin, CD8α and CD103 expression in the tumor sections after BGB324 and DMSO treatment. Scale bar = 100 µm and 50 µm (inset). Red arrows indicating positive signals of CD8α and CD103. (H) Bar charts showing the quantification of p-PDPK1, HMGB1, calreticulin staining intensities, and CD8α+ and CD103+ cells in three independent fields. **p<0.01 on a Cox-Mantel log-rank test in the survival curve. **p<0.01; ***p<0.001 on a two-tailed unpaired Student’s t-test. **p*<0.05; ***p*<0.01; ****p*<0.001 on one-way ANOVA with Bonferroni’s multiple comparisons test.


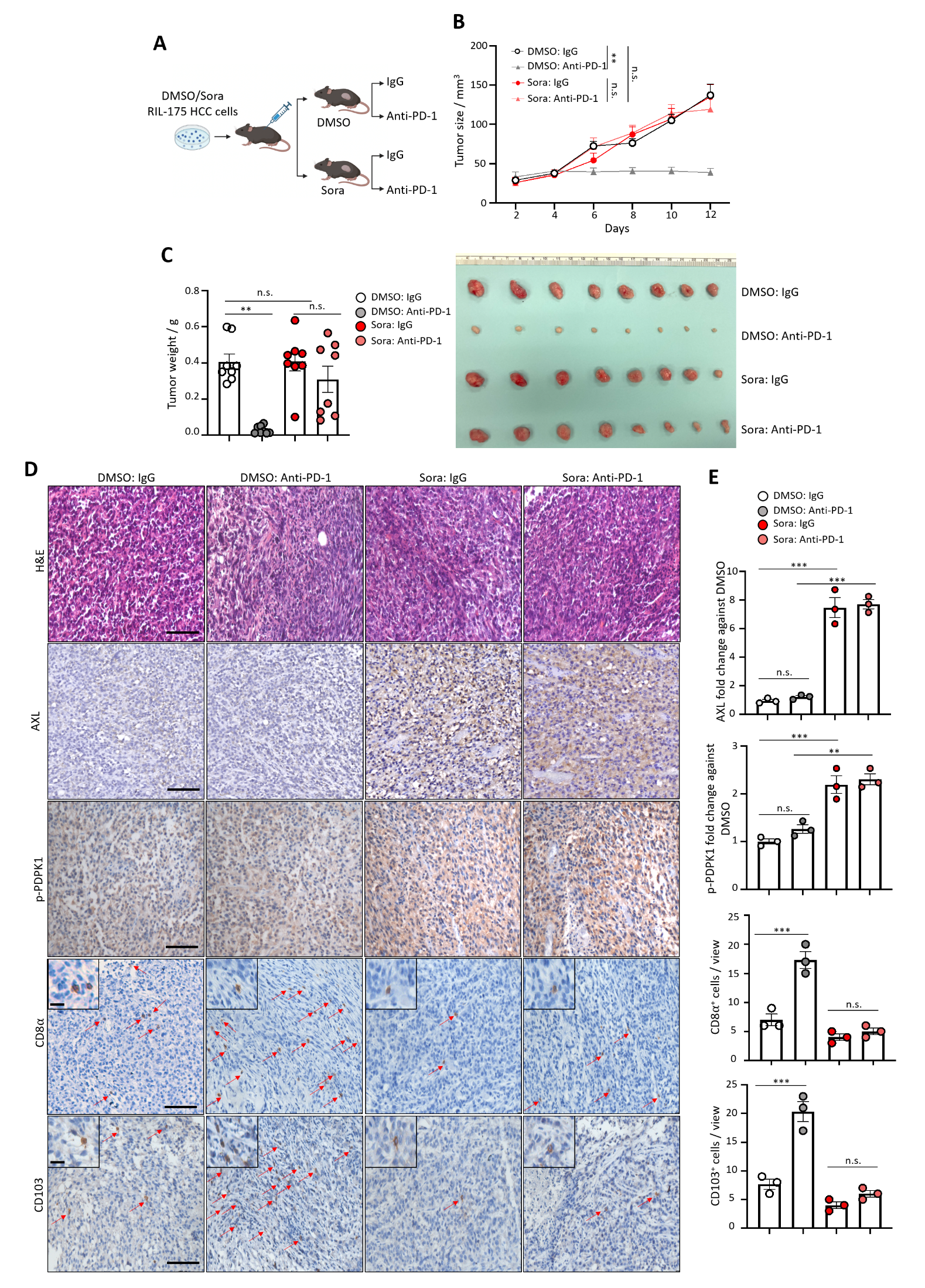


Supplemental Figure 17. (A) Schematic diagram illustrating anti-PD-1 and IgG treatment in secondary xenografts established from residual RIL-175 HCC cells after treatment with sorafenib or DMSO. (B) Tumor growth curves across the treatment course (n = 8 per group). Data representative of one experiment. (C) Tumor weight of resected xenografts after treatment. (D) Representative image of xenografts resected after treatment. (D) Representative H&E and IHC images showing AXL, p-PDPK1, and CD8α^+^ and CD103^+^ cells in the tumor sections from DMSO and Sora groups treated with either IgG or anti-PD-1 antibodies. Scale bar = 100 µm and 25 µm (inset). Red arrows indicating positive signals of CD8α and CD103. (E) Bar charts showing the quantification of CD8α+ and CD103+ cells in three independent fields. ***p*<0.01; ****p*<0.001; n.s. not significant on one-way ANOVA with Bonferroni’s multiple comparisons test.


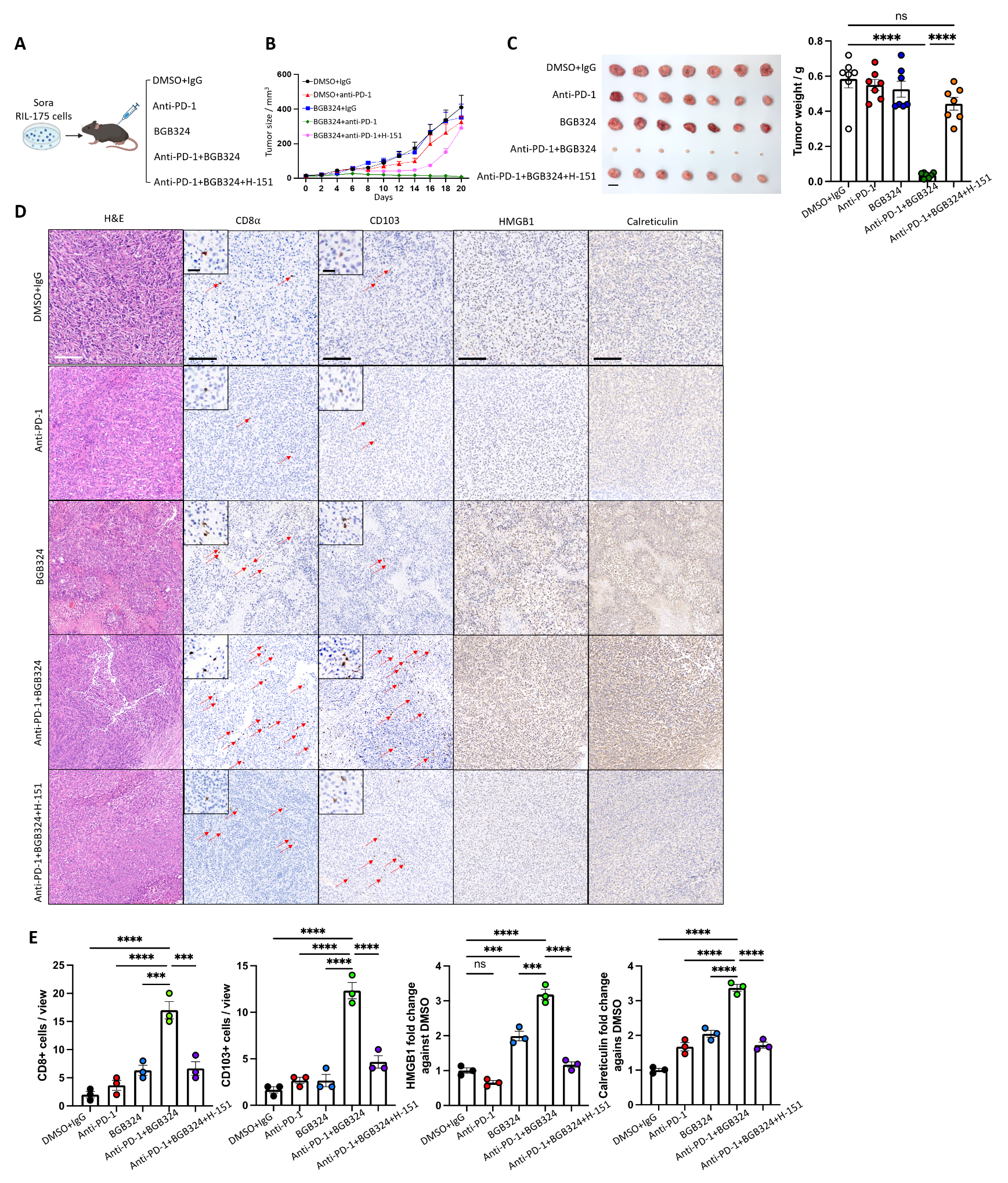


Supplemental Figure 18. (A) Schematic diagram illustrating single and combined anti-PD-1 and BGB324 treatment rescued with STING inhibitor H-151 in secondary xenografts established from residual RIL-175 HCC cells after sorafenib treatment. (B) Tumor growth curves across the treatment course (n = 7 per group). Data representative of one experiment. (C) Representative image of resected residual tumor nodules (left). Bar chart showing the tumor weight of resected xenografts after treatment (right). (D) Representative H&E and IHC images showing p-PDPK1, HMGB1, calreticulin, CD8α and CD103 expression in the tumor sections after single anti-PD-1 antibody, single BGB324, combined treatment and combined treatment rescued with H-151. Scale bar = 100 µm and 25 µm (inset). Red arrows indicating positive signals of CD8α and CD103. (E) Bar charts showing the quantification of HMGB1 and calreticulin staining intensities, and CD8α^+^ and CD103^+^ cells in three independent fields. ****p*<0.001; *****p*<0.0001; n.s. not significant on one-way ANOVA with Bonferroni’s multiple comparisons test.


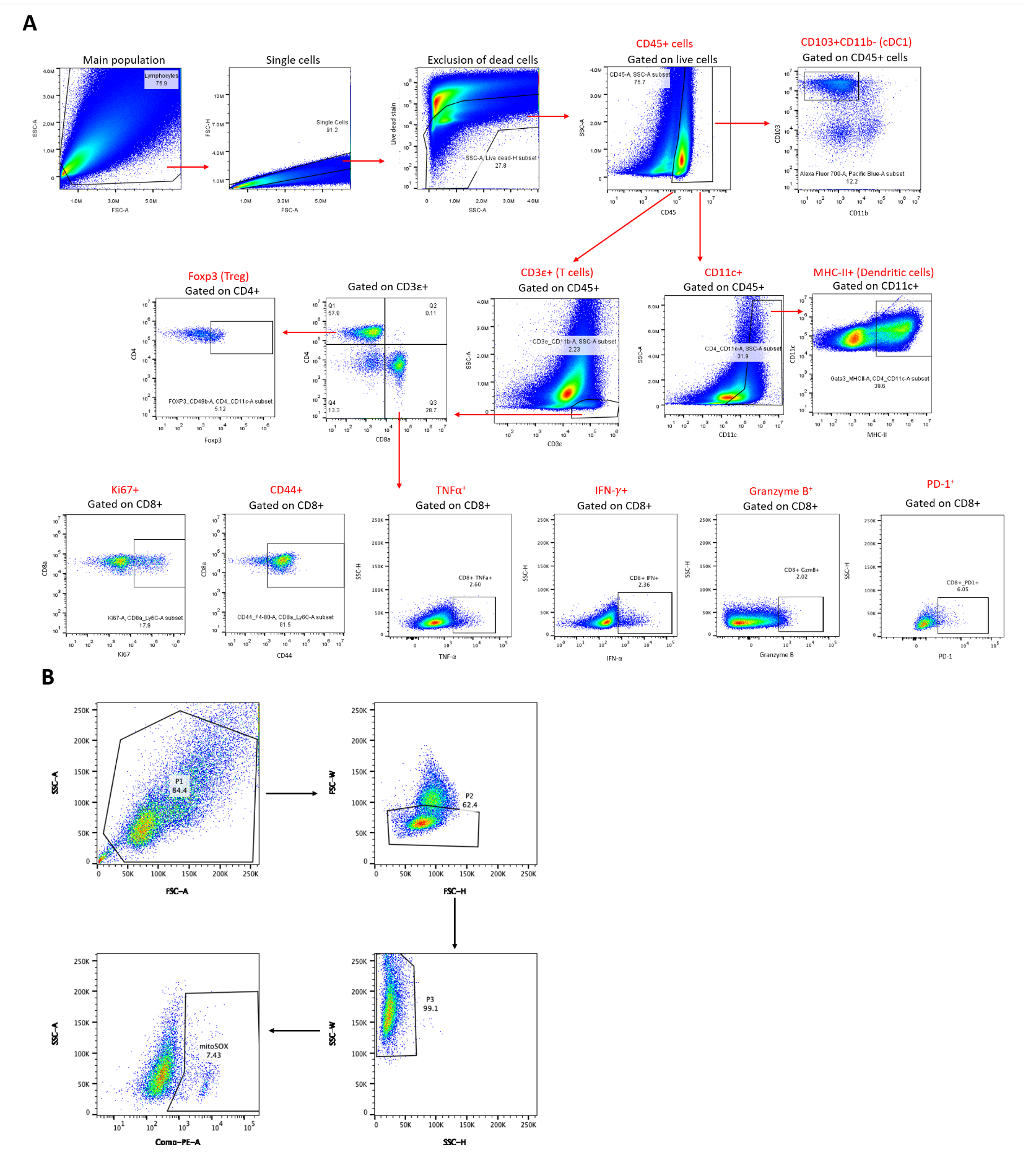


Supplemental Figure 19. (A) Gating strategy for immunophenotyping of tumor infiltrating immune cells. (B) Gating strategy for HCC cells with mitoSOX staining.
