# Supplementary material for: Targeting AXL induces tumor-intrinsic immunogenic response in tyrosine kinase inhibitor-resistant liver cancer
Source: Cell Death Dis. 2024 Feb 3;15(2):110. doi: 10.1038/s41419-024-06493-0 (PMC10838288; doi:10.1038/s41419-024-06493-0)

Main Figure 3F

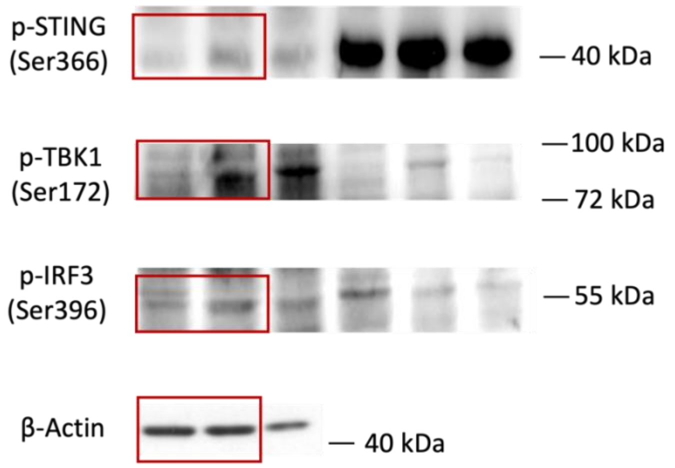

Main Figure 4A

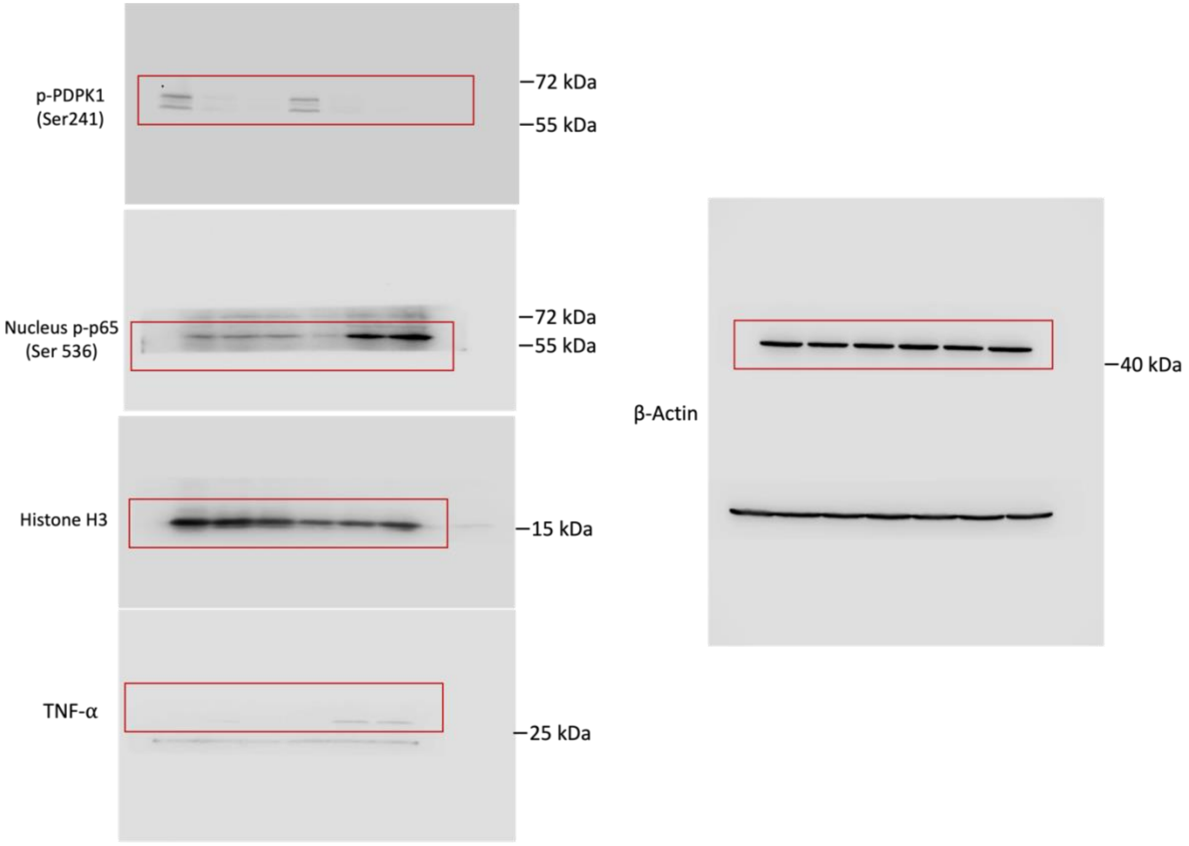

Main Figure 4E

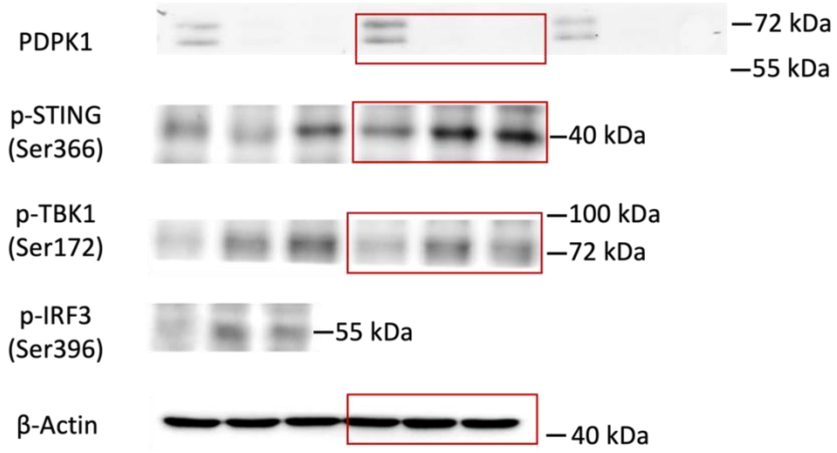

Main Figure 4F

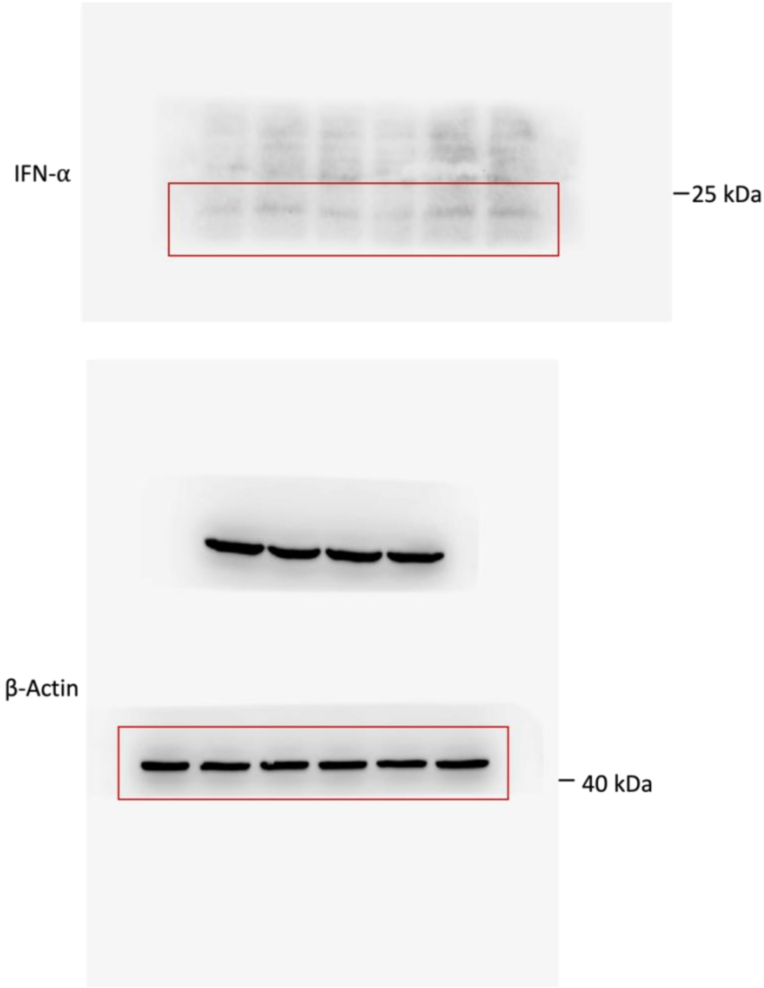

Main Figure 5A

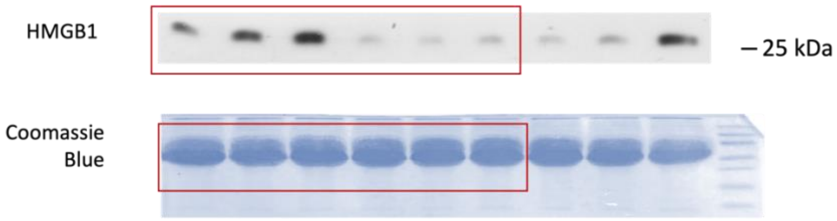

Main Figure 5D

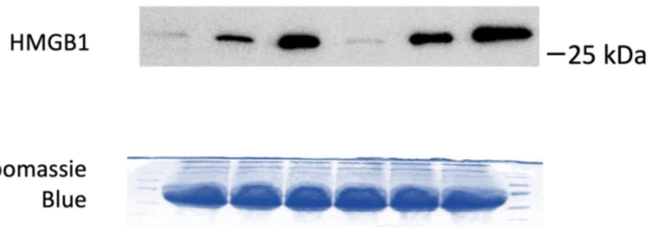

Main Figure 5G

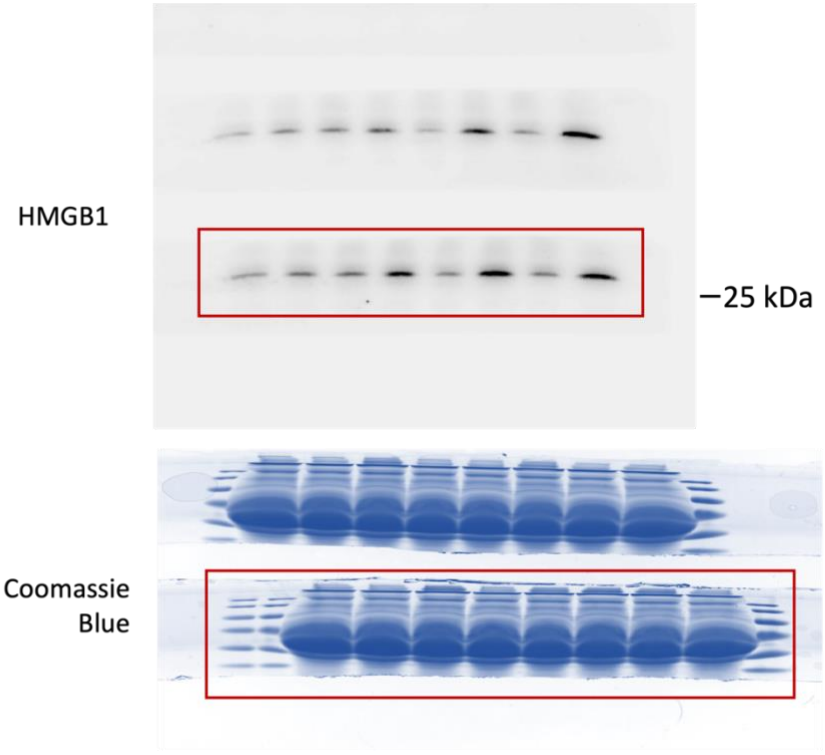

Supplementary Figure 4A

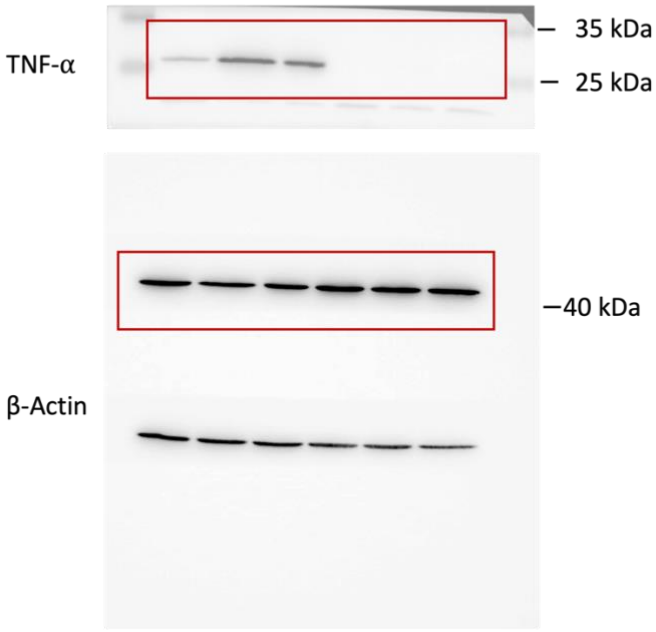

Supplementary Figure 4B

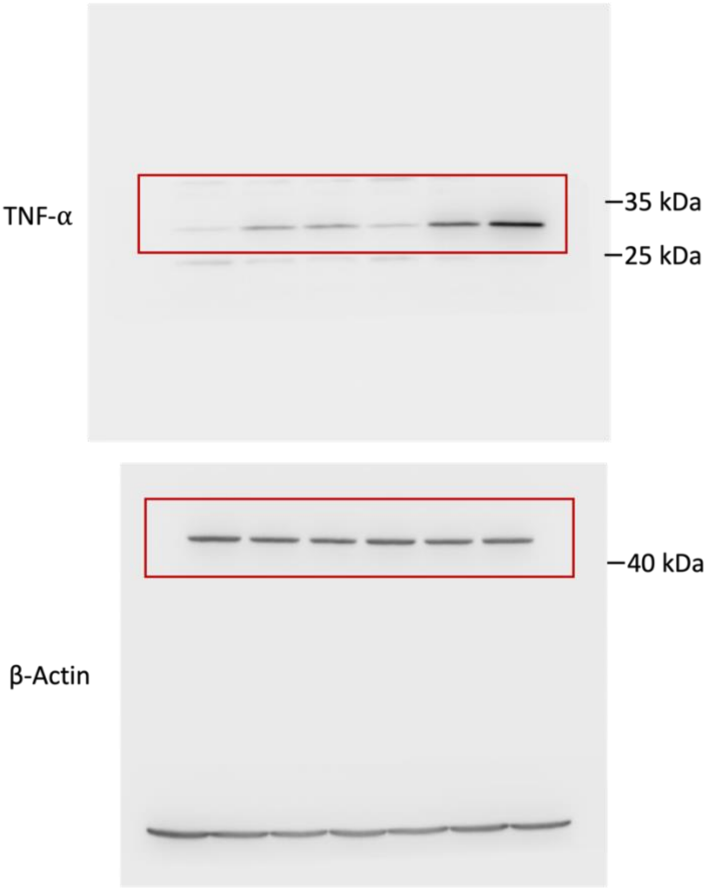

Supplementary Figure 4E

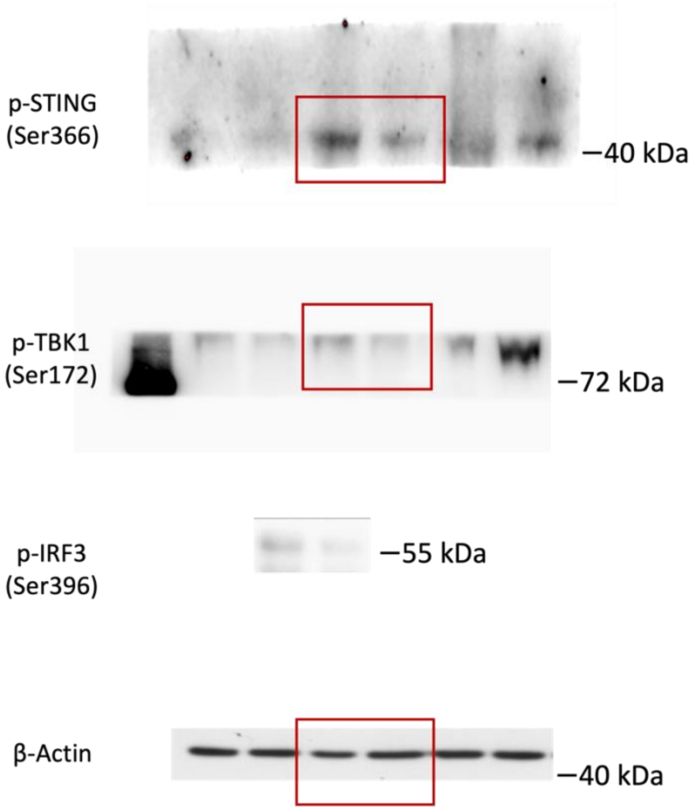

Supplementary Figure 4G

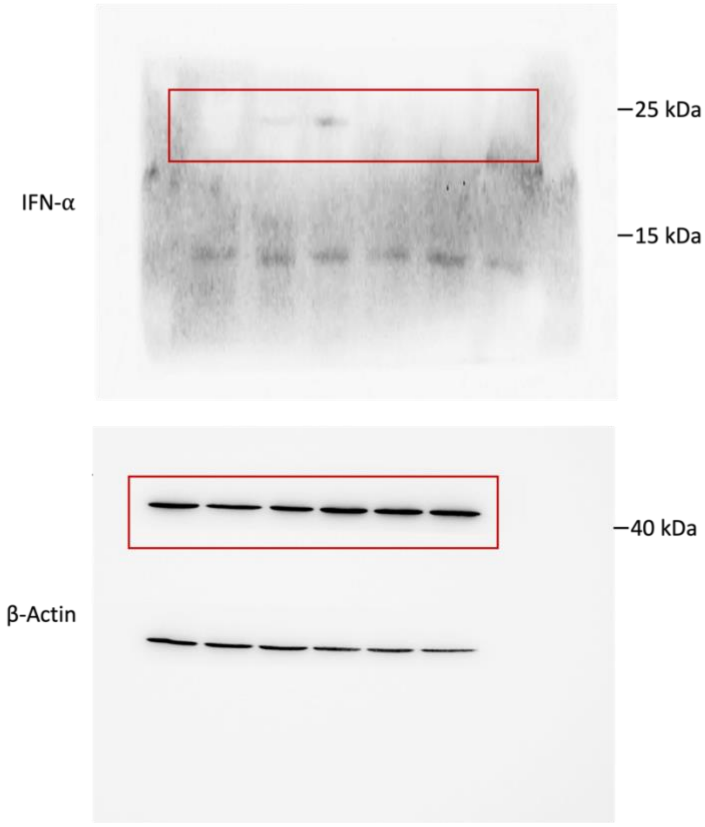

Supplementary Figure 4J

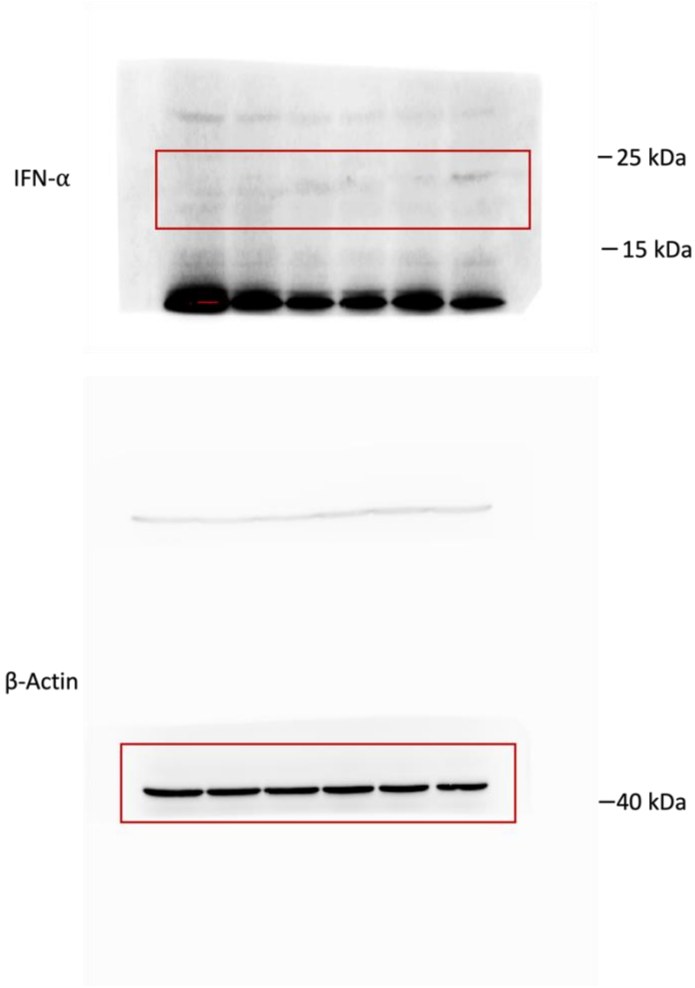

Supplementary Figure 5C

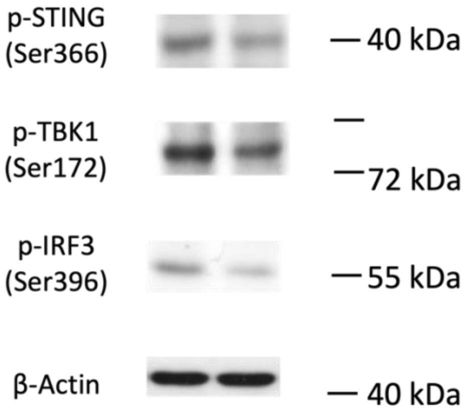

Supplementary Figure 6C

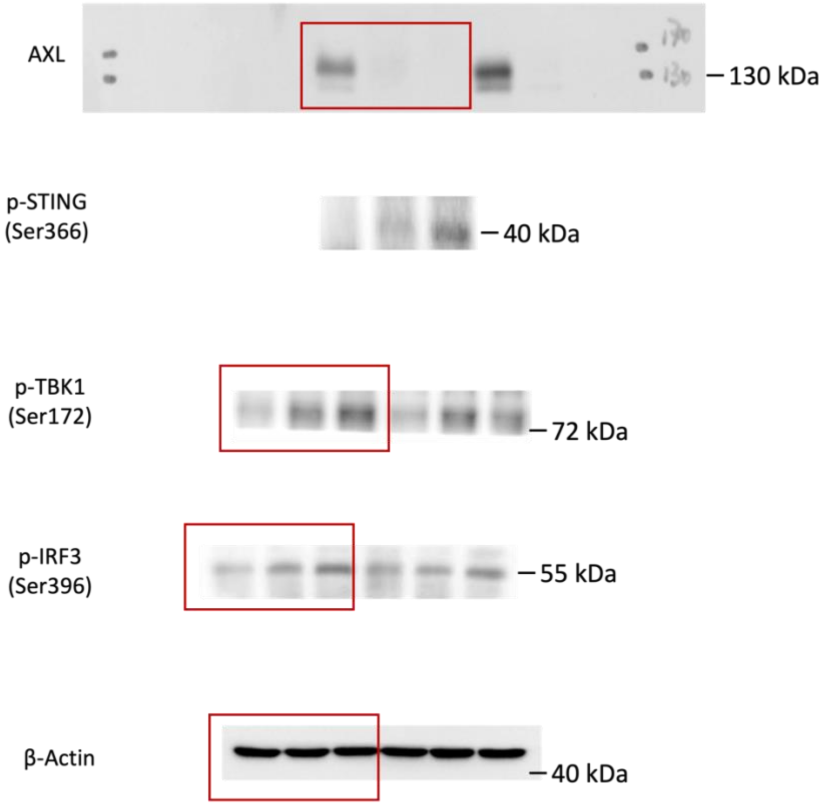

Supplementary Figure 7C

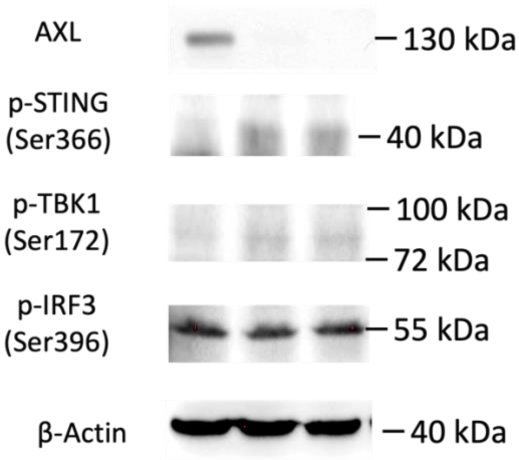

Supplementary Figure 8A

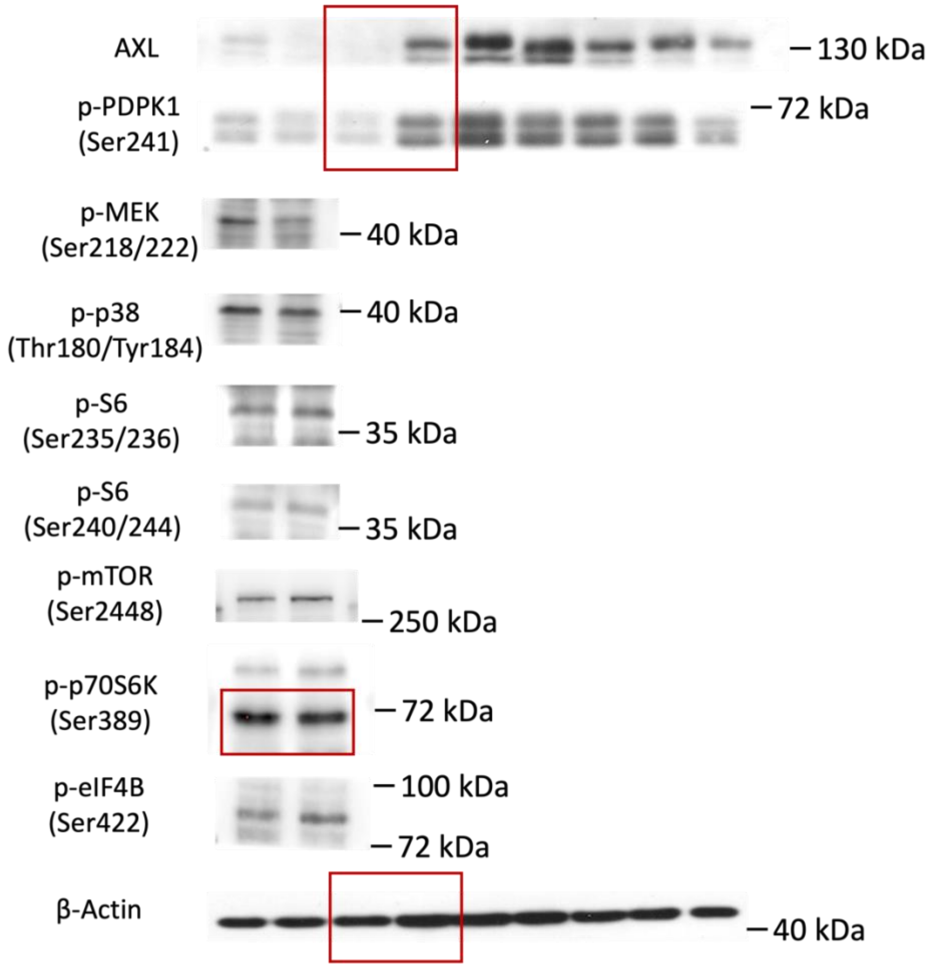

Supplementary Figure 8B

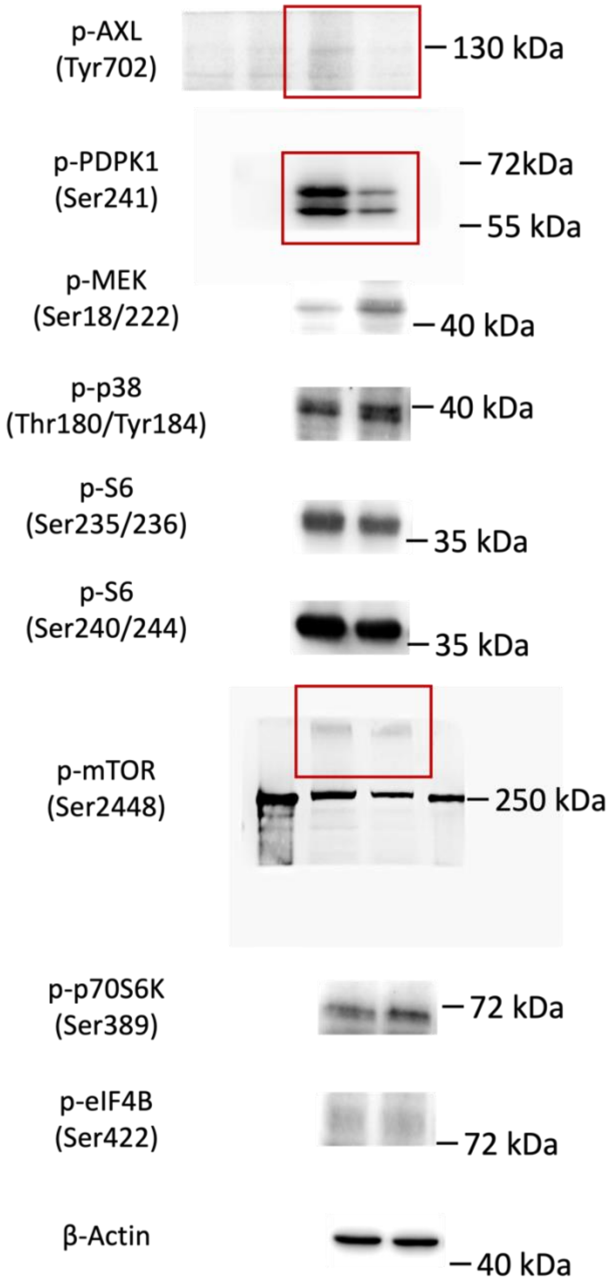

Supplementary Figure 8C

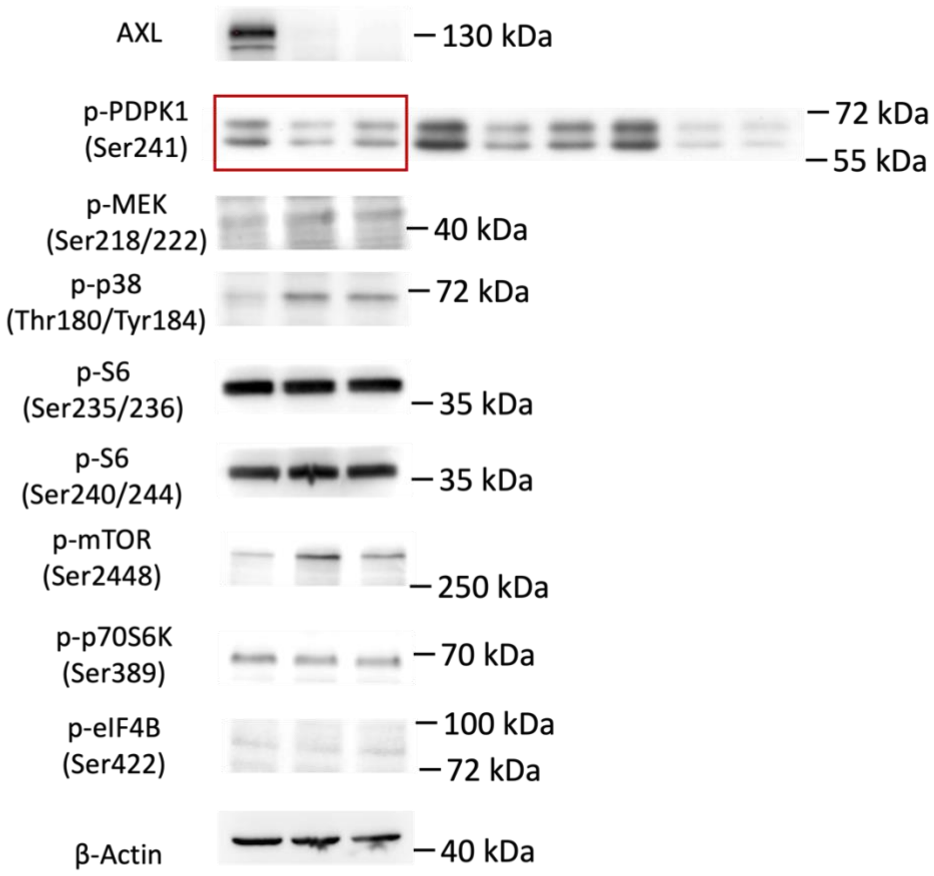

Supplementary Figure 9B

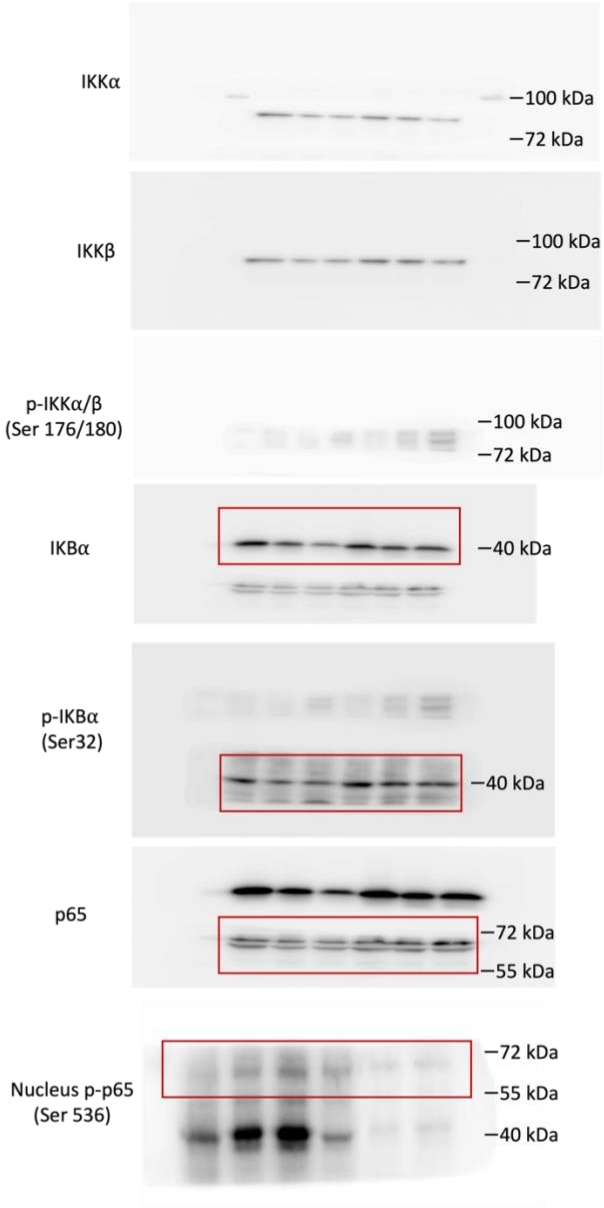

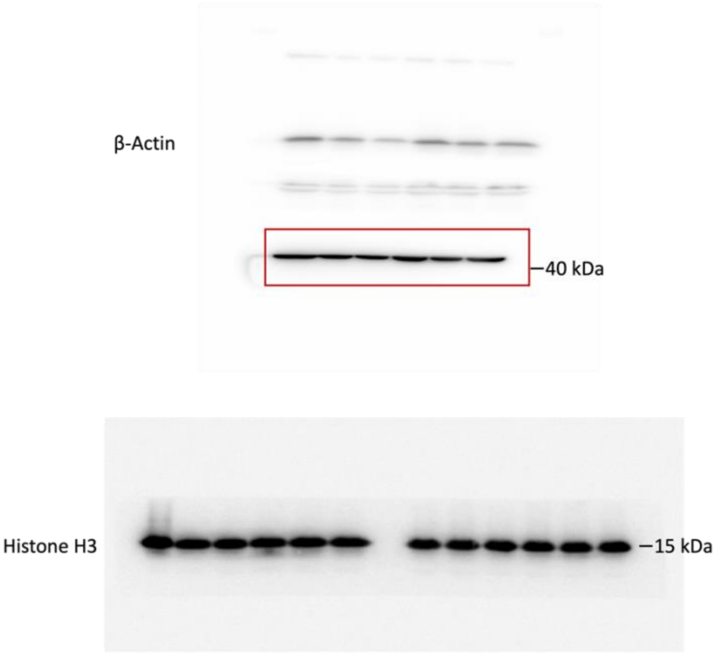

Supplementary Figure 9C

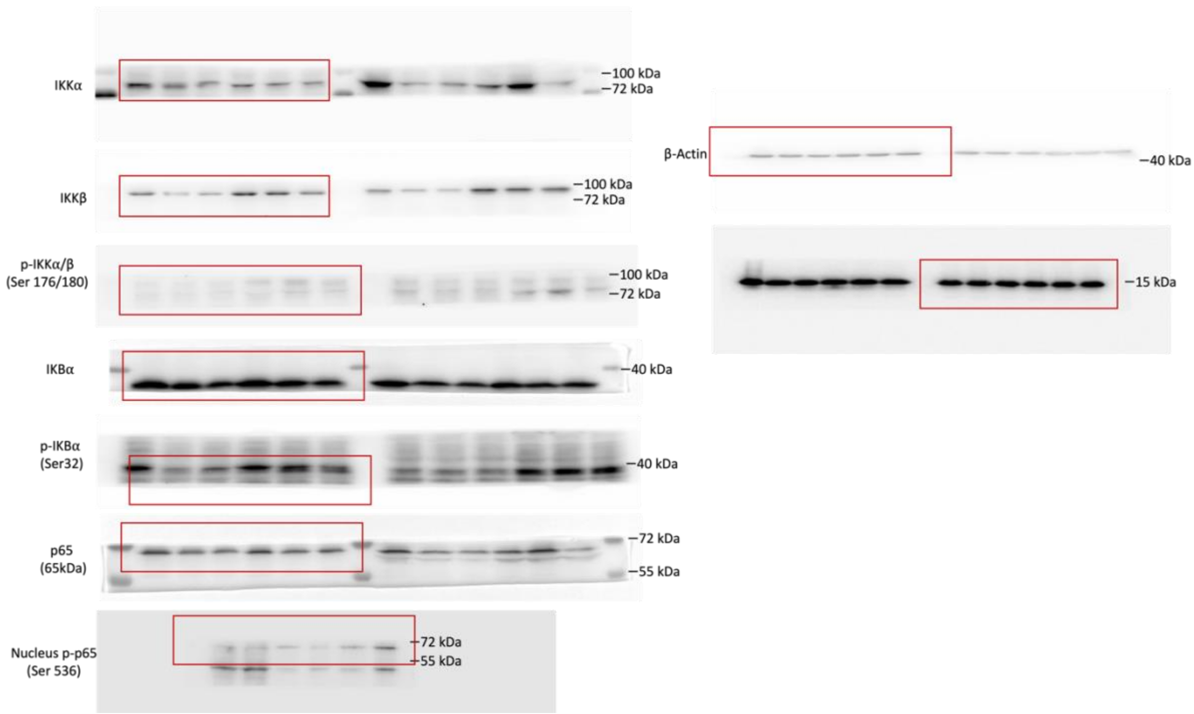

Supplementary Figure 9D

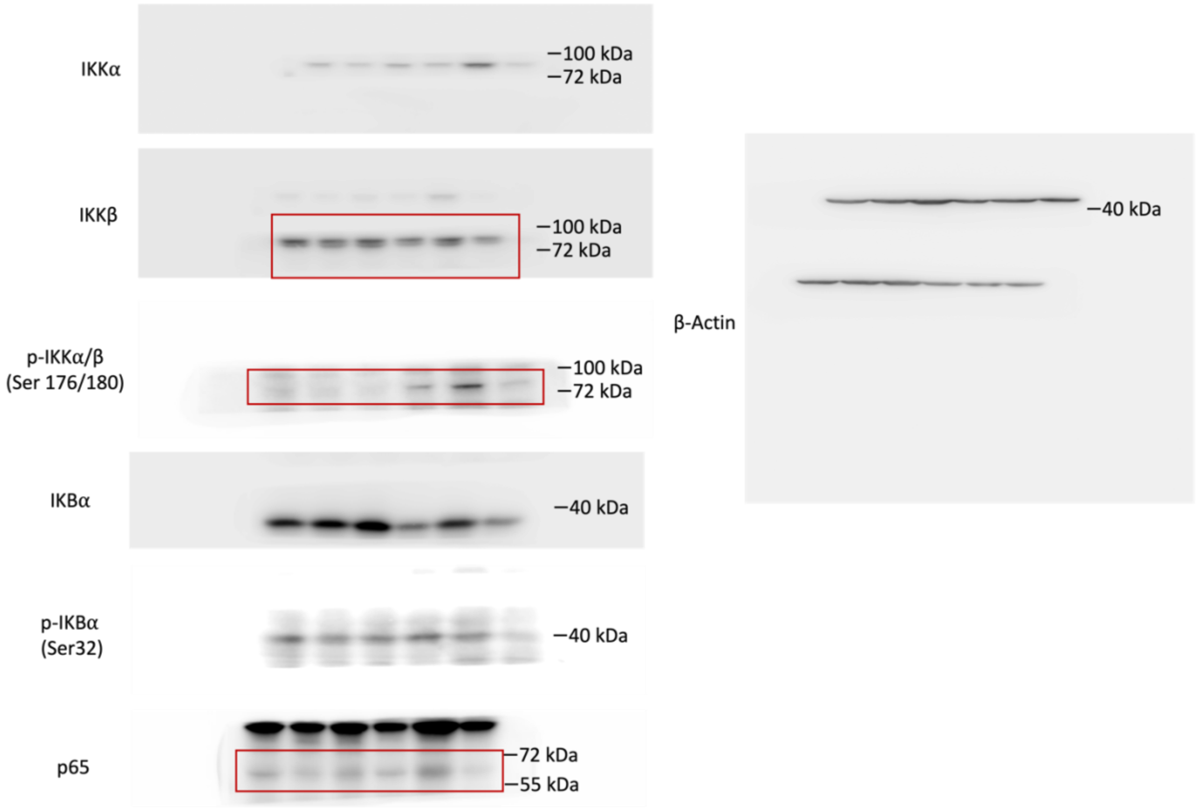

Supplementary Figure 10C

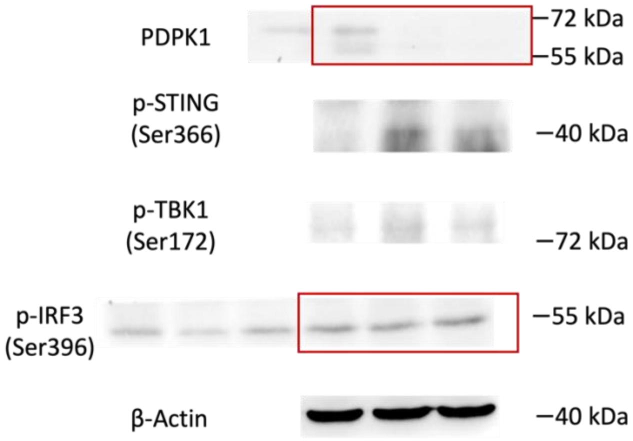

Supplementary Figure 11E

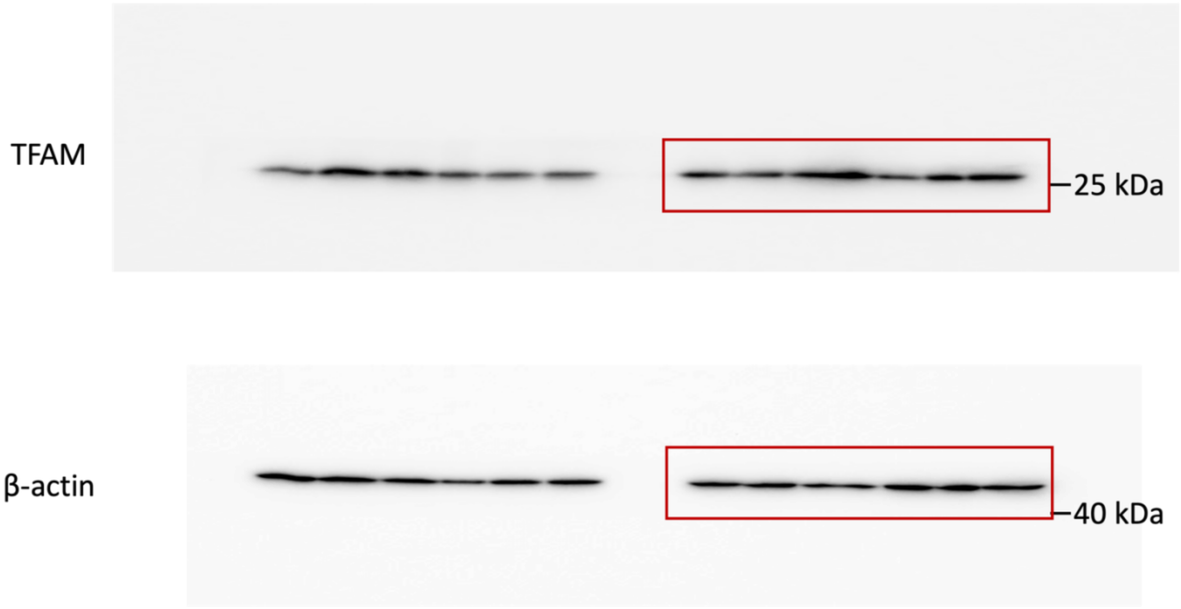

Supplementary Figure 11F

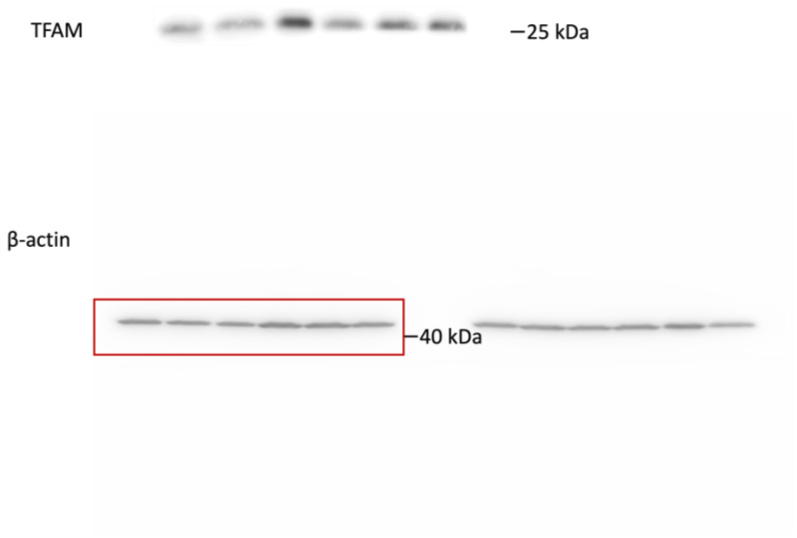

Supplementary Figure 11G

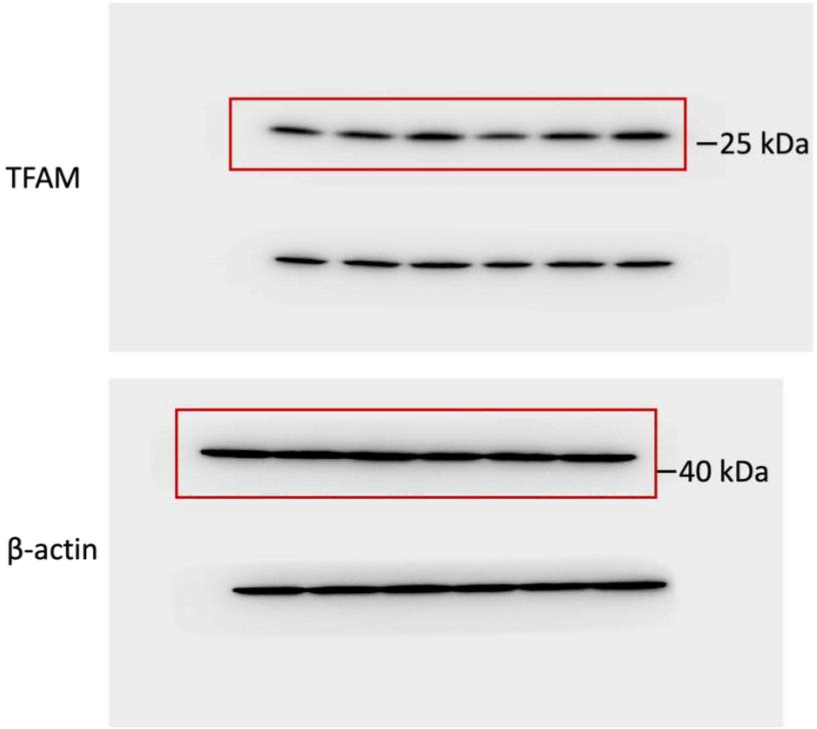

Supplementary Figure 12B

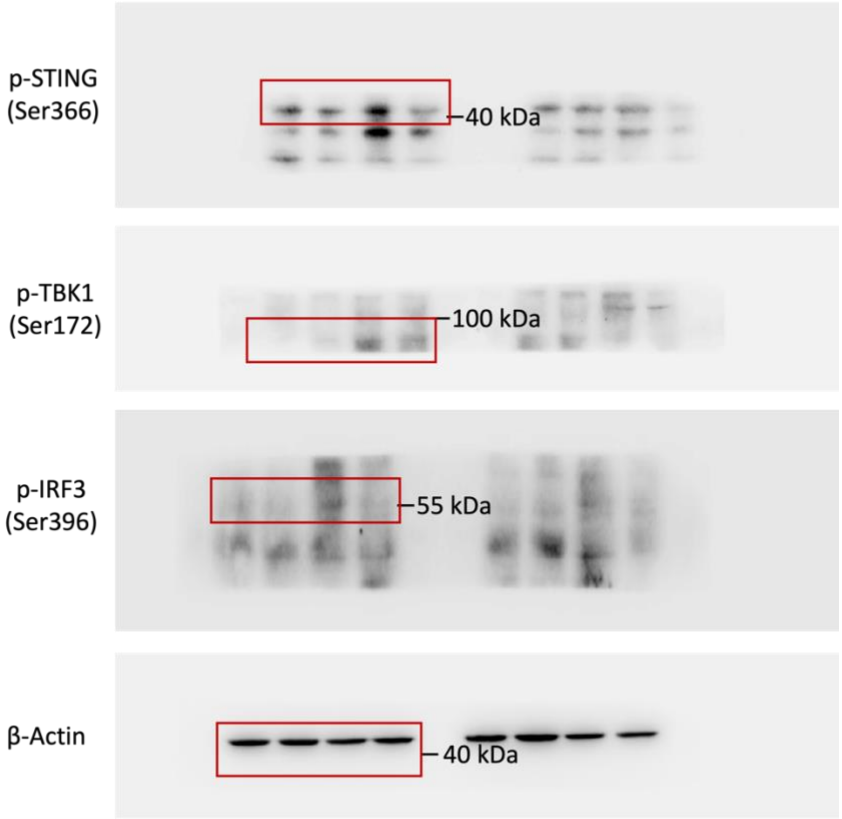

Supplementary Figure 12D

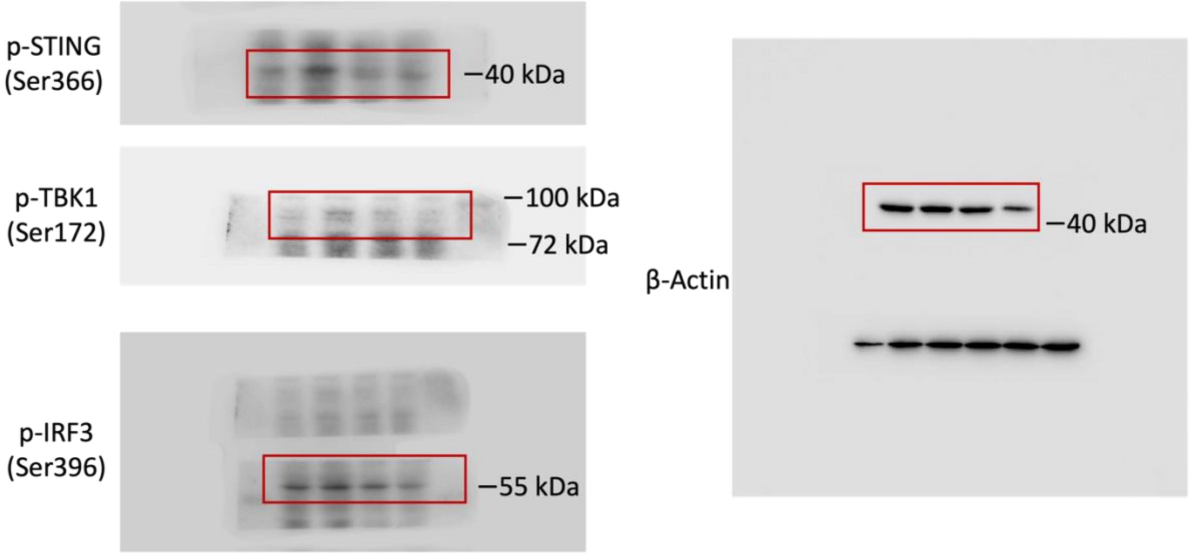

Supplementary Figure 13A

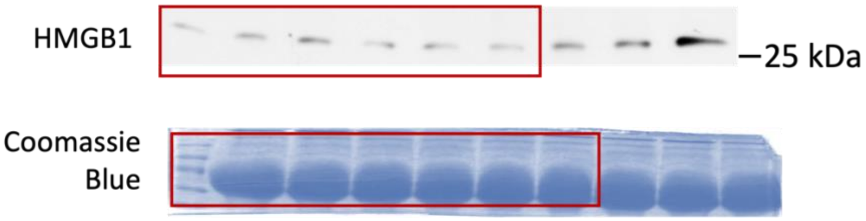

Supplementary Figure 14A

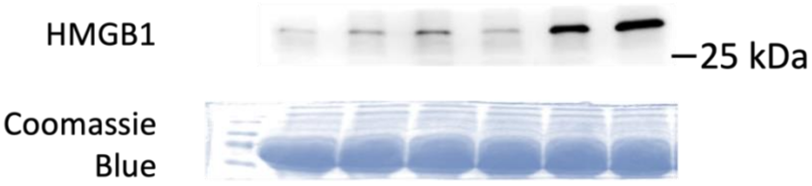

Supplementary Figure 14D

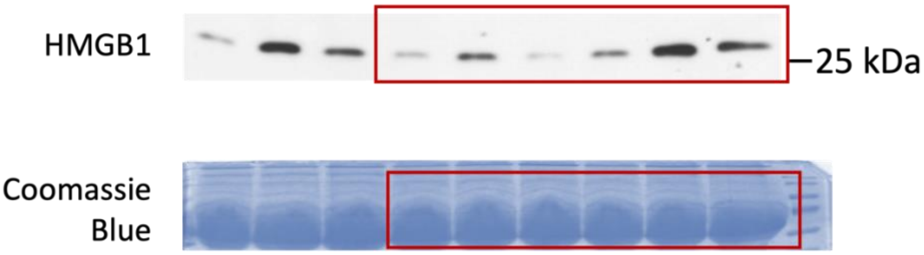

Supplement: Supplementary file 2 — Original Data File [file 41419_2024_6493_MOESM2_ESM.pdf]
